# Mitotic genes are transcriptionally upregulated in the fibroblast irradiated with very low doses of UV-C

**DOI:** 10.1038/srep29233

**Published:** 2016-07-05

**Authors:** Seiji Takeuchi, Toshiro Matsuda, Ryusuke Ono, Mariko Tsujimoto, Chikako Nishigori

**Affiliations:** 1 Division of Dermatology, Department of Internal Related, Kobe University Graduate School of Medicine, 7-5-1 Kusunoki-cho, Chuou-ku, Kobe 650-0017, Japan; 2 Kindai University Atomic Energy Research Institute, 3-4-1 Kowakae, Higashiosaka City, Osaka 577-8502, Japan

## Abstract

Ultraviolet (UV) radiation induces a variety of biological effects, including DNA damage response and cell signaling pathways. We performed transcriptome analysis using microarray in human primary cultured fibroblasts irradiated with UV-C (0.5 or 5 J/m^2^) and harvested at 4 or 12 h following UV exposure. All transcript data were analyzed by comparison with the corresponding results in non-irradiated (control) cells. The number of genes with significantly altered expression (≥2-fold difference relative to the control) is higher in the sample irradiated with high dose of UV, suggesting that gene expression was UV dose-dependent. Pathway analysis on the upregulated genes at 12 h indicates that the expression of some cell cycle-related genes was predominantly induced irrespective of UV-dose. Interestingly, almost all the genes with significant altered expression were cell cycle-related genes designated as ‘Mitotic Genes’, which function in the spindle assembly checkpoint. Therefore, even a low dose of UV could affect the transcriptional profile.

Ultraviolet (UV) radiation from sunlight is an environmental factor that has a variety of physiological and biological effects, including induction of DNA damage response pathways such as cell cycle checkpoint control, DNA repair and apoptosis. UV radiation (UVR) can be an environmental hazard and mutagen, leading to an increased risk of developing skin cancers, and these biological effects are mainly attributed to pyrimidine dimers, major DNA damage caused by UVR. UVR is classified into three bands according to the wavelength based on its characteristic biological effects: UV-A (320–400 nm), UV-B (280–320 nm), and UV-C (<280 nm). The major DNA damage produced by UV-C is pyrimidine dimers, while UV-B and UV-A produce both pyrimidine dimers and oxidative DNA lesions. UVR affects cellular DNA and the cell membrane, triggering a wide variety of signaling cascades to maintain homeostasis. Transcriptional regulation is one of the UVR-evoked signaling cascades, controlled in part by the balance between RNA synthesis and degradation. Thus, UVR-induced pathways as a protective responses such as cell cycle response and DNA repair can be roughly classified into two types, DNA damage-dependent pathway^1^ or DNA damage–independent pathway^2^.

Well known pathways activated by DNA damage include a p53-dependent G1-phase checkpoint^3^ and intra-S-phase ATR–CHK1 (ataxia telangiectasia and Rad3-related/checkpoint kinase 1)-dependent checkpoint^4^. Upon UV irradiation, ATR/CHK1 is activated, resulting in the phosphorylation of p53, leading to the transactivation of a variety of p53-responsive genes. The p53-responsive genes such as p21 play a role in cell cycle regulation and apoptosis in order to either sustain the UV-damaged cell or protect the UV-damaged individuals by inducing apoptosis of cells that are too severely damaged to be repaired. ATR kinase is activated in response to persistent single-stranded DNA and phosphorylates a variety of ATR-targeting genes such as CHK1^5^. Activation of ATR-targeting proteins contributes to the cell cycle checkpoint control and repair system mainly in the S-phase^6^,^7^.

In other cases UVR is known to induce the clustering of certain cell-surface receptors and leads to the formation of reactive oxygen species (ROS), resulting in the signal transduction of cell survival and proliferation^8^,^9^,^10^. One such DNA damage-independent pathway that has been extensively studied is the MAPK pathway that leads ultimately to survival of UV-irradiated cells^11^. The MAPK signaling pathways are-subdivided into three different pathways: the extracellular signal-regulated kinases (ERK), p38 MAPK (p38 kinase), and c-Jun NH2-terminal kinases (JNK) signaling pathways^12^. Previous reports suggest that induction of MAPK signaling pathways by UVR is wavelength-specific. UV-A induces stronger activation of ERK, whereas JNK is principally activated by UV-C and p38 kinases are activated in response to any UVR wavelength^11^,^13^.

DNA microarray technology has become a useful tool for the evaluation of global gene expression in UV-damaged cells. Several microarray analyses of UV-B-irradiated keratinocytes have yielded similar, almost identical, results in spite of considerable difference in experimental conditions^14^,^15^,^16^,^17^. Several papers on the microarray analysis of UV-B irradiated melanocytes, another cellular component of the epidermis, have also been published^18^,^19^,^20^. These studies were performed under the assumption of a physiological situation in which only UV-B and UV-A range of solar UVR reach the earth and UV-B cause the most typical sun-induced reaction such as sunburn and suntan. In one study, Yang *et al*. found that a set of p53 targets were deregulated in UV-B irradiated melanocytes^19^. UV-C has been used experimentally as an inducer of genetic mutations and cellular modifications. Gentile *et al*. reported that cell cycle arrest and apoptosis are transcriptionally highly distinct responses in the UV-C irradiated fibroblast^21^.

Solar UV-B and solar UV-A penetrate the epidermis, while UV-C does not^22^. Yet, in the present study we used UV-C to simplify the experimental condition to ask biological events caused by a small amount of pyrimidine dimers, a key molecule triggering biological effects, and they are also produced by UV-A and UV-B in human skin *in vivo* in daily life^23^. We irradiated fibroblasts with 0.5 J/m^2^ of UV-C, a dose 20 times lower than that which Gentile *et al*. applied as a “low” dose. We then performed microarray analysis of UV-irradiated and non-irradiated (control) samples and compared the data. To our surprise, despite the extremely low dose of UV-C, almost all the genes with significantly upregulated expression were found to be mitotic genes, which function in the spindle assembly checkpoint.

## Results

### Transcriptional profiles of affected genes in primary fibroblasts irradiated with a low dose of UV-C

Primary cultured fibroblasts obtained from a 30 y.o. female healthy volunteer were irradiated with a low dose of UV-C, and subjected to microarray analysis. Exponentially growing human skin fibroblast were irradiated with a low (0.5 J/m^2^) (LUV) or high (5 J/m^2^) (HUV) dose of UV-C and incubated for 4 or 12 h. The LUV (0.5 J/m^2^) used in this study is an extremely low dose that does not affect the survival of normal primary fibroblasts, as determined using colony formation assays^24^. Total RNA was extracted from the cells and was further processed and used in microarray hybridization as described in the Materials and Methods section. In order to screen the UV-C–affected genes specifically, transcriptional profiles of the UV-irradiated samples were compared with those of the non-irradiated samples. Genes that showed ≥2-fold difference were selected from filtered spots on the Flags (Table 1). Approximately 2% of all entities revealed altered expression in cells irradiated with LUV at two time points. On the other hand, in the samples irradiated with HUV, approximately 6% or 4% of all entities were altered at 4 or 12 h, respectively. This result suggests that the number of altered genes could be affected by the dose of UV.

**Figure 1:**
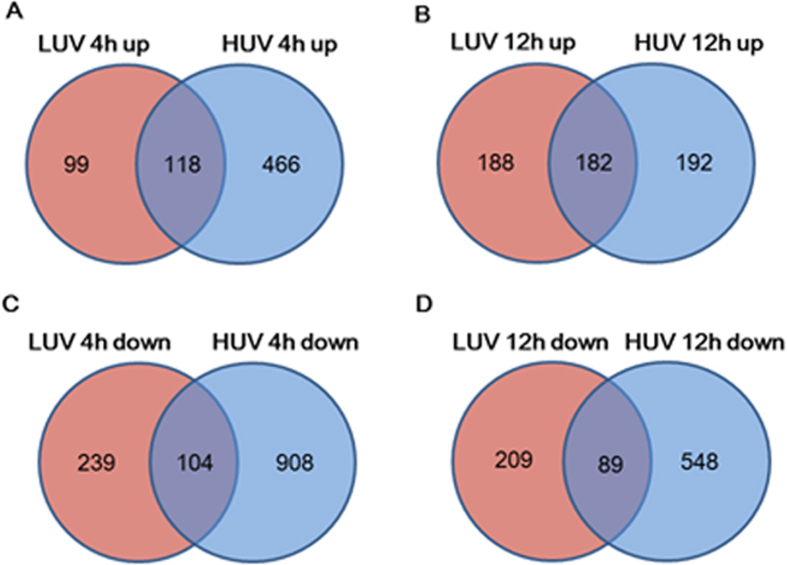
Venn diagram indicating the number of upregulated or downregulated genes following UV-C irradiation. The number of significantly altered genes (≥2-fold difference) at 4 or 12 h after UV exposure is indicated. LUV: lower dose of UV-C; 0.5 J/m^2^. HUV: higher dose of UV-C; 5 J/m^2^. The number of upregulated genes at 4 h (**A**) and at 12 h (**B**) is shown. The number of downregulated genes at (**C**) and at 12 h (**D**) is shown.

We also analyzed the overlapping genes between LUV- and HUV-induced genes as shown in Fig. 1 in the upregulated genes. The number of overlapping genes in the upregulated group at 4 h was 118 genes, whereas at 12 h, the number of overlapping genes was 182, as shown in Fig. 1A,B. The ratio of overlapping genes to total genes filtered on Flags at 4 h was 17% (118/683), whereas at 12 h, the ratio of overlapping genes to total genes was increased to 32% (182/562). In contrast to upregulated genes, in the downregulated genes the ratio of overlapping genes to total genes filtered on Flags at 4 h was 8% (104/1251), whereas, at 12 h, the ratio of overlapping genes was 10% (89/846). The ratio of overlapping genes between LUV and HUV in the upregulated group was higher than that of the downregulated one. The ratio markedly increased in a time-dependent manner in the upregulated genes group.

### Pathway analysis in the upregulated gene group

**Figure 2:**
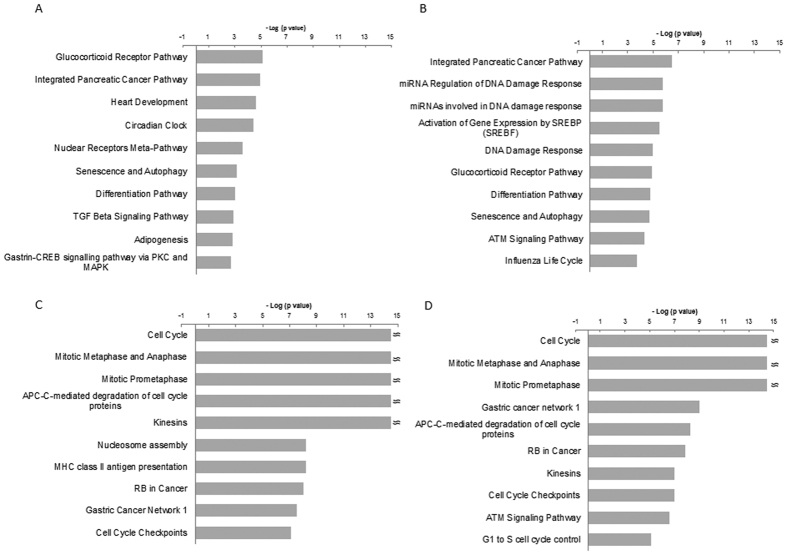
Top 10 pathways enriched for pathway analysis through WikiPathways in upregulated genes. The results from the pathway analysis through WikiPathways are depicted within the category of upregulated genes. (**A**) LUV, at 4 h (**B**) HUV, at 4 h (**C**) LUV, at 12 h (**D**) HUV, at 12 h. The Y-axis shows the minus logarithmic value of each p-value indicating the validity of each pathway. The accession number of each pathway for WikiPathways was saved; refer to the raw data in Supplementary Tables S1–4.

In order to evaluate the pathways of UV-affected genes, we used WikiPathways through Genespring software. The top 10 pathways of upregulated genes for each of the 4 samples are shown in Fig. 2 and raw data is shown in Supplementary Tables S1–S4. Interestingly, the pathways at 12 h in both the LUV and HUV samples clearly show a similar pattern, and included cell-cycle related pathways (Fig. 2C,D). In contrast, the pathways at 4 h (Fig. 2A,B) did not show such a characteristic pattern. The top 10 pathways at 4 h in the HUV samples include some DNA damage response-related genes, which were not evident in the pathways at 4 h in the LUV samples. The validities of the p-values in each pathway at 12 h are unambiguously higher than those at 4 h. These results suggest that the cell cycle pathways could be predominantly upregulated at the later time point after UV irradiation irrespective of UV dose. There were 8 overlapping pathways between the LUV and HUV samples at 12 h in the top 10 altered pathways; these included the Cell Cycle, Mitotic Metaphase and Anaphase, Mitotic Prometaphase, APC-C mediated degradation of cell cycle proteins, Kinesin, Cell Cycle Checkpoints, RB in cancer, and Gastric cancer network 1.

We also performed pathway analysis using GO (Gene Ontology) data set (Supplementary Tables S5–8). Consistent with the results from WikiPathways, in the 2 pathway profiles at 12 h (Supplementary Tables S7 and S8), cell cycle-related pathways, especially in the M phase, were clearly invoked. Among the pathways at 4 h in LUV samples, various pathways such as cellular response or cell signaling were found (Supplementary Tables S5 and S6). On the other hand, among the pathways at 4 h in HUV samples, nuclear and nucleolar component-related genes were indicated in the top level. Similar results obtained by the two forms of pathway analysis as described in WikiPathways and Gene Ontology validate our analysis.

### Pathway analysis in the downregulated gene group

**Figure 3:**
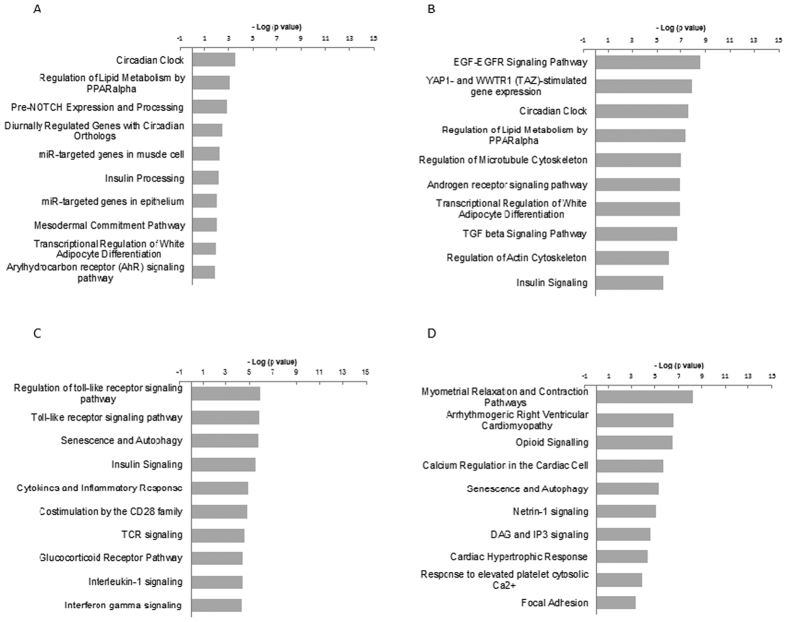
Top 10 pathways enriched for pathway analysis through WikiPathways in downregulated genes. The results from the pathway analysis through WikiPathways are depicted within the category of downregulated genes. (**A**) LUV, at 4 h (**B**) HUV, at 4 h (**C**) LUV, at 12 h (**D**) HUV, at 12 h. The Y-axis shows the minus logarithmic value of each p-value indicating the validity of each pathway in the same manner as in Fig. 2. The accession number of each pathway for WikiPathways was saved; refer to the raw data in Supplementary Tables S9–12.

We also conducted pathway analysis on the downregulated genes (Fig. 3A–D, Supplementary Tables S9–S12). In contrast to the upregulated gene group, characteristic pathways such as cell cycle-related pathways were not induced, even in the samples at 12 h. Comparing the HUV and LUV sample top 10 pathways at 4 h, there were three overlapping pathways: Circadian Clock, Regulation of Lipid Metabolism by PPARalpha, and Transcriptional Regulation of White Adipocyte Differentiations. The most significantly downregulated pathway in the HUV sample at 4 h was the EGF-EGFR Signaling pathway (Fig. 3B), in which only one gene was significantly downregulated in the LUV sample (Supplementary Table S11), suggesting that this pathway could be downregulated by the relatively higher amount of UV dose. In the two groups at 12 h, there was one overlapping pathway, Senescence and Autophagy. In fact, similar genes were changed, comparing two groups at individual gene level (Supplementary Fig. S1).

### Changes in expression of cell cycle-related genes in fibroblasts following UV-C irradiation

**Figure 4:**
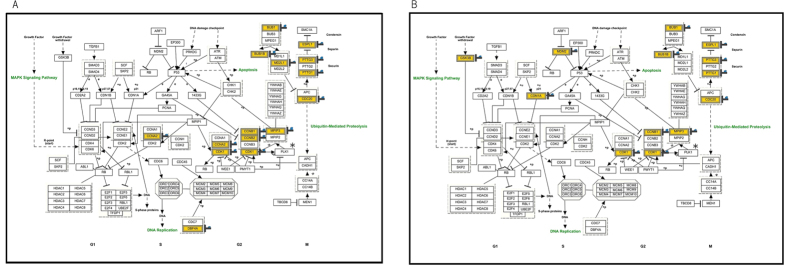
Mitotic genes are predominantly induced for the cell cycle-related genes at 12 h following UV exposure. The significantly upregulated genes within Cell Cycle (WP179_70629) are depicted in the case of LUV (**A**) and HUV (**B**). The brown columns show the significantly upregulated genes (≥2-fold difference). *PLK1 was filtered out by insufficient flag value in the un-irradiated sample for significantly upregulated genes despite the significant upregulation of PLK1 in both HUV and LUV at 12 h in the Supplementary Fig. S2.

Multiple cell cycle-related pathways were markedly upregulated in fibroblasts exposed to HUV as well as those exposed to LUV at 12 h. In order to elucidate which genes were upregulated, the picture of Cell Cycle Pathway (WP179_70629) offered to the WikiPathways were depicted in Fig. 4. Upregulated genes in samples exposed to HUV or LUV indicates a similarity in the response pattern between the two samples. The upregulated genes were unevenly distributed and represented genes potently functioning from G2 to M, implying that these genes might be functional in the cell cycle regulation from G2 to M. We found that 10 genes for significantly upregulated genes commonly found in both LUV and HUV samples at 12 h, which were BUB1, BUB1B, CCNB1, CDC20, CDK1, ESPL1, MPIP3, PLK1, PTTG1, PTTG3 (Fig. 4, Supplementary Fig. S2). PLK1 was filtered out by insufficient flag value in the un-irradiated sample for significantly upregulated genes. We recognized PLK1 as one of significantly upregulated genes at 12 h. Surprisingly, all of these genes have been estimated to function in mitosis and/or at the spindle assembly checkpoint (SAC) and are designated as ‘Mitotic Genes’.

In order to validate the results from microarray analysis, we performed qRT-PCR on the three genes selected from mitotic genes described above. At 12 h after UV exposure, expressional induction could be found on all three genes, consistent with the results from microarray analysis (Supplementary Fig. S4).

## Discussion

We found that a much lower dose of UV-C than is commonly used (LUV) could induce the characteristic pathways, which are mainly composed of cell cycle-related ones at 12 h after UV irradiation. In the HUV at 12 h, similar genes and pathways to LUV case were induced, comparing transcriptional profiles between HUV and LUV. Cell cycle-related genes showing significant upregulation (≥2-fold difference) at 12 h in both LUV and HUV include BUB1, BUB1B, CCNB1, CDC20, CDK1, ESPL1, MPIP3, PLK1, PTTG1, PTTG3, all of which are designated as ‘mitotic genes’, functioning in SAC (Fig. 4, Supplementary Fig. S2). On the other hand, at 4 h, multiple DNA damage response pathways were identified among the top 10 known pathways in samples at 4 h following exposure to HUV, in contrast to that observed in the LUV samples (Fig. 2). There are many reports demonstrating that UV irradiation damages genomic DNA, evoking a variety of DNA damage response pathways such as the p53 and ATM/ATR pathways. In fact, p21 and MDM2, which are well-known p53-transactivated genes^25^,^26^, were significantly upregulated at 4 h in the HUV sample (Supplementary Fig. S3). Downstream targets of the ATM/ATR pathway such as CDC25A and H2AFX as well as ATM itself are also transcriptionally induced in the HUV sample group at 4 h. These results indicate that the p53 and ATM/ATR pathway could be activated in the HUV sample, whereas induction of DNA damage response pathways might be obscure in LUV.

We should note that the features of individual cell cycle-related genes could function mainly in the M phase. These genes have been characterized as ‘Mitotic Genes’ which are directly involved in mitosis itself and/or the SAC. The SAC is a mechanism employed by cells to avoid genomic instability, regulating metaphase to anaphase transition by ensuring the fidelity of chromosome segregation^27^. The SAC signaling converges on Cdc20, a key activator of the ubiquitin E3 ligase anaphase-promoting complex/cyclosome (APC/C) that targets cyclin B and securin for proteolysis^27^,^28^. It has been reported that UV-C exposure can delay mitotic progression through an alternative mechanism to DNA damage response^29^. The induction of many mitotic genes following UV exposure may result in the delay of mitotic progression via activation of the SAC. It has been reported that transcriptional regulation of mitotic genes is coordinated by several transcription factors, NF-Y^30^, Forkbox M1^31^ and E2F^32^. Whether these transcription factors can be activated by UV-C irradiation and also be linked to the induction of mitotic genes has, to our knowledge, not been defined. Expression of cyclin B, a Cdk1 activator involved in G2/M progression, has been found to be regulated by p53^33^. Direct interaction of p53 with the promoter response element downregulates cyclin B expression upon DNA damage-mediated checkpoint arrest. Furthermore, it has been reported that p53 controls the transcription of CDC20 and BUB1B^34^,^35^,^36^. Following genotoxic stress, Cdc20 expression is indirectly suppressed by p53 through a p21-dependent mechanism^34^. Based on our findings that mitotic genes are induced by UV irrespective of UV dose, p53 activation is unlikely to be responsible for the induction of the expression of mitotic genes.

In the case of downregulated pathways at 4 h, common pathways between LUV and HUV samples in the top 10 are Circadian Clock, Regulation of Lipid Metabolism by PPARalpha and Transcriptional Regulation of White Adipocyte Differentiation. In the case of the 12 h time point, only Senescence and Autophagy overlap between LUV and HUV among the top 10 pathways. In contrast to the upregulated pathways, we did not find any characteristic downregulated pathways other than those described above. A possible explanation for this is that UV-induced photoproducts could arrest the elongation of RNA polymerase II, possibly inhibiting the synthesis of transcripts throughout the genome^37^. The pathway of downregulated genes may be reflected by the stalling of RNA polymerase II at the damaged site, consequently, the downregulated pathways does not include particular pathways in contrast to the case of upregulated genes, where ‘Mitotic Genes’ are predominantly induced.

In conclusion, we found that an extremely lower dose of UV-C than that is commonly used in similar studies could induce a change in the expression of many genes. Notably ‘Mitotic Genes’ were unambiguously induced to an equivalent extent as that observed with a higher dose of UV. Using microarray analysis, we identified transcription control pathways in the SAC following UV irradiation. Recently, the importance of this pathway has been indicated^38^. Although the exact significance of upregulation of mitotic genes after very small amount UV-C remains to be elucidated, it could be possible that upon even a small amount of pyrimidine dimers triggers the preparation for the high fidelity mitosis.

## Methods

### Cell culture and UV irradiation

The study was approved by Ethics Committee of Kobe University School of Medicine and conducted according to the Declaration of Helsinki. All the experiments described here were performed in accordance with the regulations and guidelines issued by the Ethics Committee of Kobe University School of Medicine.

Primary cultures of skin fibroblasts were established from a healthy volunteer as previously described^39^. The volunteer gave the informed consent. This method was approved by Kobe University School of Medicine Ethics Committee. Cells were maintained in Dulbecco’s modified Eagle medium (DMEM) supplemented with 10% fetal bovine serum. The cells were incubated at 37 °C in a humidified atmosphere containing 5% CO_2_. Exponentially growing fibroblasts were irradiated with UV-C using a germicidal lamp (Toshiba GL10) at 0.5 J/m^2^ as low dose UV (LUV) or 5 J/m^2^ as high dose UV (HUV). Fluence rate was 100 mW/m^2^, measured using a UV radiometer UV-254 (Topcon, Tokyo Kogaku Kikai KK, Tokyo, Japan). The medium in the cell culture were removed and rinsed with PBS one time before the cells were exposed with UV-C. Immediately after UV exposure, the cell culture were refilled with medium and incubated in the incubator. At 4 or 12 h time points after UV exposure, cells were harvested using a cell scraper.

### cRNA preparation, array hybridization, and scanning

Harvested cells were lysed with Trizol™ (Ambion) and total RNA was purified according to the manufacturer’s protocol. The quality of total RNA was verified using an Agilent 2100 Bioanalyzer. Biotinylated cRNA was prepared according to the standard Affymetrix protocol starting with 250 ng of total RNA and using the GeneChip 3′ IVT Express Kit. Following fragmentation, 10 μg of cRNA were hybridized for 16 h at 45 °C on a Human Genome U133 Plus 2.0 Array. GeneChips were washed and stained under the control of the Affymetrix GeneChip Command Console Software (AGCC). GeneChips were scanned using the Laser scanner. The data were analyzed with Microarray Suite version 5.0 (MAS 5.0) using expression console software.

### Data analysis

The raw data from all analyses performed are available at the Gene Expression Omnibus (http://www.ncbi.nlm.nih.gov/geo/) by accession numbers GSE70818 for low dose and high dose data series. Microarray data were normalized and analyzed using GeneSpring 13.0 (Agilent Technologies). Each entities was filtered based on their flag values (Present or Marginal). Per Chip normalization to the 75 percentile shift was performed. Changes in gene expression were determined by comparing all treatments with the non-irradiated control. Differentially expressed genes were determined based on a fold change cutoff of at least 2 (increase or decrease). Pathway analysis and gene ontology (GO) analysis were also performed GeneSpring 13.0. Differentially expressed gene lists were subjected to pathway analysis through the Simple Experiment Analysis (SEA) workflow using WikiPathways database.

### qRT-PCR

At 12 h after UV-C exposure (0.5 J/m^2^, 5 J/m^2^), harvested cells were lysed with TrizolTM (Ambion) and total RNA was purified according to the manufacturer’s protocol. RNA concentration and purity were assessed using NanoPhotometer (Implen). RNA was reverse-transcribed with PrimeScriptTM RT Master Mix (TAKARA). qRT-PCR was performed using a Applied Biosystems 7500 Real-Time PCR System (ThermoFisher Scientific) and SYBR Premix Ex TaqTM II (TAKARA) as described by the manufacturer. The endogenous expression of glyceraldehyde-phosphate dehydrogenase (GAPDH) was used as the internal control. All primers for detected genes were described in Supplementary Table S13.

## Additional Information

**How to cite this article**: Takeuchi, S. *et al*. Mitotic genes are transcriptionally upregulated in the fibroblast irradiated with very low doses of UV-C. *Sci. Rep.* **6**, 29233; doi: 10.1038/srep29233 (2016).

## Supplementary Material

Supplementary Information

## Figures and Tables

**Table 1:** The number of genes with changed expression when irradiated with the lower dose of UV-C.

Sample*	UV-C dose (J/m^2^)	Harvest time (hr)	All Entities**	Filtered on Flags***	Fold change > = 2.0	Upregulated	Downregulated
LUV-4 h	0.5	4	54675	24858	560	217	343
HUV-4 h	5	4	54675	24398	1596	584	1012
LUV-12 h	0.5	12	54675	25294	668	370	298
HUV-12 h	5	12	54675	24977	1011	374	637

^*^In each sample, the genes indicating the significant expressional difference were picked up compared with that in un-irradiated cell.

^**^Total number of the spots as targets on expression analysis on microarray.

^***^The number of spots indicating Present or Marginal fluorescent intensities.
