# Supplementary material for: Mitotic genes are transcriptionally upregulated in the fibroblast irradiated with very low doses of UV-C
Source: Sci Rep. 2016 Jul 5;6:29233. doi: 10.1038/srep29233 (PMC4932599; doi:10.1038/srep29233)
Supplement: Supplementary Information [file srep29233-s1.pdf]

Supplementary information file

Mitotic genes are transcriptionally upregulated in the fibroblast irradiated with very low doses of UV-C.

Seiji TAKEUCHI[1†], Toshiro MATSUDA[2†], Ryusuke ONO[1], Mariko TSUJIMOTO[1], Chikako NISHIGORI[1]

[1]Division of Dermatology, Department of Internal Related, Kobe University Graduate School of Medicine, [2]Kindai University Atomic Energy Research Institute

[†]These authors contributed equally to this work

Corresponding author: Chikako Nishigori

Division of Dermatology, Graduate School of Medicine, Kobe University

7-5-1, Kusunoki-cho, Chuou-ku, Kobe 650-0017, Japan

Tel: 81-78-382-6134

Fax: 81-78-382-6149

Correspondence to chikako@med.kobe-u.ac.jp

A

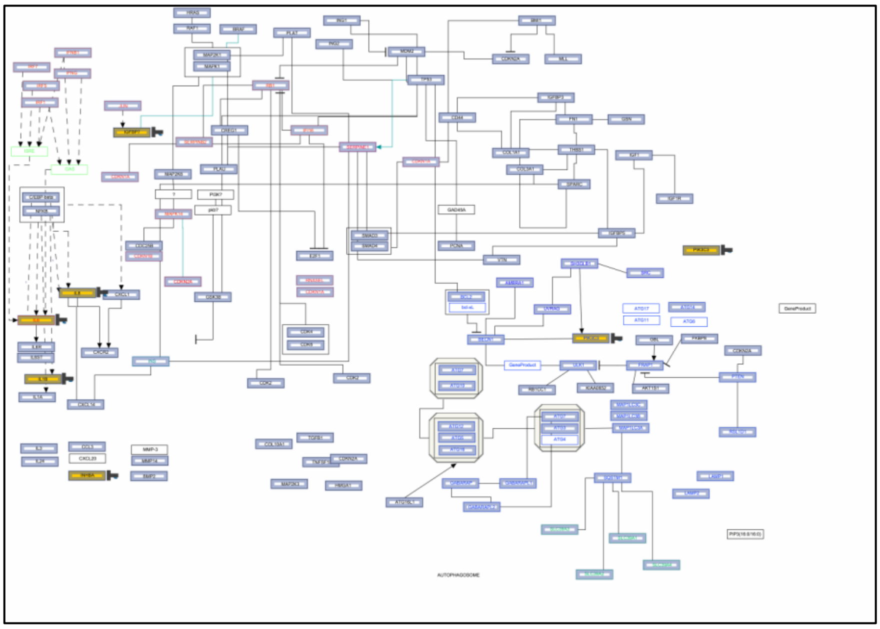

B

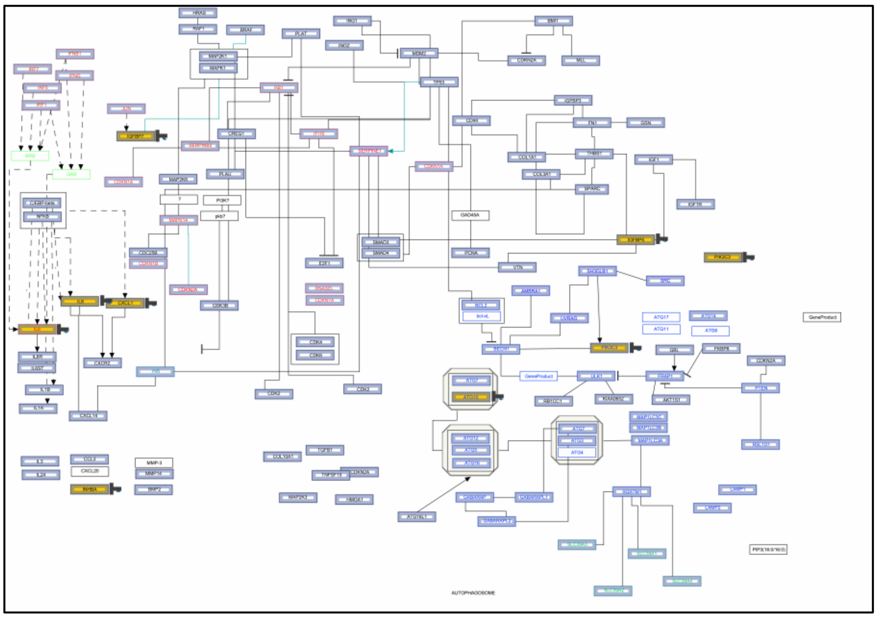

Figure S1

Significantly downregulated genes in Senescence and Autophagy (WP615_71375) from Wikipathways

The significantly downregulated genes within Senescence and Autophagy (WP615_71375) are depicted in the case of LUV (A) and HUV (B) at 12 h. The brown columns show the significantly upregulated genes (≥2-fold difference).

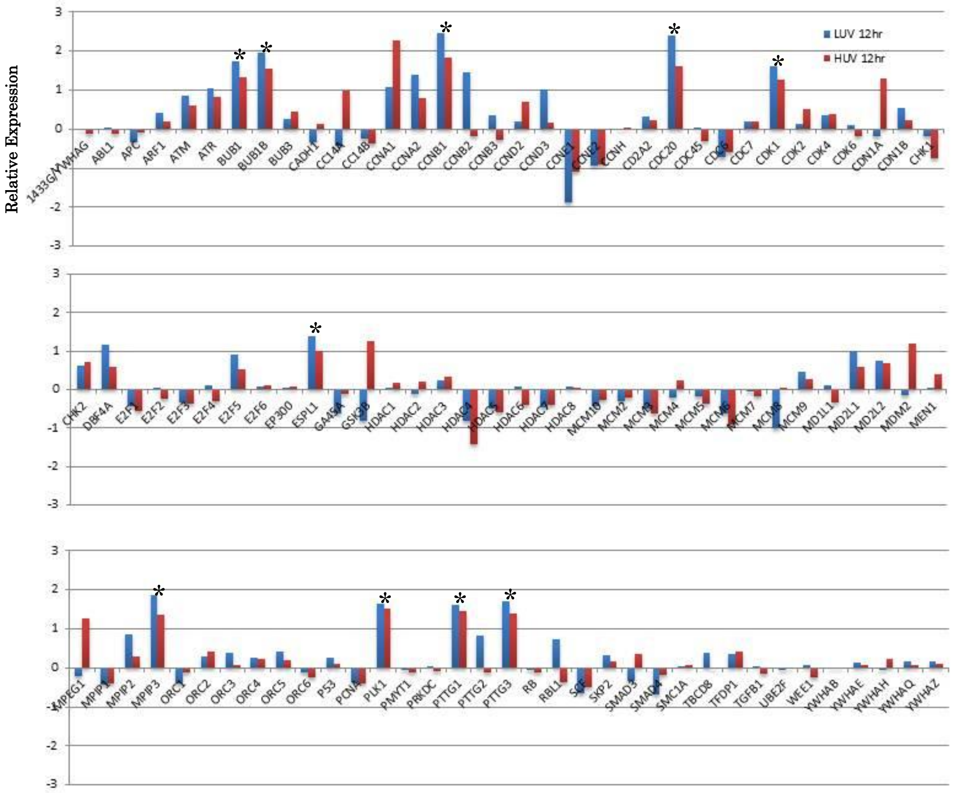

Figure S2

Relative gene expression in Cell Cycle Pathway (WP179_70629) at 12 h after UV-C exposure.

Asterisk-attached genes indicate that the expression level were significantly upregulated (2≥-fold difference relative to control) commonly in both LUV and HUV sample.

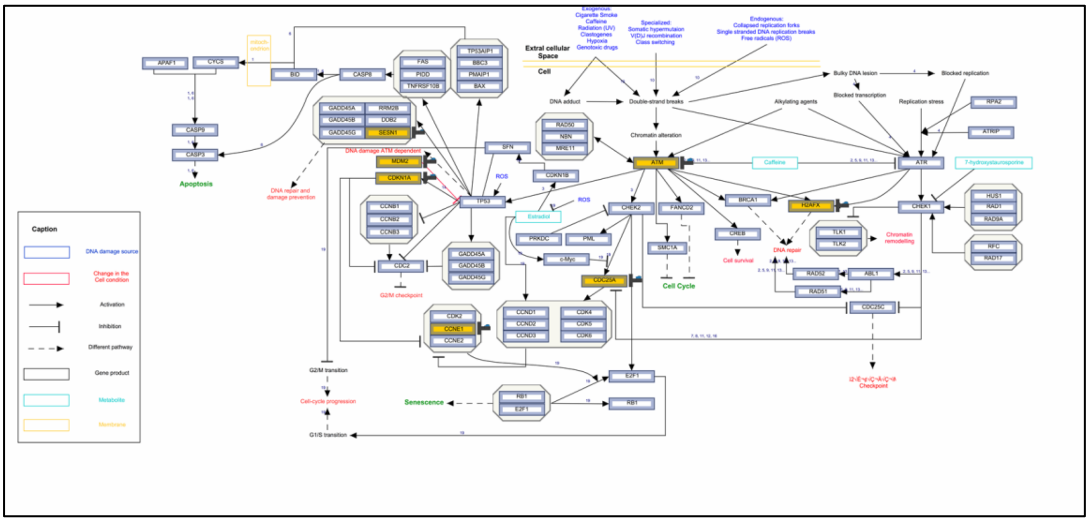

Figure S3

Siginificantly upregulated genes in DNA Damage Response (WP707_78527) from Wikipathways

The significantly upregulated genes within DNA Damage Response (WP707_78527) are depicted in the HUV (B) at 4 h. The brown columns show the significantly upregulated genes (≥2-fold difference).

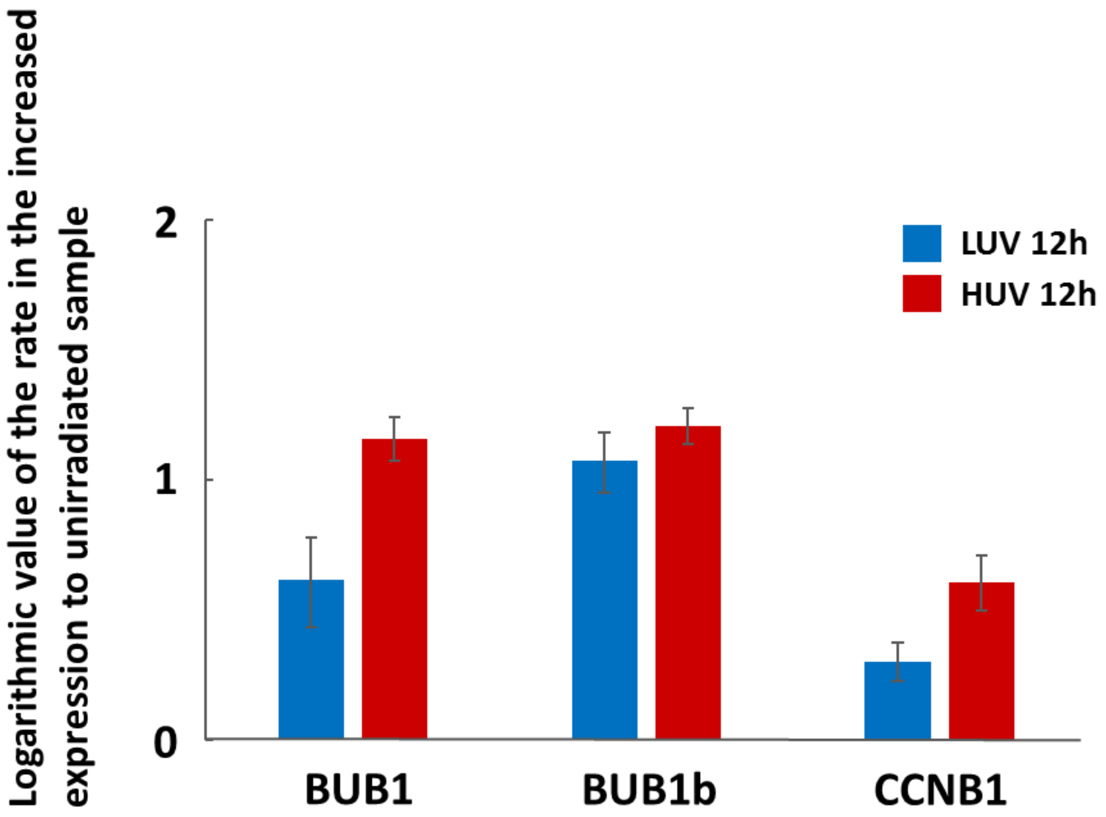

Figure S4

Three mitotic genes were upregulated, validated by qRT-PCR

The histogram vertical axis indicates that logarithmic value of the rate in the increased expression to unirradiated sample.

Table S1 Pathway analysis through WikiPathways in the fibroblast irradiated with UV-C (upregulation, 4hr, 0.5J/m^2)	p-value (low dose 4hr)	Matched Entities (low dose 4hr)	Pathway Entities of Experiment Type (low dose 4hr)
Hs_Glucocorticoid_Receptor_Pathway_WP2880_79522	8.15E-06	5	71
Hs_Glucocorticoid_Receptor_Pathway_WP2880_79615	8.15E-06	5	71
Hs_Integrated_Pancreatic_Cancer_Pathway_WP2377_71228	1.38E-05	7	200
Hs_Heart_Development_WP1591_78590	2.62E-05	4	47
Hs_Circadian_Clock_WP1797_76871	4.03E-05	4	49
Hs_Nuclear_Receptors_Meta-Pathway_WP2882_78569	2.36E-04	7	318
Hs_Senescence_and_Autophagy_WP615_71375	7.95E-04	4	106
Hs_Differentiation_Pathway_WP2848_78558	9.23E-04	3	50
Hs_TGF_Beta_Signaling_Pathway_WP560_68944	0.001225039	3	55
Hs_Adipogenesis_WP236_78584	0.001691314	4	131
Hs_Gastrin-CREB_signalling_pathway_via_PKC_and_MAPK_WP2664_76844	0.001993909	4	147
Hs_Endochondral_Ossification_WP474_72122	0.002000213	3	64
Hs_Oncostatin_M_Signaling_Pathway_WP2374_73668	0.002091067	3	65
Hs_Insulin_Signaling_WP481_72080	0.003659608	4	161
Hs_SIDS_Susceptibility_Pathways_WP706_78533	0.003823773	4	166
Hs_MAPK_Signaling_Pathway_WP382_72103	0.004255859	4	168
Hs_Elastic_fibre_formation_WP2666_76849	0.006849377	2	32
Hs_Interleukin-1_signaling_WP1839_76943	0.009081217	2	38
Hs_Transcriptional_activity_of_SMAD2-SMAD3-SMAD4_heterotrimer_WP2755_77005	0.011592632	2	44
Hs_IL-6_signaling_pathway_WP364_78561	0.012127451	2	43
Hs_Interleukin-11_Signaling_Pathway_WP2332_79525	0.012672903	2	44
Hs_Structural_Pathway_of_Interleukin_1_(IL-1)_WP2637_76330	0.014372171	2	49
Hs_Structural_Pathway_of_Interleukin_1_(IL-1)_WP2637_79580	0.014372171	2	49
Hs_Serotonin_Receptor_2_and_STAT3_Signaling_WP733_74441	0.015392281	1	4
Hs_Complement_and_Coagulation_Cascades_WP558_67786	0.01678137	2	64
Hs_Regulation_of_toll-like_receptor_signaling_pathway_WP1449_77378	0.018011875	3	150
Hs_Regulation_of_toll-like_receptor_signaling_pathway_WP1449_79550	0.018346587	3	150
Hs_IL-1_signaling_pathway_WP195_78528	0.0193495	2	55
Hs_Extracellular_matrix_organization_WP2703_76914	0.02137664	2	58
Hs_miR-targeted_genes_in_lymphocytes_-_TarBase_WP2004_78524	0.022405311	5	495
Hs_Complement_and_Coagulation_Cascades_WP558_79680	0.022774933	2	61
Hs_EGF-EGFR_Signaling_Pathway_WP437_79266	0.025362847	3	162
Hs_Interleukin-6_signaling_WP2704_76915	0.026781576	1	11
Hs_Endoderm_Differentiation_WP2853_78496	0.028729012	2	146
Hs_Dissolution_of_Fibrin_Clot_WP1802_76989	0.0305488	1	8
Hs_Non-homologous_end_joining_WP438_68977	0.0305488	1	8
Hs_L1CAM_interactions_WP1843_76876	0.031105012	2	75
Hs_Transcriptional_Regulation_of_White_Adipocyte_Differentiation_WP2751_76992	0.032730736	2	74
Hs_Mammary_gland_development_pathway_-_Involution_(Stage_4_of_4)_WP2815_78062	0.038039774	1	10
Hs_Signaling_by_VEGF_WP1919_76864	0.038039774	1	10
Hs_Selenium_Micronutrient_Network_WP15_78776	0.039554447	2	84
Hs_miR-targeted_genes_in_muscle_cell_-_TarBase_WP2005_78538	0.041500688	4	409
Hs_Mitotic_G2-G2-M_phases_WP1859_77022	0.044992782	2	89
Hs_BMP_Signalling_and_Regulation_WP1425_74390	0.04547315	1	12
Hs_Peptide_hormone_biosynthesis_WP2691_76894	0.04547315	1	12
Hs_Cell_surface_interactions_at_the_vascular_wall_WP1794_77039	0.045924645	2	91
Hs_GPCR_ligand_binding_WP1825_76977	0.04863014	4	371
Hs_Platelet_Adhesion_to_exposed_collagen_WP1883_76862	0.049168378	1	13
Hs_SRF_and_miRs_in_Smooth_Muscle_Differentiation_and_Proliferation_WP1991_75261	0.049168378	1	17
Hs_Signaling_by_Activin_WP2791_77082	0.049168378	1	13
Hs_Quercetin_and_Nf-kB-_AP-1_Induced_Cell_Apoptosis_WP2435_79583	0.05284937	1	15
Hs_Quercetin_and_Nf-kB-_AP-1_Induced_Cell_Apoptosis_WP2435_79692	0.05284937	1	15
Hs_Regulation_of_Cholesterol_Biosynthesis_by_SREBP_(SREBF)_WP2686_76888	0.05284937	1	16
Hs_Cell_Differentiation_-_Index_WP2029_69036	0.056516185	1	54
Hs_Neural_Crest_Differentiation_WP2064_79263	0.056628454	2	101
Hs_Toll-like_receptor_signaling_pathway_WP75_72133	0.059684988	2	102
Hs_Serotonin_Receptor_2_and_ELK-SRF-GATA4_signaling_WP732_74437	0.06380749	1	17
Hs_GPCR_downstream_signaling_WP1824_76910	0.06430161	4	406
Hs_MyD88_cascade_initiated_on_plasma_membrane_WP2801_77090	0.06743209	1	19
Hs_miR-targeted_genes_in_adipocytes_-_TarBase_WP2001_78529	0.06743209	1	38
Hs_Serotonin_Receptor_4-6-7_and_NR3C_Signaling_WP734_74438	0.071042724	1	19
Hs_Regulation_of_Lipid_Metabolism_by_Peroxisome_proliferator-activated_receptor_alpha_(PPARalpha)_WP2797_77088	0.07135508	2	118
Hs_Spinal_Cord_Injury_WP2431_78556	0.07135508	2	116
Hs_Eicosanoid_Synthesis_WP167_71381	0.07463945	1	25
Hs_Hypertrophy_Model_WP516_71358	0.07463945	1	20
Hs_Double-Strand_Break_Repair_WP1807_76953	0.07822232	1	21
Hs_MyD88_dependent_cascade_initiated_on_endosome_WP2768_77040	0.07822232	1	21
Hs_Signaling_by_BMP_WP2760_77018	0.07822232	1	21
Hs_Blood_Clotting_Cascade_WP272_71361	0.081791386	1	22
Hs_Cell_Differentiation_-_meta_WP2023_68892	0.081791386	1	67
Hs_MAP_kinase_activation_in_TLR_cascade_WP2792_78712	0.081791386	1	22
Hs_Processing_of_Capped_Intronless_Pre-mRNA_WP1890_76858	0.0853467	1	23
Hs_TAK1_activates_NFkB_by_phosphorylation_and_activation_of_IKKs_complex_WP2656_76831	0.0853467	1	24
Hs_miR-targeted_genes_in_leukocytes_-_TarBase_WP2003_78572	0.087162286	2	160
Hs_IL1_and_megakaryotyces_in_obesity_WP2865_78483	0.08888832	1	24
Hs_Physiological_and_Pathological_Hypertrophy_of_the_Heart_WP1528_78581	0.08888832	1	24
Hs_Ectoderm_Commitment_Pathway_WP2856_78535	0.091855206	2	145
Hs_Ectoderm_Differentiation_WP2858_78578	0.091855206	2	145
Hs_TGF_beta_Signaling_Pathway_WP366_79341	0.091855206	2	131
Hs_Differentiation_of_white_and_brown_adipocyte_WP2895_79216	0.09241629	1	25
Hs_YAP1-_and_WWTR1_(TAZ)-stimulated_gene_expression_WP2738_76971	0.09241629	1	25
Hs_Cytokines_and_Inflammatory_Response_WP530_79331	0.095930666	1	30
Hs_Arachidonic_acid_metabolism_WP2650_76814	0.0994315	1	27
Hs_MyD88-Mal_cascade_initiated_on_plasma_membrane_WP2761_77020	0.0994315	1	28
Hs_Nanoparticle-mediated_activation_of_receptor_signaling_WP2643_74251	0.10291885	1	28
Hs_Signaling_by_ERBB2_WP2780_77062	0.10291885	1	36
Hs_BDNF_signaling_pathway_WP2380_79265	0.103895985	2	141
Hs_Formation_of_Fibrin_Clot_(Clotting_Cascade)_WP1818_76965	0.10985328	1	30
Hs_Hair_Follicle_Development-_Organogenesis_(Part_2_of_3)_WP2839_78519	0.10985328	1	31
Hs_Oxidative_Stress_WP408_78546	0.10985328	1	30
Hs_Regulation_of_Actin_Cytoskeleton_WP51_79526	0.111315824	2	149
Hs_Prostaglandin_Synthesis_and_Regulation_WP98_72088	0.11330047	1	31
Hs_Trans-sulfuration_and_one_carbon_metabolism_WP2525_78541	0.11330047	1	31
Hs_miRs_in_Muscle_Cell_Differentiation_WP2012_68959	0.11330047	1	40
Hs_Calcium_Regulation_in_the_Cardiac_Cell_WP536_78588	0.11507653	2	150
Hs_Ovarian_Infertility_Genes_WP34_72115	0.11673438	1	32
Hs_Monoamine_GPCRs_WP58_69046	0.12015505	1	34
Hs_Signaling_by_TGF-beta_Receptor_Complex_WP2742_76980	0.12015505	1	36
Hs_Signaling_of_Hepatocyte_Growth_Factor_Receptor_WP313_71094	0.12015505	1	34
Hs_Myometrial_Relaxation_and_Contraction_Pathways_WP289_78540	0.121415235	2	156
Hs_p38_MAPK_Signaling_Pathway_WP400_72084	0.123562545	1	34
Hs_Nucleotide-binding_domain,_leucine_rich_repeat_containing_receptor_(NLR)_signaling_pathways_WP2763_77025	0.13033819	1	38
Hs_Signaling_by_ERBB4_WP2781_77063	0.13033819	1	44
Hs_ATM_Signaling_Pathway_WP2516_78531	0.1370617	1	41
Hs_G13_Signaling_Pathway_WP524_72112	0.1370617	1	38
Hs_Interferon_gamma_signaling_WP1836_77096	0.1370617	1	43
Hs_Nuclear_Receptors_WP170_71083	0.1370617	1	38
Hs_Eukaryotic_Transcription_Initiation_WP405_73594	0.14705011	1	41
Hs_DNA_Replication_WP466_76196	0.15035397	1	42
Hs_Interferon_alpha-beta_signaling_WP1835_77078	0.15035397	1	45

Hs_tRNA_Aminoacylation_WP1938_76923	0.15035397	1	42
Hs_Hair_Follicle_Development-_Induction_(Part_1_of_3)_WP2804_78710	0.15364507	1	44
Hs_JAK-STAT_WP2593_74127	0.15364507	1	45
Hs_JAK-STAT_WP2594_74128	0.15364507	1	45
Hs_Activation_of_Chaperone_Genes_by_XBP1(S)_WP2667_76852	0.16018927	1	46
Hs_Aryl_Hydrocarbon_Receptor_Pathway_WP2873_79696	0.16018927	1	46
Hs_Aryl_Hydrocarbon_Receptor_pathway_WP2873_79544	0.16018927	1	46
Hs_Focal_Adhesion_WP306_78800	0.16101788	2	188
Hs_Aryl_Hydrocarbon_Receptor_WP2586_78547	0.16344248	1	47
Hs_Focal_Adhesion_WP306_79698	0.16373767	2	188
Hs_Energy_Metabolism_WP1541_68947	0.16668314	1	47
Hs_Transport_of_glucose_and_other_sugars,_bile_salts_and_organic_acids,_metal_ions_and_amine_compounds_WP1935_76949	0.17952134	1	52
Hs_Vitamin_B12_Metabolism_WP1533_70117	0.17952134	1	53
Hs_Wnt_Signaling_Pathway_Netpath_WP363_78571	0.17952134	1	51
Hs_Cardiac_Progenitor_Differentiation_WP2406_73324	0.18270001	1	53
Hs_Apoptosis-related_network_due_to_altered_Notch3_in_ovarian_cancer_WP2864_79278	0.18586643	1	53
Hs_ErbB_Signaling_Pathway_WP673_69914	0.18902065	1	54
Hs_Interferon_type_I_signaling_pathways_WP585_79096	0.18902065	1	54
Hs_Pathogenic_Escherichia_coli_infection_WP2272_78594	0.19529265	1	64
Hs_Activation_of_Gene_Expression_by_SREBP_(SREBF)_WP2706_76917	0.19841054	1	60
Hs_Costimulation_by_the_CD28_family_WP1799_77064	0.19841054	1	71
Hs_Cell_junction_organization_WP1793_77057	0.20769224	1	61
Hs_Metabolism_of_nucleotides_WP1851_76838	0.20769224	1	65
Hs_Cytosolic_sensors_of_pathogen-associated_DNA_WP2794_77085	0.2107623	1	62
Hs_Integrin_cell_surface_interactions_WP1833_77019	0.21382055	1	64
Hs_Leptin_signaling_pathway_WP2034_79545	0.21382055	1	62
Hs_SREBP_signalling_WP1982_78494	0.21382055	1	65
Hs_Semaphorin_interactions_WP1907_76850	0.21382055	1	62
Hs_Angiogenesis_overview_WP1993_71385	0.21990168	1	65
Hs_Folate_Metabolism_WP176_74202	0.21990168	1	67
Hs_TSH_signaling_pathway_WP2032_78548	0.22593601	1	66
Hs_Wnt_Signaling_Pathway_WP428_79528	0.22593601	1	67
Hs_Regulation_of_DNA_replication_WP1898_76824	0.22893575	1	70
Hs_AMPK_Signaling_WP1403_79471	0.23192391	1	68
Hs_Mesodermal_Commitment_Pathway_WP2857_78577	0.23192391	1	154
Hs_PIP3_activates_AKT_signaling_WP2653_76821	0.2378657	1	90
Hs_Primary_Focal_Segmental_Glomerulosclerosis_FSGS_WP2572_79296	0.24376176	1	74
Hs_Arrhythmogenic_Right_Ventricular_Cardiomyopathy_WP2118_71265	0.24961242	1	78
Hs_GPCRs,_Class_A_Rhodopsin-like_WP455_78510	0.25512338	2	262
Hs_GPCRs,_Class_A_Rhodopsin-like_WP455_79717	0.25512338	2	262
Hs_M-G1_Transition_WP2785_77074	0.25541806	1	79
Hs_Prolactin_Signaling_Pathway_WP2037_78501	0.25541806	1	76
Hs_Apoptosis_Modulation_and_Signaling_WP1772_63162	0.26689556	1	93
Hs_Hair_Follicle_Development-_Cytodifferentiation_(Part_3_of_3)_WP2840_78512	0.26689556	1	87
Hs_Integration_of_energy_metabolism_WP1831_77011	0.26689556	1	81
Hs_MicroRNAs_in_cardiomyocyte_hypertrophy_WP1544_75258	0.27819702	1	104
Hs_Allograft_Rejection_WP2328_78554	0.28378257	1	100
Hs_miR-targeted_genes_in_epithelium_-_TarBase_WP2002_78530	0.28800288	2	345
Hs_Androgen_receptor_signaling_pathway_WP138_79277	0.29208037	1	89
Hs_GPCRs,_Other_WP117_71231	0.297559	1	118
Hs_GPCRs,_Other_WP117_79657	0.30028248	1	118
Hs_GPCRs,_Other_WP117_79718	0.30028248	1	118
Hs_G_Protein_Signaling_Pathways_WP35_71252	0.30028248	1	92
Hs_B_Cell_Receptor_Signaling_Pathway_WP23_78566	0.30299544	1	94
Hs_DNA_Damage_Response_(only_ATM_dependent)_WP710_70109	0.30569795	1	97
Hs_Integrin-mediated_Cell_Adhesion_WP185_71391	0.3190549	1	99
Hs_Wnt_Signaling_Pathway_and_Pluripotency_WP399_79474	0.32169545	1	101
Hs_RNA_Polymerase_II_Transcription_WP1906_76887	0.3243258	1	101
Hs_Cell_Cycle_WP179_70629	0.3295561	1	103
Hs_Cell_Cycle_Checkpoints_WP1775_76816	0.3500775	1	115
Hs_Prostate_Cancer_WP2263_73838	0.3500775	1	115
Hs_Mitotic_G1-G1-S_phases_WP1858_76928	0.3675203	1	120
Hs_miR-targeted_genes_in_squamous_cell_-_TarBase_WP2006_78523	0.38210002	1	160
Hs_mRNA_Processing_WP411_71369	0.38449723	1	127
Hs_HIV_Life_Cycle_WP2658_76836	0.38926396	1	143
Hs_Processing_of_Capped_Intron-Containing_Pre-mRNA_WP1889_77003	0.40796635	1	144
Hs_Immunoregulatory_interactions_between_a_Lymphoid_and_a_non-Lymphoid_cell_WP1829_76993	0.4171027	1	292
Hs_NRF2_pathway_WP2884_79518	0.4171027	1	143
Hs_NRF2_pathway_WP2884_79616	0.4171027	1	143
Hs_Integrated_Breast_Cancer_Pathway_WP1984_72732	0.47110987	1	164
Hs_Metapathway_biotransformation_WP702_73516	0.49129134	1	188
Hs_Influenza_Life_Cycle_WP2683_76880	0.5068833	1	217
Hs_Metabolism_of_amino_acids_and_derivatives_WP2693_76898	0.5068833	1	184

Table S2 Pathway analysis through WikiPathways in the fibroblast irradiated with UV-C (upregulation, 4hr, 5J/m^2)

	p-value(high dose 4hr)	Matched Entities (high dose 4hr)	Pathway Entities of Experiment Type (high dose 4hr)
Hs_Integrated_Pancreatic_Cancer_Pathway_WP2377_71228	3.35E-07	13	200
Hs_miRNA_Regulation_of_DNA_Damage_Response_WP1530_79564	1.48E-06	8	98
Hs_miRNA_Regulation_of_DNA_Damage_Response_WP1530_78503	1.48E-06	8	98
Hs_miRNAs_involved_in_DNA_damage_response_WP1545_78559	1.52E-06	5	69
Hs_Activation_of_Gene_Expression_by_SREBP_(SREBF)_WP2706_76917	2.84E-06	7	60
Hs_DNA_Damage_Response_WP707_78527	9.39E-06	7	68
Hs_Glucocorticoid_Receptor_Pathway_WP2880_79522	1.14E-05	7	71
Hs_Glucocorticoid_Receptor_Pathway_WP2880_79615	1.14E-05	7	71
Hs_Differentiation_Pathway_WP2848_78558	1.50E-05	6	50
Hs_Senescence_and_Autophagy_WP615_71375	2.19E-05	8	106
Hs_ATM_Signaling_Pathway_WP2516_78531	5.54E-05	5	41
Hs_Influenza_Life_Cycle_WP2683_76880	1.85E-04	9	217
Hs_ErbB_Signaling_Pathway_WP673_69914	3.03E-04	5	54
Hs_Cholesterol_Biosynthesis_WP197_78758	5.26E-04	3	15
Hs_Endochondral_Ossification_WP474_72122	6.68E-04	5	64
Hs_Nuclear_Receptors_Meta-Pathway_WP2882_78569	7.08E-04	11	318
Hs_Processing_of_Capped_Intron-Containing_Pre-mRNA_WP1889_77003	7.19E-04	7	144
Hs_G1_to_S_cell_cycle_control_WP45_71377	8.81E-04	5	68
Hs_Nonsense-Mediated_Decay_WP2710_76924	0.00103763	6	111
Hs_Cholesterol_biosynthesis_WP1795_77044	0.001084527	3	21
Hs_Double-Strand_Break_Repair_WP1807_76953	0.001464754	3	21
Hs_TP53_Network_WP1742_71700	0.001464754	3	22
Hs_miR-targeted_genes_in_lymphocytes_-_TarBase_WP2004_78524	0.002013569	12	495
Hs_Eukaryotic_Translation_Termination_WP1813_77051	0.002148859	5	88
Hs_Cytoplasmic_Ribosomal_Proteins_WP477_67139	0.002639863	5	88
Hs_RB_in_Cancer_WP2446_78573	0.002639863	5	87
Hs_Regulation_of_Water_Balance_by_Renal_Aquaporins_WP2662_76842	0.002750504	3	26
Hs_DNA_Damage_Response_(only_ATM_dependent)_WP710_70109	0.003687158	5	97
Hs_Meiotic_Recombination_WP2698_76904	0.003773517	3	29
Hs_Hair_Follicle_Development-_Organogenesis_(Part_2_of_3)_WP2839_78519	0.004159304	3	31
Hs_Ovarian_Infertility_Genes_WP34_72115	0.00500053	3	32
Hs_Cell_Cycle_WP179_70629	0.0054332	5	103
Hs_Integrated_Cancer_pathway_WP1971_71249	0.006971653	3	36
Hs_Cell_Cycle_Checkpoints_WP1775_76816	0.007415812	5	115
Hs_Parkin-Ubiquitin_Proteasomal_System_pathway_WP2359_72121	0.007536539	4	73
Hs_S_Phase_WP2772_77049	0.007980794	5	116
Hs_Parkinsons_Disease_Pathway_WP2371_79311	0.010001581	3	62
Hs_Apoptosis_Modulation_and_Signaling_WP1772_63162	0.011370802	4	93
Hs_Selenium_Micronutrient_Network_WP15_78776	0.01186123	4	84
Hs_Heart_Development_WP1591_78590	0.012132745	3	47
Hs_Signaling_Pathways_in_Glioblastoma_WP2261_78522	0.01236502	4	82
Hs_Adipogenesis_WP236_78584	0.014031468	5	131
Hs_Energy_Metabolism_WP1541_68947	0.014504525	3	47
Hs_Eukaryotic_Translation_Elongation_WP1811_77053	0.014516179	4	91
Hs_GPCR_ligand_binding_WP1825_76977	0.015639506	9	371
Hs_Focal_Adhesion_WP306_78800	0.01611156	6	188
Hs_Circadian_Clock_WP1797_76871	0.01622103	3	49
Hs_Androgen_receptor_signaling_pathway_WP138_79277	0.016275452	4	89
Hs_Focal_Adhesion_WP306_79698	0.016897012	6	188
Hs_Small_Ligand_GPCRs_WP247_74422	0.017816916	2	19
Hs_Mitochondrial_Gene_Expression_WP391_71373	0.017816916	2	19
Hs_Vitamin_B12_Metabolism_WP1533_70117	0.018046608	3	53
Hs_Eicosanoid_Synthesis_WP167_71381	0.019655943	2	25
Hs_GPCRs,_Class_A_Rhodopsin-like_WP455_78510	0.02096546	7	262
Hs_GPCRs,_Class_A_Rhodopsin-like_WP455_79717	0.02096546	7	262
Hs_Signaling_by_Type_1_Insulin-like_Growth_Factor_1_Receptor_(IGF1R)_WP2677_76867	0.024181457	3	75
Hs_RNA_Polymerase_II_Transcription_WP1906_76887	0.02461645	4	101
Hs_SRP-dependent_cotranslational_protein_targeting_to_membrane_WP2737_76970	0.027037729	4	111
Hs_IL1_and_megakaryotyces_in_obesity_WP2865_78483	0.027751973	2	24
Hs_Activation_of_Genes_by_ATF4_WP2753_76999	0.029951904	2	25
Hs_SREBP_signalling_WP1982_78494	0.030048158	3	65
Hs_miR-targeted_genes_in_muscle_cell_-_TarBase_WP2005_78538	0.030697625	8	409
Hs_miR-targeted_genes_in_epithelium_-_TarBase_WP2002_78530	0.031627305	7	345
Hs_Cytokines_and_Inflammatory_Response_WP530_79331	0.032218687	2	30
Hs_Eukaryotic_Translation_Initiation_WP1812_76969	0.033223756	4	117
Hs_Prostate_Cancer_WP2263_73838	0.033223756	4	115
Hs_Integrated_Breast_Cancer_Pathway_WP1984_72732	0.033954494	5	164
Hs_Arachidonic_acid_metabolism_WP2650_76814	0.03455062	2	27
Hs_Spinal_Cord_Injury_WP2431_78556	0.035130322	4	116
Hs_Regulation_of_Lipid_Metabolism_by_Peroxisome_proliferator-activated_receptor_alpha_(PPARalpha)_WP2797_77088	0.035130322	4	118
Hs_TSH_signaling_pathway_WP2032_78548	0.035231445	3	66
Hs_AMPK_Signaling_WP1403_79471	0.037984595	3	68
Hs_Mitotic_G1-G1-S_phases_WP1858_76928	0.040168896	4	120
Hs_PIP3_activates_AKT_signaling_WP2653_76821	0.040844254	3	90
Hs_Oxidative_Stress_WP408_78546	0.041920666	2	30
Hs_DNA_Damage_Bypass_WP1803_76966	0.042739253	1	4
Hs_miRs_in_Muscle_Cell_Differentiation_WP2012_68959	0.044496696	2	40
Hs_Prostaglandin_Synthesis_and_Regulation_WP98_72088	0.044496696	2	31
Hs_Bladder_Cancer_WP2828_79529	0.044496696	2	31
Hs_Elastic_fibre_formation_WP2666_76849	0.047129776	2	32
Hs_Inflammatory_Response_Pathway_WP453_63217	0.047129776	2	33
Hs_Assembly_of_collagen_fibrils_and_other_multimeric_structures_WP2798_77089	0.047129776	2	33
Hs_mRNA_Processing_WP411_71369	0.047876894	4	127
Hs_RNA_Polymerase_I,_RNA_Polymerase_III,_and_Mitochondrial_Transcription_WP1905_77034	0.048452437	3	76
Hs_Signaling_of_Hepatocyte_Growth_Factor_Receptor_WP313_71094	0.04981836	2	34
Hs_Prolactin_Signaling_Pathway_WP2037_78501	0.050051574	3	76
Hs_HIV_Life_Cycle_WP2658_76836	0.050219007	4	143
Hs_Mitochondrial_Iron-Sulfur_Cluster_Biogenesis_WP2702_76911	0.053136047	1	7
Hs_Interleukin-3,_5_and_GM-CSF_signaling_WP1840_77073	0.058202166	2	37
Hs_Interleukin-1_signaling_WP1839_76943	0.061097924	2	38
Hs_Lipid_digestion,_mobilization,_and_transport_WP2764_77026	0.061097924	2	41
Hs_Gastrin-CREB_signalling_pathway_via_PKC_and_MAPK_WP2664_76844	0.061520673	4	147
Hs_Metabolism_of_carbohydrates_WP1848_76833	0.06194806	3	87
Hs_GPCR_downstream_signaling_WP1824_76910	0.06237681	8	406
Hs_Interleukin-7_signaling_WP2673_76857	0.06342012	1	6
Hs_Apoptosis_WP254_78808	0.06374565	3	84
Hs_Nuclear_Receptors_WP170_71083	0.06404189	2	38
Hs_BDNF_signaling_pathway_WP2380_79265	0.06833124	4	141
Hs_Eukaryotic_Transcription_Initiation_WP405_73594	0.0731493	2	41
Hs_IL-2_Signaling_Pathway_WP49_78543	0.07627249	2	42
Hs_Synthesis_of_DNA_WP1925_76968	0.07698641	3	94
Hs_JAK-STAT_WP2593_74127	0.0794372	2	45
Hs_IL-6_signaling_pathway_WP364_78561	0.0794372	2	43
Hs_JAK-STAT_WP2594_74128	0.0794372	2	45
Hs_Hair_Follicle_Development-_Induction_(Part_1_of_3)_WP2804_78710	0.0794372	2	44
Hs_Interleukin-11_Signaling_Pathway_WP2332_79525	0.08264218	2	44
Hs_Non-homologous_end_joining_WP438_68977	0.08365496	1	8
Hs_Uptake_of_Carbon_Dioxide_and_Release_of_Oxygen_by_Erythrocytes_WP2744_76982	0.08365496	1	8
Hs_Uptake_of_Oxygen_and_Release_of_Carbon_Dioxide_by_Erythrocytes_WP2681_76878	0.08365496	1	8
Hs_TFs_Regulate_miRNAs_related_to_cardiac_hypertrophy_WP1559_68890	0.08365496	1	15

Hs_Activation_of_Chaperone_Genes_by_XBP1(S)_WP2667_76852	0.08588617	2	46
Hs_Aryl_Hydrocarbon_Receptor_WP2586_78547	0.08916795	2	47
Hs_Metabolism_of_non-coding_RNA_WP2715_76932	0.08916795	2	55
Hs_Neural_Crest_Differentiation_WP2064_79263	0.09345506	3	101
Hs_Diurnally_Regulated_Genes_with_Circadian_Orthologs_WP410_69903	0.09584011	2	48
Hs_EGF-EGFR_Signaling_Pathway_WP437_79266	0.10089865	4	162
Hs_Translation_Factors_WP107_78489	0.10264933	2	50
Hs_Complement_and_Coagulation_Cascades_WP558_67786	0.106102504	2	64
Hs_Collagen_biosynthesis_and_modifying_enzymes_WP2725_76944	0.106102504	2	51
Hs_Cardiac_Progenitor_Differentiation_WP2406_73324	0.10958658	2	53
Hs_MAPK_Signaling_Pathway_WP382_72103	0.11131226	4	168
Hs_Apoptosis-related_network_due_to_altered_Notch3_in_ovarian_cancer_WP2864_79278	0.1131005	2	53
Hs_Nucleotide_GPCRs_WP80_68938	0.113191895	1	11
Hs_Vitamin_D_Metabolism_WP1531_74057	0.113191895	1	11
Hs_Iron_metabolism_in_placenta_WP2007_69751	0.113191895	1	12
Hs_TGF_Beta_Signaling_Pathway_WP560_68944	0.11664319	2	55
Hs_IL-1_signaling_pathway_WP195_78528	0.12021363	2	55
Hs_Synthesis,_Secretion,_and_Deacylation_of_Ghrelin_WP1926_76897	0.1228248	1	15
Hs_BMP_Signalling_and_Regulation_WP1425_74390	0.1228248	1	12
Hs_Peptide_hormone_biosynthesis_WP2691_76894	0.1228248	1	12
Hs_Pathogenic_Escherichia_coli_infection_WP2272_78594	0.12381079	2	64
Hs_Extracellular_matrix_organization_WP2703_76914	0.1310813	2	58
Hs_Homologous_recombination_WP186_68935	0.13235326	1	13
Hs_Dopamine_metabolism_WP2436_71387	0.13235326	1	13
Hs_Regulation_of_Insulin-like_Growth_Factor_(IGF)_Transport_and_Uptake_by_Insulin-like_Growth_Factor_Binding_Proteins_(IGFBPs)_WP2799_77094	0.13235326	1	13
Hs_SRF_and_miRs_in_Smooth_Muscle_Differentiation_and_Proliferation_WP1991_75261	0.13235326	1	17
Hs_Platelet_Adhesion_to_exposed_collagen_WP1883_76862	0.13235326	1	13
Hs_Signaling_by_Activin_WP2791_77082	0.13235326	1	13
Hs_Kit_receptor_signaling_pathway_WP304_78799	0.13475272	2	59
Hs_Host_Interactions_of_HIV_factors_WP2684_76883	0.1375589	3	137
Hs_Complement_and_Coagulation_Cascades_WP558_79680	0.13844697	2	61
Hs_Metabolism_of_nucleotides_WP1851_76838	0.13844697	2	65
Hs_Quercetin_and_Nf-kB-_AP-1_Induced_Cell_Apoptosis_WP2435_79692	0.14177838	1	15
Hs_Quercetin_and_Nf-kB-_AP-1_Induced_Cell_Apoptosis_WP2435_79583	0.14177838	1	15
Hs_Regulation_of_Cholesterol_Biosynthesis_by_SREBP_(SREBF)_WP2686_76888	0.14177838	1	16
Hs_Cytosolic_sensors_of_pathogen-associated_DNA_WP2794_77085	0.14216313	2	62
Hs_TarBasePathway_WP1992_78296	0.1511013	1	18
Hs_Cell_Differentiation_-_Index_WP2029_69036	0.1511013	1	54
Hs_miR-targeted_genes_in_squamous_cell_-_TarBase_WP2006_78523	0.15272947	3	160
Hs_Proteasome_Degradation_WP183_71712	0.15343408	2	65
Hs_Folate_Metabolism_WP176_74202	0.15343408	2	67
Hs_Oncostatin_M_Signaling_Pathway_WP2374_73668	0.15722898	2	65
Hs_SREBF_and_miR33_in_cholesterol_and_lipid_homeostasis_WP2011_75253	0.16032314	1	18
Hs_Osteoclast_Signaling_WP12_78593	0.16032314	1	16
Hs_Sulfation_Biotransformation_Reaction_WP692_69031	0.16032314	1	18
Hs_ID_signaling_pathway_WP53_67360	0.16032314	1	16
Hs_miR-targeted_genes_in_leukocytes_-_TarBase_WP2003_78572	0.16050488	3	160
Hs_Regulation_of_DNA_replication_WP1898_76824	0.16487053	2	70
Hs_Mesodermal_Commitment_Pathway_WP2857_78577	0.16871555	2	154
Hs_Endoderm_Differentiation_WP2853_78496	0.16871555	2	146
Hs_Kinesins_WP1842_76861	0.16944496	1	18
Hs_ACE_Inhibitor_Pathway_WP554_77712	0.16944496	1	17
Hs_TGF_beta_Signaling_Pathway_WP366_79341	0.17105316	3	131
Hs_Ectoderm_Differentiation_WP2858_78578	0.17105316	3	145
Hs_Ectoderm_Commitment_Pathway_WP2856_78535	0.17105316	3	145
Hs_miR-targeted_genes_in_adipocytes_-_TarBase_WP2001_78529	0.17846787	1	38
Hs_MyD88_cascade_initiated_on_plasma_membrane_WP2801_77090	0.17846787	1	19
Hs_Polycystic_Kidney_Disease_Pathway_WP2571_78508	0.17846787	1	18
Hs_Primary_Focal_Segmental_Glomerulosclerosis_FSGS_WP2572_79296	0.18423897	2	74
Hs_Peptide_GPCRs_WP24_79444	0.18423897	2	73
Hs_Serotonin_Receptor_4-6-7_and_NR3C_Signaling_WP734_74438	0.18739292	1	19
Hs_Nucleotide_Metabolism_WP404_68960	0.18739292	1	19
Hs_Base_Excision_Repair_WP1787_78301	0.18739292	1	19
Hs_Transcriptional_Regulation_of_White_Adipocyte_Differentiation_WP2751_76992	0.18815187	2	74
Hs_NRF2_pathway_WP2884_79518	0.19270755	3	143
Hs_NRF2_pathway_WP2884_79616	0.19270755	3	143
Hs_Hypertrophy_Model_WP516_71358	0.19622119	1	20
Hs_PDGF_Pathway_WP2526_78551	0.19622119	1	37
Hs_MyD88_dependent_cascade_initiated_on_endosome_WP2768_77040	0.2049537	1	21
Hs_Signaling_by_BMP_WP2760_77018	0.2049537	1	21
Hs_Cell_Differentiation_-_meta_WP2023_68892	0.21359152	1	67
Hs_MAP_kinase_activation_in_TLR_cascade_WP2792_78712	0.21359152	1	22
Hs_Detoxification_of_Reactive_Oxygen_Species_WP2824_76144	0.21359152	1	26
Hs_Regulation_of_Actin_Cytoskeleton_WP51_79526	0.21499619	3	149
Hs_Hair_Follicle_Development-_Cytodifferentiation_(Part_3_of_3)_WP2840_78512	0.21582143	2	87
Hs_Regulation_of_mRNA_Stability_by_Proteins_that_Bind_AU-rich_Elements_WP2733_76960	0.21582143	2	83
Hs_Mitotic_Metaphase_and_Anaphase_WP2757_77009	0.22065242	3	153
Hs_TAK1_activates_NFkB_by_phosphorylation_and_activation_of_IKKs_complex_WP2656_76831	0.22213565	1	24
Hs_Transport_of_vitamins,_nucleosides,_and_related_molecules_WP1937_77048	0.22213565	1	24
Hs_Metabolism_of_steroid_hormones_and_vitamin_D_WP2749_76990	0.22213565	1	23
Hs_Processing_of_Capped_Intronless_Pre-mRNA_WP1890_76858	0.22213565	1	23
Hs_Calcium_Regulation_in_the_Cardiac_Cell_WP536_78588	0.2234918	3	150
Hs_Signal_Transduction_of_S1P_Receptor_WP26_78492	0.23058711	1	25
Hs_Physiological_and_Pathological_Hypertrophy_of_the_Heart_WP1528_78581	0.23058711	1	24
Hs_MicroRNAs_in_cardiomyocyte_hypertrophy_WP1544_75258	0.23179436	2	104
Hs_Myometrial_Relaxation_and_Contraction_Pathways_WP289_78540	0.23779191	3	156
Hs_YAP1-_and_WWTR1_(TAZ)-stimulated_gene_expression_WP2738_76971	0.23894691	1	25
Hs_Post-translational_modification-_synthesis_of_GPI-anchored_proteins_WP1887_77093	0.23894691	1	26
Hs_Differentiation_of_white_and_brown_adipocyte_WP2895_79216	0.23894691	1	25
Hs_Phase_II_conjugation_WP1880_76996	0.23894691	1	26
Hs_Regulatory_RNA_pathways_WP1901_76851	0.24721603	1	26
Hs_MyD88-Mal_cascade_initiated_on_plasma_membrane_WP2761_77020	0.25539547	1	28
Hs_Extracellular_vesicle-mediated_signaling_in_recipient_cells_WP2870_78078	0.25539547	1	30
Hs_FSH_signaling_pathway_WP2035_78536	0.25539547	1	27
Hs_Arylhydrocarbon_receptor_(AhR)_signaling_pathway_WP2100_74081	0.25539547	1	28
Hs_Signaling_by_ERBB2_WP2780_77062	0.2634862	1	36
Hs_Nanoparticle-mediated_activation_of_receptor_signaling_WP2643_74251	0.2634862	1	28
Hs_mRNA_Capping_WP1861_76815	0.2634862	1	28
Hs_Interleukin-2_signaling_WP2732_76959	0.2634862	1	29
Hs_Statin_Pathway_WP430_78268	0.27148917	1	31
Hs_Extracellular_vesicle-mediated_signaling_in_recipient_cells_WP2870_79555	0.27148917	1	30
Hs_Mitotic_Prometaphase_WP2652_76819	0.27597335	2	98
Hs_DAG_and_IP3_signaling_WP2688_76890	0.27940533	1	30
Hs_Dopaminergic_Neurogenesis_WP2855_79211	0.27940533	1	30
Hs_Formation_of_Fibrin_Clot_(Clotting_Cascade)_WP1818_76965	0.27940533	1	30
Hs_Gastric_cancer_network_2_WP2363_76329	0.28723562	1	32
Hs_Integrin-mediated_Cell_Adhesion_WP185_71391	0.29203394	2	99
Hs_Mitochondrial_Protein_Import_WP2717_76934	0.29498094	1	32
Hs_Wnt_Signaling_Pathway_and_Pluripotency_WP399_79474	0.29604265	2	101
Hs_Nuclear_Receptors_in_Lipid_Metabolism_and_Toxicity_WP299_78587	0.3026423	1	35
Hs_Meiotic_Synapsis_WP2731_76957	0.3026423	1	35
Hs_Fluoropyrimidine_Activity_WP1601_78516	0.3026423	1	33
Hs_Signaling_by_TGF-beta_Receptor_Complex_WP2742_76980	0.3026423	1	36

Hs_Protein_folding_WP1892_76909	0.3026423	1	33
Hs_TOR_Signaling_WP1471_70031	0.3102205	1	34
Hs_p38_MAPK_Signaling_Pathway_WP400_72084	0.3102205	1	34
Hs_Metabolism_of_amino_acids_and_derivatives_WP2693_76898	0.31692162	3	184
Hs_Hexose_transport_WP1828_77015	0.3177165	1	39
Hs_Intrinsic_Pathway_for_Apoptosis_WP1841_76964	0.3177165	1	35
Hs_Telomere_Maintenance_WP1928_76893	0.3251312	1	37
Hs_Nucleotide-binding_domain,_leucine_rich_repeat_containing_receptor_(NLR)_signaling_pathways_WP2763_77025	0.3251312	1	38
Hs_Signaling_by_ERBB4_WP2781_77063	0.3251312	1	44
Hs_Glycogen_Metabolism_WP500_63201	0.3251312	1	36
Hs_Interferon_gamma_signaling_WP1836_77096	0.33972016	1	43
Hs_G13_Signaling_Pathway_WP524_72112	0.33972016	1	38
Hs_Mitotic_Prophase_WP2654_76823	0.35399428	1	44
Hs_IL-5_Signaling_Pathway_WP127_78498	0.35399428	1	40
Hs_TWEAK_Signaling_Pathway_WP2036_78525	0.3610154	1	41
Hs_Integrated_Lung_Cancer_Pathway_WP2512_71235	0.3610154	1	44
Hs_Transcriptional_activity_of_SMAD2-SMAD3-SMAD4_heterotrimer_WP2755_77005	0.36796036	1	44
Hs_Interferon_alpha-beta_signaling_WP1835_77078	0.36796036	1	45
Hs_tRNA_Aminoacylation_WP1938_76923	0.36796036	1	42
Hs_Neurotransmitter_Receptor_Binding_And_Downstream_Transmission_In_The_Postsynaptic_Cell_WP2754_77001	0.3711472	2	124
Hs_Regulation_of_Microtubule_Cytoskeleton_WP2038_78570	0.37482995	1	44
Hs_Aryl_Hydrocarbon_Receptor_pathway_WP2873_79544	0.38834637	1	46
Hs_Aryl_Hydrocarbon_Receptor_Pathway_WP2873_79696	0.38834637	1	46
Hs_Selenium_Metabolism_and_Selenoproteins_WP28_71888	0.39499477	1	48
Hs_Tryptophan_metabolism_WP465_77387	0.39499477	1	80
Hs_Tryptophan_metabolism_WP465_79226	0.39499477	1	80
Hs_Structural_Pathway_of_Interleukin_1_(IL-1)_WP2637_79580	0.40157104	1	49
Hs_Structural_Pathway_of_Interleukin_1_(IL-1)_WP2637_76330	0.40157104	1	49
Hs_Signalling_by_NGF_WP1976_76994	0.40157104	1	50
Hs_Signaling_by_FGFR_WP1911_76830	0.40807596	1	69
Hs_Deadenylation-dependent_mRNA_decay_WP2659_76837	0.41451028	1	49
Hs_Nucleotide_Excision_Repair_WP1980_76859	0.41451028	1	49
Hs_Transport_of_glucose_and_other_sugars,_bile_salts_and_organic_acids,_metal_ions_and_amine_compounds_WP1935_76949	0.42717025	1	52
Hs_Wnt_Signaling_Pathway_Netpath_WP363_78571	0.42717025	1	51
Hs_ISG15_antiviral_mechanism_WP2672_76856	0.43339738	1	61
Hs_Regulation_of_Apoptosis_WP1896_77071	0.43339738	1	53
Hs_Degradation_of_beta-catenin_by_the_destruction_complex_WP2773_77050	0.43955693	1	55
Hs_Asparagine_N-linked_glycosylation_WP1785_78300	0.43955693	1	53
Hs_Interferon_type_I_signaling_pathways_WP585_79096	0.44564962	1	54
Hs_Cardiac_Hypertrophic_Response_WP2795_78544	0.44564962	1	54
Hs_IL-4_Signaling_Pathway_WP395_78576	0.44564962	1	55
Hs_Regulation_of_toll-like_receptor_signaling_pathway_WP1449_77378	0.4573962	2	150
Hs_Opioid_Signalling_WP1978_76919	0.45763743	1	56
Hs_Regulation_of_toll-like_receptor_signaling_pathway_WP1449_79550	0.4609936	2	150
Hs_Costimulation_by_the_CD28_family_WP1799_77064	0.4635339	1	71
Hs_Cell_junction_organization_WP1793_77057	0.4808422	1	61
Hs_Notch_Signaling_Pathway_WP61_78592	0.48648685	1	61
Hs_Metabolism_of_water-soluble_vitamins_and_cofactors_WP1857_76875	0.49207026	1	67
Hs_Semaphorin_interactions_WP1907_76850	0.49207026	1	62
Hs_Binding_and_Uptake_of_Ligands_by_Scavenger_Receptors_WP2784_77068	0.49207026	1	195
Hs_Leptin_signaling_pathway_WP2034_79545	0.49207026	1	62
Hs_Integrin_cell_surface_interactions_WP1833_77019	0.49207026	1	64
Hs_Histone_Modifications_WP2369_69927	0.5030559	1	67
Hs_Insulin_Signaling_WP481_72080	0.5232546	2	161
Hs_SIDS_Susceptibility_Pathways_WP706_78533	0.5298689	2	166
Hs_Factors_involved_in_megakaryocyte_development_and_platelet_production_WP1815_77027	0.53461	1	71
Hs_L1CAM_interactions_WP1843_76876	0.539671	1	75
Hs_Signaling_by_Insulin_receptor_WP1913_77046	0.5496288	1	75
Hs_Arrhythmogenic_Right_Ventricular_Cardiomyopathy_WP2118_71265	0.5545268	1	78
Hs_M-G1_Transition_WP2785_77074	0.5641638	1	79
Hs_Metapathway_biotransformation_WP702_73516	0.5651178	2	188
Hs_APC-C-mediated_degradation_of_cell_cycle_proteins_WP1782_77060	0.568904	1	80
Hs_Integration_of_energy_metabolism_WP1831_77011	0.582818	1	81
Hs_MHC_class_II_antigen_presentation_WP2679_76872	0.6006751	1	90
Hs_Alzheimers_Disease_WP2059_79302	0.6093154	1	146
Hs_Allograft_Rejection_WP2328_78554	0.6093154	1	100
Hs_Mitotic_G2-G2-M_phases_WP1859_77022	0.6135653	1	89
Hs_Cell_surface_interactions_at_the_vascular_wall_WP1794_77039	0.6177691	1	91
Hs_TNF_alpha_Signaling_Pathway_WP231_79280	0.62192726	1	90
Hs_Corticotropin-releasing_hormone_WP2355_78490	0.6260402	1	90
Hs_GPCRs,_Other_WP117_71231	0.63010854	1	118
Hs_Corticotropin-releasing_hormone_WP2355_79562	0.63010854	1	91
Hs_GPCRs,_Other_WP117_79718	0.6341327	1	118
Hs_G_Protein_Signaling_Pathways_WP35_71252	0.6341327	1	92
Hs_GPCRs,_Other_WP117_79657	0.6341327	1	118
Hs_B_Cell_Receptor_Signaling_Pathway_WP23_78566	0.6381131	1	94
Hs_Toll-like_receptor_signaling_pathway_WP75_72133	0.6720468	1	102
Hs_Signaling_by_the_B_Cell_Receptor_(BCR)_WP2746_76984	0.7306807	1	239
Hs_Class_I_MHC_mediated_antigen_processing_&_presentation_WP2796_77098	0.76640326	1	148
Hs_Immunoregulatory_interactions_between_a_Lymphoid_and_a_non-Lymphoid_cell_WP1829_76993	0.781255	1	292

Table S3 Pathway analysis through WikiPathways in the fibroblast irradiated with UV-C (upregulation, 12hr, 0.5J/m^2)	p-value (low dose 12hr)	Matched Entities (low dose 12hr)	Pathway Entities of Experiment Type (low dose 12hr)
Hs_Mitotic_Metaphase_and_Anaphase_WP2757_77009	0	27	153
Hs_APC-C-mediated_degradation_of_cell_cycle_proteins_WP1782_77060	0	11	80
Hs_Cell_Cycle_WP179_70629	0	13	103
Hs_Kinesins_WP1842_76861	0	8	18
Hs_Mitotic_Prometaphase_WP2652_76819	0	29	98
Hs_Nucleosome_assembly_WP1874_76826	6.56E-09	6	22
Hs_MHC_class_II_antigen_presentation_WP2679_76872	6.67E-09	9	90
Hs_RB_in_Cancer_WP2446_78573	9.20E-09	9	87
Hs_Gastric_Cancer_Network_1_WP2361_79693	3.20E-08	6	29
Hs_Gastric_cancer_network_1_WP2361_77608	3.20E-08	6	28
Hs_Cell_Cycle_Checkpoints_WP1775_76816	7.85E-08	9	115
Hs_Mitotic_G2-G2-M_phases_WP1859_77022	2.36E-06	7	89
Hs_Mitotic_G1-G1-S_phases_WP1858_76928	1.78E-05	7	120
Hs_DNA_Damage_Response_WP707_78527	1.05E-04	5	68
Hs_ATM_Signaling_Pathway_WP2516_78531	1.30E-04	4	41
Hs_miRNA_Regulation_of_DNA_Damage_Response_WP1530_78503	1.57E-04	5	98
Hs_miRNA_Regulation_of_DNA_Damage_Response_WP1530_79564	1.57E-04	5	98
Hs_Mitotic_Prophase_WP2654_76823	1.60E-04	4	44
Hs_Integrated_Pancreatic_Cancer_Pathway_WP2377_71228	4.65E-04	7	200
Hs_Constitutive_Androstane_Receptor_Pathway_WP2875_79600	0.001225232	3	32
Hs_Gastric_cancer_network_2_WP2363_76329	0.001225232	3	32
Hs_Constitutive_Androstane_Receptor_Pathway_WP2875_79540	0.001225232	3	32
Hs_G1_to_S_cell_cycle_control_WP45_71377	0.00122598	4	68
Hs_Pregnane_X_Receptor_pathway_WP2876_79556	0.00134519	3	33
Hs_Pregnane_X_Receptor_pathway_WP2876_79537	0.00134519	3	33
Hs_Benzo(a)pyrene_metabolism_WP696_72081	0.001616056	2	9
Hs_miR-targeted_genes_in_lymphocytes_-_TarBase_WP2004_78524	0.002229267	9	495
Hs_tRNA_Aminoacylation_WP1938_76923	0.002960857	3	42
Hs_Regulation_of_Microtubule_Cytoskeleton_WP2038_78570	0.003166995	3	44
Hs_Mitotic_Telophase-Cytokinesis_WP2765_77030	0.003994016	2	14
Hs_Diurnally_Regulated_Genes_with_Circadian_Orthologs_WP410_69903	0.004328739	3	48
Hs_Circadian_Clock_WP1797_76871	0.004588024	3	49
Hs_Integrated_Breast_Cancer_Pathway_WP1984_72732	0.005505598	5	164
Hs_Apoptosis-related_network_due_to_altered_Notch3_in_ovarian_cancer_WP2864_79278	0.005718097	3	53
Hs_Nuclear_Receptors_Meta-Pathway_WP2882_78569	0.005988206	7	318
Hs_ErbB_Signaling_Pathway_WP673_69914	0.006024277	3	54
Hs_Double-Strand_Break_Repair_WP1807_76953	0.008931704	2	21
Hs_AMPK_Signaling_WP1403_79471	0.011351611	3	68
Hs_Physiological_and_Pathological_Hypertrophy_of_the_Heart_WP1528_78581	0.011582153	2	24
Hs_FSH_signaling_pathway_WP2035_78536	0.014533243	2	27
Hs_Androgen_receptor_signaling_pathway_WP138_79277	0.023182191	3	89
Hs_Interleukin-1_processing_WP1838_77045	0.02698915	1	4
Hs_BMI1_WP2612_73362	0.02698915	1	4
Hs_G13_Signaling_Pathway_WP524_72112	0.027716557	2	38
Hs_FAS_pathway_and_Stress_induction_of_HSP_regulation_WP314_71366	0.027716557	2	38
Hs_MAPK_Signaling_Pathway_WP382_72103	0.02846594	4	168
Hs_IL-5_Signaling_Pathway_WP127_78498	0.03048075	2	40
Hs_Wnt_Signaling_Pathway_and_Pluripotency_WP399_79474	0.031268287	3	101
Hs_IL-2_Signaling_Pathway_WP49_78543	0.033349227	2	42
Hs_Diclofenac_Metabolic_Pathway_WP2491_73891	0.033622164	1	5
Hs_IL-6_signaling_pathway_WP364_78561	0.034821533	2	43
Hs_Interleukin-11_Signaling_Pathway_WP2332_79525	0.036318664	2	44
Hs_Interleukin-7_signaling_WP2673_76857	0.04021009	1	6
Hs_Nicotine_Metabolism_WP1600_70113	0.04021009	1	6
Hs_Energy_Metabolism_WP1541_68947	0.04095502	2	47
Hs_miR-targeted_genes_in_epithelium_-_TarBase_WP2002_78530	0.041025914	5	345
Hs_Ubiquinol_biosynthesis_WP2734_76961	0.04675323	1	7
Hs_Vitamin_D_Receptor_Pathway_WP2877_79534	0.04675323	1	7
Hs_ISG15_antiviral_mechanism_WP2672_76856	0.04914192	2	61
Hs_IL-4_Signaling_Pathway_WP395_78576	0.05256868	2	55
Hs_Non-homologous_end_joining_WP438_68977	0.05325189	1	8
Hs_TFs_Regulate_miRNAs_related_to_cardiac_hypertrophy_WP1559_68890	0.05325189	1	15
Hs_Adipogenesis_WP236_78584	0.05975023	3	131
Hs_TGF_beta_Signaling_Pathway_WP366_79341	0.060854416	3	131
Hs_Kit_receptor_signaling_pathway_WP304_78799	0.06149002	2	59
Hs_RAF-MAP_kinase_cascade_WP2735_76963	0.06611697	1	10
Hs_Signaling_by_VEGF_WP1919_76864	0.06611697	1	10
Hs_Liver_X_Receptor_Pathway_WP2874_79542	0.06611697	1	10
Hs_Liver_X_Receptor_Pathway_WP2874_79599	0.06611697	1	10
Hs_Leptin_signaling_pathway_WP2034_79545	0.06707184	2	62
Hs_Glucocorticoid_Receptor_Pathway_WP2880_79615	0.082718134	2	71
Hs_Glucocorticoid_Receptor_Pathway_WP2880_79522	0.082718134	2	71
Hs_Insulin_Signaling_WP481_72080	0.098165356	3	161
Hs_EGF-EGFR_Signaling_Pathway_WP437_79266	0.099539064	3	162
Hs_Signaling_by_NODAL_WP2675_76863	0.1036756	1	17
Hs_Signaling_Pathways_in_Glioblastoma_WP2261_78522	0.10798016	2	82
Hs_Apoptosis_WP254_78808	0.11237009	2	84
Hs_Metapathway_biotransformation_WP702_73516	0.11660925	3	188
Hs_Fc_epsilon_receptor_(FCERI)_signaling_WP2759_77017	0.1168056	2	218
Hs_miRNAs_involved_in_DNA_damage_response_WP1545_78559	0.12188618	1	69
Hs_Mitochondrial_Gene_Expression_WP391_71373	0.12188618	1	19
Hs_Focal_Adhesion_WP306_78800	0.13314149	3	188
Hs_Focal_Adhesion_WP306_79698	0.13623118	3	188
Hs_MAP_kinase_activation_in_TLR_cascade_WP2792_78712	0.1397278	1	22
Hs_Processing_of_Capped_Intronless_Pre-mRNA_WP1890_76858	0.14559431	1	23
Hs_Integrin-mediated_Cell_Adhesion_WP185_71391	0.14661095	2	99
Hs_Signal_Transduction_of_S1P_Receptor_WP26_78492	0.15142092	1	25
Hs_Regulation_of_beta-cell_development_WP1897_77067	0.15142092	1	28
Hs_YAP1-_and_WWTR1_(TAZ)-stimulated_gene_expression_WP2738_76971	0.15720792	1	25
Hs_Activation_of_Genes_by_ATF4_WP2753_76999	0.15720792	1	25
Hs_IL-7_Signaling_Pathway_WP205_79472	0.15720792	1	25
Hs_PPAR_Alpha_Pathway_WP2878_79689	0.16295555	1	26
Hs_PPAR_alpha_pathway_WP2878_79532	0.16295555	1	26
Hs_Cytokines_and_Inflammatory_Response_WP530_79331	0.16295555	1	30
Hs_EPO_Receptor_Signaling_WP581_67199	0.16295555	1	26
Hs_Regulatory_RNA_pathways_WP1901_76851	0.16295555	1	26
Hs_Extracellular_vesicle-mediated_signaling_in_recipient_cells_WP2870_78078	0.16866411	1	30
Hs_Prostate_Cancer_WP2263_73838	0.17531618	2	115
Hs_MAPK_Cascade_WP422_72129	0.179965	1	29
Hs_Meiotic_Recombination_WP2698_76904	0.179965	1	29
Hs_Extracellular_vesicle-mediated_signaling_in_recipient_cells_WP2870_79555	0.179965	1	30
Hs_Oxidative_Stress_WP408_78546	0.18555787	1	30
Hs_Trans-sulfuration_and_one_carbon_metabolism_WP2525_78541	0.19111271	1	31
Hs_Host_Interactions_of_HIV_factors_WP2684_76883	0.19244397	2	137
Hs_Elastic_fibre_formation_WP2666_76849	0.19662976	1	32
Hs_Monoamine_Transport_WP727_68928	0.19662976	1	32
Hs_Nuclear_Receptors_in_Lipid_Metabolism_and_Toxicity_WP299_78587	0.20210929	1	35

Hs_Meiotic_Synapsis_WP2731_76957	0.20210929	1	35
Hs_Pre-NOTCH_Expression_and_Processing_WP2786_77076	0.20755155	1	38
Hs_p38_MAPK_Signaling_Pathway_WP400_72084	0.20755155	1	34
Hs_TOR_Signaling_WP1471_70031	0.20755155	1	34
Hs_Hexose_transport_WP1828_77015	0.2129568	1	39
Hs_HIV_Life_Cycle_WP2658_76836	0.21475843	2	143
Hs_Integrated_Cancer_pathway_WP1971_71249	0.21832527	1	36
Hs_Nucleotide-binding_domain,_leucine_rich_repeat_containing_receptor_(NLR)_signaling_pathways_WP2763_77025	0.21832527	1	38
Hs_Glycogen_Metabolism_WP500_63201	0.21832527	1	36
Hs_Ectoderm_Commitment_Pathway_WP2856_78535	0.22475336	2	145
Hs_Ectoderm_Differentiation_WP2858_78578	0.22475336	2	145
Hs_Amyotrophic_lateral_sclerosis_(ALS)_WP2447_75221	0.22895293	1	38
Hs_Interferon_gamma_signaling_WP1836_77096	0.22895293	1	43
Hs_Nuclear_Receptors_WP170_71083	0.22895293	1	38
Hs_Striated_Muscle_Contraction_WP383_68970	0.22895293	1	38
Hs_Vitamin_A_and_Carotenoid_Metabolism_WP716_74442	0.2342126	1	43
Hs_Processing_of_Capped_Intron-Containing_Pre-mRNA_WP1889_77003	0.23478216	2	144
Hs_NOD_pathway_WP1433_68991	0.23943649	1	41
Hs_Glycosaminoglycan_metabolism_WP2743_76981	0.24462485	1	41
Hs_DNA_Replication_WP466_76196	0.24977791	1	42
Hs_BDNF_signaling_pathway_WP2380_79265	0.24986914	2	141
Hs_JAK-STAT_WP2593_74127	0.25489593	1	45
Hs_JAK-STAT_WP2594_74128	0.25489593	1	45
Hs_Apoptotic_execution_phase_WP1784_76813	0.25997913	1	46
Hs_Heart_Development_WP1591_78590	0.25997913	1	47
Hs_Aryl_Hydrocarbon_Receptor_WP2586_78547	0.270042	1	47
Hs_Metabolism_of_non-coding_RNA_WP2715_76932	0.270042	1	55
Hs_Muscle_contraction_WP1864_76895	0.27502215	1	49
Hs_Deadenylation-dependent_mRNA_decay_WP2659_76837	0.28488103	1	49
Hs_Myometrial_Relaxation_and_Contraction_Pathways_WP289_78540	0.28513223	2	156
Hs_Wnt_Signaling_Pathway_Netpath_WP363_78571	0.29460624	1	51
Hs_Regulation_of_Apoptosis_WP1896_77071	0.29941925	1	53
Hs_SIDS_Susceptibility_Pathways_WP706_78533	0.3052255	2	166
Hs_Interferon_type_I_signaling_pathways_WP585_79096	0.3089473	1	54
Hs_Signaling_by_Type_1_Insulin-like_Growth_Factor_1_Receptor_(IGF1R)_WP2677_76867	0.32299757	1	75
Hs_Costimulation_by_the_CD28_family_WP1799_77064	0.32299757	1	71
Hs_Activation_of_Gene_Expression_by_SREBP_(SREBF)_WP2706_76917	0.32299757	1	60
Hs_Extracellular_matrix_organization_WP2703_76914	0.32761744	1	58
Hs_Complement_cascade_WP1798_77042	0.33220583	1	192
Hs_Oxidation_by_Cytochrome_P450_WP43_73490	0.336763	1	63
Hs_Oxidation_by_Cytochrome_P450_WP43_79585	0.34128916	1	63
Hs_Notch_Signaling_Pathway_WP61_78592	0.34128916	1	61
Hs_Semaphorin_interactions_WP1907_76850	0.34578454	1	62
Hs_Binding_and_Uptake_of_Ligands_by_Scavenger_Receptors_WP2784_77068	0.34578454	1	195
Hs_TCR_signaling_WP1927_76950	0.35024932	1	74
Hs_Influenza_Life_Cycle_WP2683_76880	0.35243627	2	217
Hs_Proteasome_Degradation_WP183_71712	0.3546837	1	65
Hs_Endochondral_Ossification_WP474_72122	0.3546837	1	64
Hs_Visual_phototransduction_WP2776_77056	0.3590879	1	67
Hs_Oncostatin_M_Signaling_Pathway_WP2374_73668	0.3590879	1	65
Hs_TSH_signaling_pathway_WP2032_78548	0.36346215	1	66
Hs_Wnt_Signaling_Pathway_WP428_79528	0.36346215	1	67
Hs_AGE-RAGE_pathway_WP2324_78487	0.36346215	1	66
Hs_Endoderm_Differentiation_WP2853_78496	0.3721215	1	146
Hs_PIP3_activates_AKT_signaling_WP2653_76821	0.3806634	1	90
Hs_L1CAM_interactions_WP1843_76876	0.3848908	1	75
Hs_Parkin-Ubiquitin_Proteasomal_System_pathway_WP2359_72121	0.3848908	1	73
Hs_Primary_Focal_Segmental_Glomerulosclerosis_FSGS_WP2572_79296	0.3890894	1	74
Hs_Signaling_by_Insulin_receptor_WP1913_77046	0.39325944	1	75
Hs_Transcriptional_Regulation_of_White_Adipocyte_Differentiation_WP2751_76992	0.39325944	1	74
Hs_miR-targeted_genes_in_muscle_cell_-_TarBase_WP2005_78538	0.39825705	3	409
Hs_Fcgamma_receptor_(FCGR)_dependent_phagocytosis_WP2719_76936	0.40151456	1	210
Hs_Prolactin_Signaling_Pathway_WP2037_78501	0.4056	1	76
Hs_M-G1_Transition_WP2785_77074	0.4056	1	79
Hs_Apoptosis_Modulation_and_Signaling_WP1772_63162	0.42166564	1	93
Hs_Regulation_of_mRNA_Stability_by_Proteins_that_Bind_AU-rich_Elements_WP2733_76960	0.42166564	1	83
Hs_Metabolism_of_carbohydrates_WP1848_76833	0.43343005	1	87
Hs_MicroRNAs_in_cardiomyocyte_hypertrophy_WP1544_75258	0.43729824	1	104
Hs_Cytoplasmic_Ribosomal_Proteins_WP477_67139	0.44874552	1	88
Hs_Corticotropin-releasing_hormone_WP2355_78490	0.45996055	1	90
Hs_Corticotropin-releasing_hormone_WP2355_79562	0.4636481	1	91
Hs_B_Cell_Receptor_Signaling_Pathway_WP23_78566	0.47094807	1	94
Hs_DNA_Damage_Response_(only_ATM_dependent)_WP710_70109	0.4745608	1	97
Hs_RNA_Polymerase_II_Transcription_WP1906_76887	0.49917042	1	101
Hs_Senescence_and_Autophagy_WP615_71375	0.51604176	1	106
Hs_S_Phase_WP2772_77049	0.5387137	1	116
Hs_Regulation_of_Lipid_Metabolism_by_Peroxisome_proliferator-activated_receptor_alpha_(PPARalpha)_WP2797_77088	0.5387137	1	118
Hs_Spinal_Cord_Injury_WP2431_78556	0.5387137	1	116
Hs_Signaling_by_the_B_Cell_Receptor_(BCR)_WP2746_76984	0.5603264	1	239
Hs_mRNA_Processing_WP411_71369	0.575143	1	127
Hs_miR-targeted_genes_in_leukocytes_-_TarBase_WP2003_78572	0.5809292	1	160
Hs_Gastrin-CREB_signalling_pathway_via_PKC_and_MAPK_WP2664_76844	0.6060101	1	147
Hs_NRF2_pathway_WP2884_79616	0.61403316	1	143
Hs_Immunoregulatory_interactions_between_a_Lymphoid_and_a_non-Lymphoid_cell_WP1829_76993	0.61403316	1	292
Hs_NRF2_pathway_WP2884_79518	0.61403316	1	143
Hs_Regulation_of_toll-like_receptor_signaling_pathway_WP1449_77378	0.62189335	1	150
Hs_Regulation_of_toll-like_receptor_signaling_pathway_WP1449_79550	0.62447774	1	150
Hs_Regulation_of_Actin_Cytoskeleton_WP51_79526	0.63464046	1	149
Hs_Calcium_Regulation_in_the_Cardiac_Cell_WP536_78588	0.6420821	1	150
Hs_Metabolism_of_amino_acids_and_derivatives_WP2693_76898	0.71264243	1	184
Hs_GPCRs,_Class_A_Rhodopsin-like_WP455_79717	0.82232976	1	262
Hs_GPCRs,_Class_A_Rhodopsin-like_WP455_78510	0.82232976	1	262
Hs_GPCR_ligand_binding_WP1825_76977	0.9107055	1	371
Hs_GPCR_downstream_signaling_WP1824_76910	0.9298366	1	406

Table S4 Pathway analysis through WikiPathways in the fibroblast irradiated with UV-C (upregulation, 12hr, 5J/m^2)	p-value (high dose 12hr)	Matched Entities (high dose 12hr)	Pathway Entities of Experiment Type (high dose 12hr)
Hs_Cell_Cycle_WP179_70629	0	12	103
Hs_Mitotic_Metaphase_and_Anaphase_WP2757_77009	0	19	153
Hs_Mitotic_Prometaphase_WP2652_76819	0	18	98
Hs_Gastric_cancer_network_1_WP2361_77608	8.91E-10	7	28
Hs_Gastric_Cancer_Network_1_WP2361_79693	8.91E-10	7	29
Hs_APC-C-mediated_degradation_of_cell_cycle_proteins_WP1782_77060	4.06E-09	9	80
Hs_RB_in_Cancer_WP2446_78573	1.21E-08	9	87
Hs_Kinesins_WP1842_76861	9.64E-08	5	18
Hs_Cell_Cycle_Checkpoints_WP1775_76816	1.02E-07	9	115
Hs_ATM_Signaling_Pathway_WP2516_78531	2.64E-07	6	41
Hs_G1_to_S_cell_cycle_control_WP45_71377	8.77E-06	6	68
Hs_DNA_Damage_Response_WP707_78527	8.77E-06	6	68
Hs_Integrated_Pancreatic_Cancer_Pathway_WP2377_71228	1.32E-05	9	200
Hs_miRNA_Regulation_of_DNA_Damage_Response_WP1530_79564	1.43E-05	6	98
Hs_miRNA_Regulation_of_DNA_Damage_Response_WP1530_78503	1.43E-05	6	98
Hs_Nucleosome_assembly_WP1874_76826	1.59E-05	4	22
Hs_MHC_class_II_antigen_presentation_WP2679_76872	2.96E-05	6	90
Hs_IL-6_signaling_pathway_WP364_78561	2.39E-04	4	43
Hs_Regulation_of_Microtubule_Cytoskeleton_WP2038_78570	2.39E-04	4	44
Hs_Mitotic_G2-G2-M_phases_WP1859_77022	3.85E-04	5	89
Hs_Circadian_Clock_WP1797_76871	3.96E-04	4	49
Hs_Androgen_receptor_signaling_pathway_WP138_79277	4.27E-04	5	89
Hs_Apoptosis-related_network_due_to_altered_Notch3_in_ovarian_cancer_WP2864_79278	5.36E-04	4	53
Hs_ErbB_Signaling_Pathway_WP673_69914	5.75E-04	4	54
Hs_Oxidative_Stress_WP408_78546	0.001214302	3	30
Hs_AMPK_Signaling_WP1403_79471	0.001371507	4	68
Hs_Mitotic_G1-G1-S_phases_WP1858_76928	0.001525818	5	120
Hs_Glucocorticoid_Receptor_Pathway_WP2880_79615	0.001527266	4	71
Hs_Glucocorticoid_Receptor_Pathway_WP2880_79522	0.001527266	4	71
Hs_Benzo(a)pyrene_metabolism_WP696_72081	0.001716496	2	9
Hs_Interleukin-11_Signaling_Pathway_WP2332_79525	0.003683958	3	44
Hs_Mitotic_Telophase-Cytokinesis_WP2765_77030	0.004239273	2	14
Hs_Energy_Metabolism_WP1541_68947	0.004441011	3	47
Hs_DNA_Damage_Response_(only_ATM_dependent)_WP710_70109	0.004453771	4	97
Hs_Wnt_Signaling_Pathway_and_Pluripotency_WP399_79474	0.005542425	4	101
Hs_EGF-EGFR_Signaling_Pathway_WP437_79266	0.005937763	5	162
Hs_Senescence_and_Autophagy_WP615_71375	0.006795683	4	106
Hs_MAPK_Signaling_Pathway_WP382_72103	0.006900403	5	168
Hs_miRNAs_involved_in_DNA_damage_response_WP1545_78559	0.007783683	2	69
Hs_TP53_Network_WP1742_71700	0.009470919	2	22
Hs_Double-Strand_Break_Repair_WP1807_76953	0.009470919	2	21
Hs_MAP_kinase_activation_in_TLR_cascade_WP2792_78712	0.010369995	2	22
Hs_Endochondral_Ossification_WP474_72122	0.010454983	3	64
Hs_Physiological_and_Pathological_Hypertrophy_of_the_Heart_WP1528_78581	0.012276274	2	24
Hs_Adipogenesis_WP236_78584	0.013640647	4	131
Hs_Primary_Focal_Segmental_Glomerulosclerosis_FSGS_WP2572_79296	0.014364767	3	74
Hs_Transcriptional_Regulation_of_White_Adipocyte_Differentiation_WP2751_76992	0.01490397	3	74
Hs_Arachidonic_acid_metabolism_WP2650_76814	0.015397844	2	27
Hs_Prolactin_Signaling_Pathway_WP2037_78501	0.016589586	3	76
Hs_Selenium_Micronutrient_Network_WP15_78776	0.019626774	3	84
Hs_Gastric_cancer_network_2_WP2363_76329	0.020027012	2	32
Hs_Bladder_Cancer_WP2828_79529	0.020027012	2	31
Hs_MicroRNAs_in_cardiomyocyte_hypertrophy_WP1544_75258	0.021586332	3	104
Hs_Apoptosis_WP254_78808	0.021586332	3	84
Hs_TOR_Signaling_WP1471_70031	0.023831226	2	34
Hs_Nuclear_Receptors_Meta-Pathway_WP2882_78569	0.024037078	6	318
Hs_Integrated_Cancer_pathway_WP1971_71249	0.026518172	2	36
Hs_Nucleotide-binding_domain,_leucine_rich_repeat_containing_receptor_(NLR)_signaling_pathways_WP2763_77025	0.026518172	2	38
Hs_miR-targeted_genes_in_lymphocytes_-_TarBase_WP2004_78524	0.02716363	7	495
Hs_Insulin_Signaling_WP481_72080	0.027399216	4	161
Hs_DNA_Damage_Bypass_WP1803_76966	0.027818946	1	4
Hs_Interleukin-1_processing_WP1838_77045	0.027818946	1	4
Hs_BMI1_WP2612_73362	0.027818946	1	4
Hs_Integrated_Breast_Cancer_Pathway_WP1984_72732	0.029048383	4	164
Hs_G13_Signaling_Pathway_WP524_72112	0.029321264	2	38
Hs_IL-5_Signaling_Pathway_WP127_78498	0.032236736	2	40
Hs_Mitotic_Prophase_WP2654_76823	0.032236736	2	44
Hs_Mitochondrial_Iron-Sulfur_Cluster_Biogenesis_WP2702_76911	0.03465224	1	7
Hs_IL-2_Signaling_Pathway_WP49_78543	0.035260916	2	42
Hs_tRNA_Aminoacylation_WP1938_76923	0.035260916	2	42
Hs_JAK-STAT_WP2593_74127	0.036812637	2	45
Hs_JAK-STAT_WP2594_74128	0.036812637	2	45
Hs_Focal_Adhesion_WP306_78800	0.04221893	4	188
Hs_Focal_Adhesion_WP306_79698	0.043622565	4	188
Hs_Prostate_Cancer_WP2263_73838	0.043827046	3	115
Hs_Diurnally_Regulated_Genes_with_Circadian_Orthologs_WP410_69903	0.04495006	2	48
Hs_Interleukin-6_signaling_WP2704_76915	0.04817546	1	11
Hs_Wnt_Signaling_Pathway_Netpath_WP363_78571	0.05012024	2	51
Hs_Host_Interactions_of_HIV_factors_WP2684_76883	0.050912775	3	137
Hs_Regulation_of_Apoptosis_WP1896_77071	0.0518891	2	53
Hs_Degradation_of_beta-catenin_by_the_destruction_complex_WP2773_77050	0.053680025	2	55
Hs_Non-homologous_end_joining_WP438_68977	0.05486606	1	8
Hs_Effects_of_Nitric_Oxide_WP1995_69910	0.05486606	1	8
Hs_TFs_Regulate_miRNAs_related_to_cardiac_hypertrophy_WP1559_68890	0.05486606	1	15
Hs_IL-4_Signaling_Pathway_WP395_78576	0.05549262	2	55
Hs_TGF_beta_Signaling_Pathway_WP366_79341	0.06543453	3	131
Hs_Metabolism_of_nucleotides_WP1851_76838	0.066801645	2	65
Hs_Mammary_gland_development_pathway_-_Involution_(Stage_4_of_4)_WP2815_78062	0.068106875	1	10
Hs_Signaling_by_VEGF_WP1919_76864	0.068106875	1	10
Hs_RAF-MAP_kinase_cascade_WP2735_76963	0.068106875	1	10
Hs_Notch_Signaling_Pathway_WP61_78592	0.06875521	2	61
Hs_Semaphorin_interactions_WP1907_76850	0.07072744	2	62
Hs_DSCAM_interactions_WP1808_77007	0.07465775	1	11
Hs_Iron_metabolism_in_placenta_WP2007_69751	0.07465775	1	12
Hs_Proteasome_Degradation_WP183_71712	0.074726455	2	65
Hs_Oncostatin_M_Signaling_Pathway_WP2374_73668	0.07675254	2	65
Hs_BDNF_signaling_pathway_WP2380_79265	0.07774974	3	141
Hs_TSH_signaling_pathway_WP2032_78548	0.07879589	2	66
Hs_Wnt_Signaling_Pathway_WP428_79528	0.07879589	2	67
Hs_Regulation_of_DNA_replication_WP1898_76824	0.080856144	2	70
Hs_Endoderm_Differentiation_WP2853_78496	0.08293298	2	146
Hs_PIP3_activates_AKT_signaling_WP2653_76821	0.087135054	2	90
Hs_Estrogen_Receptor_Pathway_WP2881_79519	0.08762205	1	13
Hs_SRF_and_miRs_in_Smooth_Muscle_Differentiation_and_Proliferation_WP1991_75261	0.08762205	1	17
Hs_Estrogen_Receptor_Pathway_WP2881_79603	0.08762205	1	13
Hs_Cell_Differentiation_-_Index_WP2029_69036	0.100405194	1	54
Hs_Signaling_Pathways_in_Glioblastoma_WP2261_78522	0.11356959	2	82
Hs_miR-targeted_genes_in_adipocytes_-_TarBase_WP2001_78529	0.11924579	1	38
Hs_MyD88_cascade_initiated_on_plasma_membrane_WP2801_77090	0.11924579	1	19
Hs_Cytoplasmic_Ribosomal_Proteins_WP477_67139	0.12512171	2	88

Hs_Signaling_by_Hippo_WP2714_76930	0.12543812	1	20
Hs_Mitochondrial_Gene_Expression_WP391_71373	0.12543812	1	19
Hs_Base_Excision_Repair_WP1787_78301	0.12543812	1	19
Hs_Serotonin_Receptor_4-6-7_and_NR3C_Signaling_WP734_74438	0.12543812	1	19
Hs_Eicosanoid_Synthesis_WP167_71381	0.13158704	1	25
Hs_Synthesis_of_DNA_WP1925_76968	0.13455595	2	94
Hs_Influenza_Life_Cycle_WP2683_76880	0.13724035	3	217
Hs_MyD88_dependent_cascade_initiated_on_endosome_WP2768_77040	0.13769285	1	21
Hs_B_Cell_Receptor_Signaling_Pathway_WP23_78566	0.1393315	2	94
Hs_Cell_Differentiation_-_meta_WP2023_68892	0.14375585	1	67
Hs_Detoxification_of_Reactive_Oxygen_Species_WP2824_76144	0.14375585	1	26
Hs_GPCRs,_Class_B_Secretin-like_WP334_79716	0.14977632	1	23
Hs_Processing_of_Capped_Intronless_Pre-mRNA_WP1890_76858	0.14977632	1	23
Hs_GPCRs,_Class_B_Secretin-like_WP334_63204	0.14977632	1	23
Hs_TAK1_activates_NFkB_by_phosphorylation_and_activation_of_IKKs_complex_WP2656_76831	0.14977632	1	24
Hs_Neural_Crest_Differentiation_WP2064_79263	0.15386812	2	101
Hs_Signal_Transduction_of_S1P_Receptor_WP26_78492	0.1557546	1	25
Hs_RNA_Polymerase_II_Transcription_WP1906_76887	0.15877733	2	101
Hs_YAP1-_and_WWTR1_(TAZ)-stimulated_gene_expression_WP2738_76971	0.16169094	1	25
Hs_IL-7_Signaling_Pathway_WP205_79472	0.16169094	1	25
Hs_Post-translational_modification-_synthesis_of_GPI-anchored_proteins_WP1887_77093	0.16169094	1	26
Hs_Regulatory_RNA_pathways_WP1901_76851	0.16758564	1	26
Hs_PPAR_Alpha_Pathway_WP2878_79689	0.16758564	1	26
Hs_PPAR_alpha_pathway_WP2878_79532	0.16758564	1	26
Hs_EPO_Receptor_Signaling_WP581_67199	0.16758564	1	26
Hs_Arylhydrocarbon_receptor_(AhR)_signaling_pathway_WP2100_74081	0.17343903	1	28
Hs_FSH_signaling_pathway_WP2035_78536	0.17343903	1	27
Hs_MyD88-Mal_cascade_initiated_on_plasma_membrane_WP2761_77020	0.17343903	1	28
Hs_Interleukin-2_signaling_WP2732_76959	0.17925136	1	29
Hs_T-Cell_Receptor_and_Co-stimulatory_Signaling_WP2583_75360	0.17925136	1	32
Hs_Nanoparticle-mediated_activation_of_receptor_signaling_WP2643_74251	0.17925136	1	28
Hs_Meiotic_Recombination_WP2698_76904	0.18502292	1	29
Hs_S_Phase_WP2772_77049	0.18877673	2	116
Hs_Spinal_Cord_Injury_WP2431_78556	0.18877673	2	116
Hs_Regulation_of_Lipid_Metabolism_by_Peroxisome_proliferator-activated_receptor_alpha_(PPARalpha)_WP2797_77088	0.18877673	2	118
Hs_Generic_Transcription_Pathway_WP1822_77033	0.19075401	1	30
Hs_Dopaminergic_Neurogenesis_WP2855_79211	0.19075401	1	30
Hs_miRs_in_Muscle_Cell_Differentiation_WP2012_68959	0.19644491	1	40
Hs_IL17_signaling_pathway_WP2112_63216	0.19644491	1	31
Hs_Inflammatory_Response_Pathway_WP453_63217	0.2020959	1	33
Hs_Ovarian_Infertility_Genes_WP34_72115	0.2020959	1	32
Hs_Monoamine_Transport_WP727_68928	0.2020959	1	32
Hs_Meiotic_Synapsis_WP2731_76957	0.20770726	1	35
Hs_Protein_folding_WP1892_76909	0.20770726	1	33
Hs_Pre-NOTCH_Expression_and_Processing_WP2786_77076	0.21327926	1	38
Hs_p38_MAPK_Signaling_Pathway_WP400_72084	0.21327926	1	34
Hs_mRNA_Processing_WP411_71369	0.2194585	2	127
Hs_Glycogen_Metabolism_WP500_63201	0.2243063	1	36
Hs_Interleukin-3,_5_and_GM-CSF_signaling_WP1840_77073	0.2243063	1	37
Hs_Interleukin-1_signaling_WP1839_76943	0.22976187	1	38
Hs_Ectoderm_Differentiation_WP2858_78578	0.23495743	2	145
Hs_Ectoderm_Commitment_Pathway_WP2856_78535	0.23495743	2	145
Hs_Nuclear_Receptors_WP170_71083	0.23517919	1	38
Hs_FAS_pathway_and_Stress_induction_of_HSP_regulation_WP314_71366	0.23517919	1	38
Hs_Interferon_gamma_signaling_WP1836_77096	0.23517919	1	43
Hs_Amyotrophic_lateral_sclerosis_(ALS)_WP2447_75221	0.23517919	1	38
Hs_GPCR_ligand_binding_WP1825_76977	0.23608747	4	371
Hs_Transport_of_inorganic_cations-anions_and_amino_acids-oligopeptides_WP1936_76845	0.2405585	1	43
Hs_Vitamin_A_and_Carotenoid_Metabolism_WP716_74442	0.2405585	1	43
Hs_Processing_of_Capped_Intron-Containing_Pre-mRNA_WP1889_77003	0.2453248	2	144
Hs_NOD_pathway_WP1433_68991	0.2459001	1	41
Hs_Glycosaminoglycan_metabolism_WP2743_76981	0.2512042	1	41
Hs_Integrated_Lung_Cancer_Pathway_WP2512_71235	0.2512042	1	44
Hs_Parkinsons_Disease_Pathway_WP2371_79311	0.2512042	1	62
Hs_TWEAK_Signaling_Pathway_WP2036_78525	0.2512042	1	41
Hs_Interferon_alpha-beta_signaling_WP1835_77078	0.2564711	1	45
Hs_GPCRs,_Class_A_Rhodopsin-like_WP455_78510	0.2612892	3	262
Hs_GPCRs,_Class_A_Rhodopsin-like_WP455_79717	0.2612892	3	262
Hs_Hair_Follicle_Development-_Induction_(Part_1_of_3)_WP2804_78710	0.26170108	1	44
Hs_Sphingolipid_metabolism_WP2788_77079	0.26170108	1	46
Hs_Regulation_of_toll-like_receptor_signaling_pathway_WP1449_77378	0.2635026	2	150
Hs_Regulation_of_toll-like_receptor_signaling_pathway_WP1449_79550	0.26610085	2	150
Hs_Apoptotic_execution_phase_WP1784_76813	0.26689434	1	46
Hs_Heart_Development_WP1591_78590	0.26689434	1	47
Hs_Tryptophan_metabolism_WP465_77387	0.27717188	1	80
Hs_Selenium_Metabolism_and_Selenoproteins_WP28_71888	0.27717188	1	48
Hs_Notch_Signaling_Pathway_WP268_70096	0.27717188	1	46
Hs_Tryptophan_metabolism_WP465_79226	0.27717188	1	80
Hs_Aryl_Hydrocarbon_Receptor_WP2586_78547	0.27717188	1	47
Hs_Structural_Pathway_of_Interleukin_1_(IL-1)_WP2637_76330	0.2822566	1	49
Hs_Structural_Pathway_of_Interleukin_1_(IL-1)_WP2637_79580	0.2822566	1	49
Hs_Calcium_Regulation_in_the_Cardiac_Cell_WP536_78588	0.28428036	2	150
Hs_miR-targeted_genes_in_epithelium_-_TarBase_WP2002_78530	0.3050162	3	345
Hs_Cardiac_Progenitor_Differentiation_WP2406_73324	0.30715024	1	53
Hs_Interferon_type_I_signaling_pathways_WP585_79096	0.3168649	1	54
Hs_NGF_signalling_via_TRKA_from_the_plasma_membrane_WP1873_76973	0.3168649	1	57
Hs_Cardiac_Hypertrophic_Response_WP2795_78544	0.3168649	1	54
Hs_SIDS_Susceptibility_Pathways_WP706_78533	0.31789777	2	166
Hs_IL-1_signaling_pathway_WP195_78528	0.32167116	1	55
Hs_Signaling_by_Type_1_Insulin-like_Growth_Factor_1_Receptor_(IGF1R)_WP2677_76867	0.33118278	1	75
Hs_Activation_of_Gene_Expression_by_SREBP_(SREBF)_WP2706_76917	0.33118278	1	60
Hs_Costimulation_by_the_CD28_family_WP1799_77064	0.33118278	1	71
Hs_Glycerophospholipid_biosynthesis_WP2740_76974	0.33588856	1	59
Hs_Kit_receptor_signaling_pathway_WP304_78799	0.34056136	1	59
Hs_Metapathway_biotransformation_WP702_73516	0.34605414	2	188
Hs_Metabolism_of_water-soluble_vitamins_and_cofactors_WP1857_76875	0.3543839	1	67
Hs_Leptin_signaling_pathway_WP2034_79545	0.3543839	1	62
Hs_TCR_signaling_WP1927_76950	0.3589269	1	74
Hs_Folate_Metabolism_WP176_74202	0.363438	1	67
Hs_Histone_Modifications_WP2369_69927	0.363438	1	67
Hs_Metabolism_of_amino_acids_and_derivatives_WP2693_76898	0.36629212	2	184
Hs_Visual_phototransduction_WP2776_77056	0.36791748	1	67
Hs_Mesodermal_Commitment_Pathway_WP2857_78577	0.3811681	1	154
Hs_L1CAM_interactions_WP1843_76876	0.3941417	1	75
Hs_Parkin-Ubiquitin_Proteasomal_System_pathway_WP2359_72121	0.3941417	1	73
Hs_Peptide_GPCRs_WP24_79444	0.3984057	1	73
Hs_Signaling_by_Insulin_receptor_WP1913_77046	0.40263975	1	75
Hs_Membrane_Trafficking_WP1846_76873	0.40684408	1	78
Hs_M-G1_Transition_WP2785_77074	0.4151644	1	79
Hs_miR-targeted_genes_in_muscle_cell_-_TarBase_WP2005_78538	0.41711542	3	409
Hs_Hair_Follicle_Development-_Cytodifferentiation_(Part_3_of_3)_WP2840_78512	0.4314575	1	87
Hs_Apoptosis_Modulation_and_Signaling_WP1772_63162	0.4314575	1	93
Hs_Regulation_of_mRNA_Stability_by_Proteins_that_Bind_AU-rich_Elements_WP2733_76960	0.4314575	1	83

Hs_Metabolism_of_carbohydrates_WP1848_76833	0.44337955	1	87
Hs_Eukaryotic_Translation_Termination_WP1813_77051	0.44337955	1	88
Hs_Fc_epsilon_receptor_(FCERI)_signaling_WP2759_77017	0.45505226	1	218
Hs_Eukaryotic_Translation_Elongation_WP1811_77053	0.45505226	1	91
Hs_Alzheimers_Disease_WP2059_79302	0.45505226	1	146
Hs_TNF_alpha_Signaling_Pathway_WP2808_78568	0.45505226	1	87
Hs_TNF_alpha_Signaling_Pathway_WP231_79280	0.46648085	1	90
Hs_Respiratory_electron_transport,_ATP_synthesis_by_chemiosmotic_coupling,_and_heat_production_by_uncoupling_proteins._WP1902_77091	0.47023702	1	94
Hs_Corticotropin-releasing_hormone_WP2355_78490	0.47023702	1	90
Hs_Corticotropin-releasing_hormone_WP2355_79562	0.4739668	1	91
Hs_Integrin-mediated_Cell_Adhesion_WP185_71391	0.5028776	1	99
Hs_Electron_Transport_Chain_WP111_79220	0.5028776	1	104
Hs_GPCR_downstream_signaling_WP1824_76910	0.5125201	3	406
Hs_Toll-like_receptor_signaling_pathway_WP75_72133	0.5133058	1	102
Hs_SRP-dependent_cotranslational_protein_targeting_to_membrane_WP2737_76970	0.5201364	1	111
Hs_Nonsense-Mediated_Decay_WP2710_76924	0.5235158	1	111
Hs_Eukaryotic_Translation_Initiation_WP1812_76969	0.5432995	1	117
Hs_Neurotransmitter_Receptor_Binding_And_Downstream_Transmission_In_The_Postsynaptic_Cell_WP2754_77001	0.5684092	1	124
Hs_Signaling_by_the_B_Cell_Receptor_(BCR)_WP2746_76984	0.5714495	1	239
Hs_miR-targeted_genes_in_squamous_cell_-_TarBase_WP2006_78523	0.5833987	1	160
Hs_HIV_Life_Cycle_WP2658_76836	0.592142	1	143
Hs_miR-targeted_genes_in_leukocytes_-_TarBase_WP2003_78572	0.592142	1	160
Hs_Class_I_MHC_mediated_antigen_processing_&_presentation_WP2796_77098	0.60908335	1	148
Hs_Gastrin-CREB_signalling_pathway_via_PKC_and_MAPK_WP2664_76844	0.617289	1	147
Hs_Regulation_of_Actin_Cytoskeleton_WP51_79526	0.64593375	1	149
Hs_Myometrial_Relaxation_and_Contraction_Pathways_WP289_78540	0.66541374	1	156

Table S5 GO LUV 4hr UP

<table>
<tr><th>GO ACCESSION</th><th>GO Term</th><th>p-value</th><th>corrected p-value</th><th>Count in Selection</th><th>% Count in Selection</th><th>Count in Total</th><th>% Count in Total</th></tr>
<tr><td>GO:0071294</td><td>cellular response to zinc ion</td><td>5.82E-11</td><td>1.89E-06</td><td>6</td><td>4.0816326</td><td>10</td><td>0.05536179</td></tr>
<tr><td>GO:0008284</td><td>positive regulation of cell proliferation</td><td>5.51E-09</td><td>8.95E-05</td><td>24</td><td>16.32653</td><td>722</td><td>3.997121</td></tr>
<tr><td>GO:0071347</td><td>cellular response to interleukin-1</td><td>1.93E-07</td><td>0.00208726</td><td>7</td><td>4.7619047</td><td>51</td><td>0.28234512</td></tr>
<tr><td>GO:0048519|GO:0043118</td><td>negative regulation of biological process</td><td>3.72E-07</td><td>0.003018721</td><td>58</td><td>39.455784</td><td>3780</td><td>20.926756</td></tr>
<tr><td>GO:0071248</td><td>cellular response to metal ion</td><td>7.77E-07</td><td>0.005049835</td><td>8</td><td>5.442177</td><td>90</td><td>0.49825612</td></tr>
<tr><td>GO:0010043</td><td>response to zinc ion</td><td>1.55E-06</td><td>0.007484095</td><td>6</td><td>4.0816326</td><td>44</td><td>0.24359187</td></tr>
<tr><td>GO:0071241</td><td>cellular response to inorganic substance</td><td>1.61E-06</td><td>0.007484095</td><td>8</td><td>5.442177</td><td>99</td><td>0.5480817</td></tr>
<tr><td>GO:0030335</td><td>positive regulation of cell migration</td><td>2.72E-06</td><td>0.008125031</td><td>12</td><td>8.163265</td><td>272</td><td>1.5058407</td></tr>
<tr><td>GO:0042127</td><td>regulation of cell proliferation</td><td>2.93E-06</td><td>0.008125031</td><td>28</td><td>19.047619</td><td>1314</td><td>7.274539</td></tr>
<tr><td>GO:0071276</td><td>cellular response to cadmium ion</td><td>3.00E-06</td><td>0.008125031</td><td>4</td><td>2.7210884</td><td>13</td><td>0.07197033</td></tr>
<tr><td>GO:2000147</td><td>positive regulation of cell motility</td><td>3.04E-06</td><td>0.008125031</td><td>12</td><td>8.163265</td><td>275</td><td>1.5224493</td></tr>
<tr><td>GO:0023057</td><td>negative regulation of signaling</td><td>3.08E-06</td><td>0.008125031</td><td>22</td><td>14.965986</td><td>877</td><td>4.855229</td></tr>
<tr><td>GO:0010648</td><td>negative regulation of cell communication</td><td>3.25E-06</td><td>0.008125031</td><td>22</td><td>14.965986</td><td>880</td><td>4.8718376</td></tr>
<tr><td>GO:0070555</td><td>response to interleukin-1</td><td>3.65E-06</td><td>0.008476263</td><td>7</td><td>4.7619047</td><td>78</td><td>0.43182194</td></tr>
<tr><td>GO:0040017</td><td>positive regulation of locomotion</td><td>4.09E-06</td><td>0.008847514</td><td>12</td><td>8.163265</td><td>283</td><td>1.5667386</td></tr>
<tr><td>GO:0051272</td><td>positive regulation of cellular component movement</td><td>4.39E-06</td><td>0.00891483</td><td>12</td><td>8.163265</td><td>285</td><td>1.577811</td></tr>
<tr><td>GO:0070887</td><td>cellular response to chemical stimulus</td><td>7.43E-06</td><td>0.013985047</td><td>36</td><td>24.489796</td><td>2045</td><td>11.3214855</td></tr>
<tr><td>GO:1902533|GO:0010740</td><td>positive regulation of intracellular signal transduction</td><td>7.75E-06</td><td>0.013985047</td><td>19</td><td>12.92517</td><td>721</td><td>3.991585</td></tr>
<tr><td>GO:0046686</td><td>response to cadmium ion</td><td>1.44E-05</td><td>0.024605924</td><td>5</td><td>3.4013605</td><td>38</td><td>0.2103748</td></tr>
<tr><td>GO:0009967|GO:0035468</td><td>positive regulation of signal transduction</td><td>1.87E-05</td><td>0.029376771</td><td>23</td><td>15.646258</td><td>1058</td><td>5.8572774</td></tr>
<tr><td>GO:0048523|GO:0051243</td><td>negative regulation of cellular process</td><td>1.97E-05</td><td>0.029376771</td><td>50</td><td>34.013607</td><td>3452</td><td>19.11089</td></tr>
<tr><td>GO:0007517</td><td>muscle organ development</td><td>1.99E-05</td><td>0.029376771</td><td>11</td><td>7.482993</td><td>278</td><td>1.5390577</td></tr>
<tr><td>GO:0023056</td><td>positive regulation of signaling</td><td>2.85E-05</td><td>0.03915654</td><td>24</td><td>16.32653</td><td>1164</td><td>6.4441123</td></tr>
<tr><td>GO:0030334</td><td>regulation of cell migration</td><td>3.01E-05</td><td>0.03915654</td><td>14</td><td>9.523809</td><td>463</td><td>2.5632508</td></tr>
<tr><td>GO:0010647</td><td>positive regulation of cell communication</td><td>3.01E-05</td><td>0.03915654</td><td>24</td><td>16.32653</td><td>1168</td><td>6.466257</td></tr>
<tr><td>GO:1990267</td><td>response to transition metal nanoparticle</td><td>3.97E-05</td><td>0.04956004</td><td>7</td><td>4.7619047</td><td>112</td><td>0.62005204</td></tr>
<tr><td>GO:0051240</td><td>positive regulation of multicellular organismal process</td><td>4.55E-05</td><td>0.054729994</td><td>23</td><td>15.646258</td><td>1120</td><td>6.2005205</td></tr>
<tr><td>GO:0070851</td><td>growth factor receptor binding</td><td>4.97E-05</td><td>0.057628818</td><td>7</td><td>4.7619047</td><td>116</td><td>0.6421968</td></tr>
<tr><td>GO:2000145</td><td>regulation of cell motility</td><td>5.21E-05</td><td>0.05836956</td><td>14</td><td>9.523809</td><td>487</td><td>2.696119</td></tr>
<tr><td>GO:0071310</td><td>cellular response to organic substance</td><td>5.44E-05</td><td>0.058900703</td><td>29</td><td>19.727892</td><td>1624</td><td>8.990754</td></tr>
<tr><td>GO:0019100</td><td>male germ-line sex determination</td><td>6.76E-05</td><td>0.06861757</td><td>2</td><td>1.3605442</td><td>2</td><td>0.011072358</td></tr>
<tr><td>GO:0007542</td><td>primary sex determination, germ-line</td><td>6.76E-05</td><td>0.06861757</td><td>2</td><td>1.3605442</td><td>2</td><td>0.011072358</td></tr>
<tr><td>GO:0060348</td><td>bone development</td><td>7.21E-05</td><td>0.0709954</td><td>7</td><td>4.7619047</td><td>123</td><td>0.68095</td></tr>
<tr><td>GO:0061061</td><td>muscle structure development</td><td>8.87E-05</td><td>0.08473454</td><td>13</td><td>8.843537</td><td>448</td><td>2.4802082</td></tr>
<tr><td>GO:0031328</td><td>positive regulation of cellular biosynthetic process</td><td>9.97E-05</td><td>0.090795346</td><td>26</td><td>17.687075</td><td>1425</td><td>7.889055</td></tr>
<tr><td>GO:0048584</td><td>positive regulation of response to stimulus</td><td>1.03E-04</td><td>0.090795346</td><td>27</td><td>18.367348</td><td>1512</td><td>8.370703</td></tr>
<tr><td>GO:1903036</td><td>positive regulation of response to wounding</td><td>1.07E-04</td><td>0.090795346</td><td>7</td><td>4.7619047</td><td>131</td><td>0.72523946</td></tr>
<tr><td>GO:0051051</td><td>negative regulation of transport</td><td>1.10E-04</td><td>0.090795346</td><td>11</td><td>7.482993</td><td>336</td><td>1.8601562</td></tr>
<tr><td>GO:0010038</td><td>response to metal ion</td><td>1.12E-04</td><td>0.090795346</td><td>10</td><td>6.802721</td><td>280</td><td>1.5501301</td></tr>
<tr><td>GO:0040012</td><td>regulation of locomotion</td><td>1.13E-04</td><td>0.090795346</td><td>14</td><td>9.523809</td><td>524</td><td>2.9009578</td></tr>
<tr><td>GO:0070498</td><td>interleukin-1-mediated signaling pathway</td><td>1.15E-04</td><td>0.090795346</td><td>3</td><td>2.0408163</td><td>12</td><td>0.066434145</td></tr>
<tr><td>GO:0003170</td><td>heart valve development</td><td>1.18E-04</td><td>0.0909986</td><td>4</td><td>2.7210884</td><td>31</td><td>0.17162155</td></tr>
<tr><td>GO:0006357|GO:0006358|GO:0010551</td><td>regulation of transcription from RNA polymerase II promoter</td><td>1.24E-04</td><td>0.092219815</td><td>26</td><td>17.687075</td><td>1444</td><td>7.994242</td></tr>
<tr><td>GO:0009891</td><td>positive regulation of biosynthetic process</td><td>1.32E-04</td><td>0.092219815</td><td>26</td><td>17.687075</td><td>1450</td><td>8.027459</td></tr>
<tr><td>GO:0051171</td><td>regulation of nitrogen compound metabolic process</td><td>1.32E-04</td><td>0.092219815</td><td>57</td><td>38.77551</td><td>4431</td><td>24.53081</td></tr>
<tr><td>GO:0045944|GO:0010552|GO:0045817</td><td>positive regulation of transcription from RNA polymerase II promoter</td><td>1.33E-04</td><td>0.092219815</td><td>18</td><td>12.244898</td><td>814</td><td>4.5064497</td></tr>
<tr><td>GO:0006355|GO:0032583|GO:0045449|GO:0061019</td><td>regulation of transcription, DNA-templated</td><td>1.33E-04</td><td>0.092219815</td><td>46</td><td>31.292517</td><td>3304</td><td>18.291534</td></tr>
<tr><td>GO:1903508</td><td>positive regulation of nucleic acid-templated transcription</td><td>1.45E-04</td><td>0.09552005</td><td>22</td><td>14.965986</td><td>1129</td><td>6.250346</td></tr>
<tr><td>GO:0045893|GO:0043193|GO:0045941|GO:0061020</td><td>positive regulation of transcription, DNA-templated</td><td>1.45E-04</td><td>0.09552005</td><td>22</td><td>14.965986</td><td>1129</td><td>6.250346</td></tr>
<tr><td>GO:0030238</td><td>male sex determination</td><td>1.48E-04</td><td>0.09552005</td><td>3</td><td>2.0408163</td><td>13</td><td>0.07197033</td></tr>
<tr><td>GO:0042221</td><td>response to chemical</td><td>1.50E-04</td><td>0.09552005</td><td>46</td><td>31.292517</td><td>3320</td><td>18.380114</td></tr>
<tr><td>GO:1903506</td><td>regulation of nucleic acid-templated transcription</td><td>1.57E-04</td><td>0.09716935</td><td>46</td><td>31.292517</td><td>3326</td><td>18.413332</td></tr>
<tr><td>GO:0042325</td><td>regulation of phosphorylation</td><td>1.59E-04</td><td>0.09716935</td><td>22</td><td>14.965986</td><td>1136</td><td>6.289099</td></tr>
<tr><td>GO:0051252</td><td>regulation of RNA metabolic process</td><td>1.63E-04</td><td>0.09731197</td><td>47</td><td>31.97279</td><td>3432</td><td>19.000166</td></tr>
<tr><td>GO:0010604</td><td>positive regulation of macromolecule metabolic process</td><td>1.66E-04</td><td>0.09731197</td><td>34</td><td>23.129251</td><td>2180</td><td>12.06887</td></tr>
<tr><td>GO:0060255</td><td>regulation of macromolecule metabolic process</td><td>1.68E-04</td><td>0.09731197</td><td>62</td><td>42.176872</td><td>5003</td><td>27.697504</td></tr>
<tr><td>GO:0001047</td><td>core promoter binding</td><td>1.71E-04</td><td>0.09733085</td><td>5</td><td>3.4013605</td><td>63</td><td>0.34877926</td></tr>
</table>

Table S6 GO HUV 4hr UP

GO ACCESSION	GO Term	p-value	corrected p-value	Count in Selection	% Count in Selection	Count in Total	% Count in Total
GO:0005730	nucleolus	4.56E-12	2.12E-07	85	20.286396	1685	9.328462
GO:0005634	nucleus	5.71E-11	1.32E-06	213	50.835323	6394	35.398327
GO:0044428	nuclear part	4.36E-10	5.28E-06	127	30.310263	3249	17.987045
GO:0031974	membrane-enclosed lumen	5.69E-10	5.28E-06	136	32.458233	3583	19.836128
GO:0090304	nucleic acid metabolic process	5.56E-10	5.28E-06	150	35.799522	4092	22.654045
GO:0043233	organelle lumen	8.49E-10	6.42E-06	134	31.980907	3532	19.553783
GO:0044260|GO:0034960	cellular macromolecule metabolic process	1.11E-09	6.42E-06	213	50.835323	6584	36.450203
GO:0070013	intracellular organelle lumen	9.85E-10	6.42E-06	132	31.50358	3468	19.199469
GO:0043170|GO:0043283	macromolecule metabolic process	1.35E-09	6.99E-06	231	55.131264	7341	40.64109
GO:0031981	nuclear lumen	1.73E-09	8.01E-06	114	27.207638	2862	15.844544
GO:0042254|GO:0007046	ribosome biogenesis	3.00E-09	1.27E-05	21	5.0119333	188	1.0408016
GO:0010467	gene expression	3.74E-09	1.45E-05	138	32.935562	3757	20.799425
GO:0016070	RNA metabolic process	4.68E-09	1.67E-05	129	30.78759	3443	19.061064
GO:0022613	ribonucleoprotein complex biogenesis	2.16E-08	7.16E-05	25	5.966587	291	1.6110281
GO:0071294	cellular response to zinc ion	2.96E-08	9.16E-05	6	1.4319808	10	0.05536179
GO:0003676	nucleic acid binding	8.62E-08	2.50E-04	129	30.78759	3610	19.985605
GO:0034470	ncRNA processing	1.75E-07	4.77E-04	22	5.2505965	258	1.4283341
GO:0016072	rRNA metabolic process	2.05E-07	5.30E-04	16	3.8186157	142	0.7861374
GO:0004062	aryl sulfotransferase activity	3.51E-07	7.43E-04	5	1.1933174	8	0.044289432
GO:1990267	response to transition metal nanoparticle	3.29E-07	7.43E-04	14	3.3412888	112	0.62005204
GO:0031428	box C/D snoRNP complex	3.51E-07	7.43E-04	5	1.1933174	8	0.044289432
GO:0006139|GO:0055134	nucleobase-containing compound metabolic process	3.52E-07	7.43E-04	164	39.140812	5033	27.863588
GO:1901360	organic cyclic compound metabolic process	4.88E-07	9.43E-04	174	41.527447	5451	30.177711
GO:0034641	cellular nitrogen compound metabolic process	4.69E-07	9.43E-04	174	41.527447	5448	30.161102
GO:0044452	nucleolar part	6.29E-07	0.001162997	9	2.1479714	44	0.24359187
GO:0006364|GO:0006365	rRNA processing	6.51E-07	0.001162997	15	3.5799522	136	0.7529203
GO:0006725	cellular aromatic compound metabolic process	9.70E-07	0.001668842	167	39.8568	5227	28.937607
GO:0043228	non-membrane-bounded organelle	1.11E-06	0.001778135	138	32.935562	4114	22.77584
GO:0043232	intracellular non-membrane-bounded organelle	1.11E-06	0.001778135	138	32.935562	4114	22.77584
GO:0046483	heterocycle metabolic process	1.36E-06	0.00211133	166	39.618137	5214	28.865637
GO:0070761	pre-snoRNP complex	1.52E-06	0.002275435	5	1.1933174	10	0.05536179
GO:0006396|GO:0006394	RNA processing	2.43E-06	0.003519719	39	9.307876	750	4.1521344
GO:0009059|GO:0043284	macromolecule biosynthetic process	3.02E-06	0.004129788	123	29.355608	3619	20.03543
GO:0034660	ncRNA metabolic process	2.94E-06	0.004129788	24	5.7279234	353	1.9542712
GO:0006807	nitrogen compound metabolic process	4.17E-06	0.005531847	180	42.959427	5864	32.464153
GO:0034645|GO:0034961	cellular macromolecule biosynthetic process	6.97E-06	0.008745223	120	28.639618	3566	19.742014
GO:0010043	response to zinc ion	6.80E-06	0.008745223	8	1.9093078	44	0.24359187
GO:0033273	response to vitamin	8.40E-06	0.00999726	11	2.6252983	91	0.5037923
GO:0044237	cellular metabolic process	8.31E-06	0.00999726	252	60.1432	8946	49.526657
GO:0010038	response to metal ion	9.59E-06	0.010859615	20	4.7732697	280	1.5501301
GO:0017108	5'-flap endonuclease activity	9.54E-06	0.010859615	4	0.9546539	7	0.038753252
GO:0044238	primary metabolic process	1.01E-05	0.011154874	254	60.620525	9052	50.11349
GO:0030515	snoRNA binding	1.44E-05	0.015533246	6	1.4319808	24	0.13286829
GO:0009058	biosynthetic process	1.55E-05	0.016353603	145	34.606205	4586	25.388916
GO:0032774	RNA biosynthetic process	1.63E-05	0.016833777	94	22.434368	2666	14.759453
GO:0043231	intracellular membrane-bounded organelle	1.78E-05	0.017981345	277	66.10979	10123	56.04274
GO:0010332	response to gamma radiation	2.12E-05	0.02094262	8	1.9093078	51	0.28234512
GO:0046686	response to cadmium ion	2.37E-05	0.022883924	7	1.6706444	38	0.2103748
GO:0043227	membrane-bounded organelle	2.45E-05	0.023235474	278	66.34845	10200	56.469025
GO:0042127	regulation of cell proliferation	3.08E-05	0.028555527	54	12.887828	1314	7.274539
GO:0071704	organic substance metabolic process	3.20E-05	0.029128883	257	61.336517	9299	51.480927
GO:0048256	flap endonuclease activity	3.31E-05	0.02955354	4	0.9546539	9	0.04982561
GO:0008284	positive regulation of cell proliferation	3.55E-05	0.031094585	35	8.353222	722	3.997121
GO:0043229	intracellular organelle	4.32E-05	0.035448123	298	71.12172	11161	61.78929
GO:1901576	organic substance biosynthetic process	4.16E-05	0.035448123	141	33.65155	4514	24.990313
GO:0009812	flavonoid metabolic process	4.43E-05	0.035448123	5	1.1933174	18	0.09965122
GO:0044249	cellular biosynthetic process	4.32E-05	0.035448123	139	33.174225	4439	24.575098
GO:0005732	small nucleolar ribonucleoprotein complex	4.43E-05	0.035448123	5	1.1933174	18	0.09965122
GO:0071248	cellular response to metal ion	4.52E-05	0.03559815	10	2.3866348	90	0.49825612
GO:0016893	endonuclease activity, active with either ribo- or deoxyribonucleic acids and producing 5'-phosphomonoesters	4.67E-05	0.036123242	7	1.6706444	42	0.23251952
GO:1901362	organic cyclic compound biosynthetic process	4.83E-05	0.036748156	105	25.059666	3148	17.42789
GO:0033557	Slx1-Slx4 complex	4.91E-05	0.036748156	3	0.7159904	4	0.022144716
GO:0006351|GO:0006350|GO:0061018|GO:0061022	transcription, DNA-templated	5.14E-05	0.037285823	88	21.002386	2526	13.984388
GO:0097659	nucleic acid-templated transcription	5.14E-05	0.037285823	88	21.002386	2526	13.984388
GO:0043226	organelle	6.93E-05	0.049512714	300	71.599045	11302	62.569893
GO:0010212	response to ionizing radiation	7.50E-05	0.052795816	12	2.8639617	135	0.74738413
GO:0051923	sulfation	7.71E-05	0.053436138	5	1.1933174	20	0.11072358
GO:0010035	response to inorganic substance	8.78E-05	0.059924148	23	5.48926	407	2.2532248
GO:0044424	intracellular part	9.38E-05	0.06314337	332	79.236275	12822	70.984886
GO:0034654	nucleobase-containing compound biosynthetic process	9.81E-05	0.06505912	98	23.38902	2941	16.281902
GO:0071241	cellular response to inorganic substance	1.02E-04	0.06689481	10	2.3866348	99	0.5480817
GO:0009987|GO:0008151|GO:0050875	cellular process	1.04E-04	0.06689481	346	82.57757	13503	74.75503
GO:0060255	regulation of macromolecule metabolic process	1.16E-04	0.07258801	150	35.799522	5003	27.697504
GO:0009314	response to radiation	1.15E-04	0.07258801	24	5.7279234	442	2.4469912
GO:0010792	DNA double-strand break processing involved in repair via single-strand annealing	1.21E-04	0.07260063	3	0.7159904	5	0.027680894
GO:0003723	RNA binding	1.22E-04	0.07260063	43	10.262529	1021	5.6524386
GO:0030529	ribonucleoprotein complex	1.27E-04	0.07260063	34	8.114558	739	4.091236
GO:0072359	circulatory system development	1.24E-04	0.07260063	35	8.353222	769	4.257322
GO:0072358	cardiovascular system development	1.24E-04	0.07260063	35	8.353222	769	4.257322
GO:0007584	response to nutrient	1.26E-04	0.07260063	14	3.3412888	187	1.0352654
GO:0045002	double-strand break repair via single-strand annealing	1.21E-04	0.07260063	3	0.7159904	5	0.027680894
GO:2000112	regulation of cellular macromolecule biosynthetic process	1.28E-04	0.07260063	115	27.4463	3605	19.957926
GO:0051272	positive regulation of cellular component movement	1.31E-04	0.07305101	18	4.295943	285	1.577811
GO:0044271	cellular nitrogen compound biosynthetic process	1.52E-04	0.08416785	101	24.105011	3088	17.09572
GO:0071347	cellular response to interleukin-1	1.67E-04	0.09141753	7	1.6706444	51	0.28234512
GO:0071276	cellular response to cadmium ion	1.74E-04	0.09416636	4	0.9546539	13	0.07197033
GO:0019438	aromatic compound biosynthetic process	1.79E-04	0.094630234	99	23.627686	3026	16.752478
GO:0010556	regulation of macromolecule biosynthetic process	1.79E-04	0.094630234	117	27.923628	3711	20.54476
GO:0004518	nuclease activity	1.85E-04	0.09673282	14	3.3412888	194	1.0740187
GO:0034035	purine ribonucleoside bisphosphate metabolic process	1.96E-04	0.09989841	5	1.1933174	24	0.13286829
GO:0050427	3'-phosphoadenosine 5'-phosphosulfate metabolic process	1.96E-04	0.09989841	5	1.1933174	24	0.13286829

Table S7 GO LUV 12hr UP

GO ACCESSION	GO Term	p-value	corrected p-value	Count in Selection	% Count in Selection	Count in Total	% Count in Total
GO:0000278	mitotic cell cycle	0	0	85	33.86454	738	4.0857
GO:0000280	nuclear division	0	0	70	27.888447	455	2.5189614
GO:0007067	mitotic nuclear division	0	0	64	25.498009	318	1.7605048
GO:0022402	cell cycle process	0	0	90	35.856575	1004	5.558324
GO:0048285	organelle fission	0	0	71	28.286852	481	2.662902
GO:0051301	cell division	0	0	67	26.693228	466	2.5798595
GO:1903047	mitotic cell cycle process	0	0	81	32.270916	665	3.681559
GO:0007049	cell cycle	4.20E-45	1.58E-41	96	38.247013	1307	7.235786
GO:0000087	mitotic M phase	5.28E-34	1.77E-30	32	12.749004	108	0.5979073
GO:0007059	chromosome segregation	1.14E-32	3.44E-29	35	13.944223	154	0.85257155
GO:0000279	M phase	1.26E-31	3.45E-28	33	13.14741	138	0.76399267
GO:1902589	single-organism organelle organization	2.23E-31	5.60E-28	89	35.458168	1627	9.007363
GO:0000775|GO:0097521	chromosome, centromeric region	1.29E-28	3.00E-25	33	13.14741	168	0.9300781
GO:0005819	spindle	1.11E-27	2.40E-24	38	15.139442	263	1.4560151
GO:0022403	cell cycle phase	1.28E-27	2.57E-24	40	15.936255	302	1.671926
GO:0000776|GO:0005699	kinetochore	2.62E-27	4.93E-24	28	11.155378	114	0.6311244
GO:0044848	biological phase	3.46E-27	6.13E-24	41	16.334661	330	1.8269391
GO:0098687	chromosomal region	7.21E-27	1.21E-23	35	13.944223	222	1.2290317
GO:0000779	condensed chromosome, centromeric region	8.97E-27	1.42E-23	26	10.358565	95	0.525937
GO:0000793	condensed chromosome	1.05E-26	1.58E-23	33	13.14741	191	1.0574101
GO:0006996	organelle organization	1.36E-26	1.95E-23	102	40.63745	2456	13.596855
GO:0000777	condensed chromosome kinetochore	7.93E-26	1.09E-22	25	9.960159	91	0.5037923
GO:0000236	mitotic prometaphase	5.11E-25	6.70E-22	24	9.561753	86	0.47611138
GO:0007017	microtubule-based process	1.81E-24	2.28E-21	43	17.131474	434	2.4027016
GO:0000226	microtubule cytoskeleton organization	1.83E-22	2.13E-19	34	13.545816	277	1.5335215
GO:0000819	sister chromatid segregation	1.84E-22	2.13E-19	20	7.9681277	62	0.3432431
GO:0005694	chromosome	4.98E-22	5.56E-19	51	20.318726	729	4.0358744
GO:0015630	microtubule cytoskeleton	7.24E-21	7.79E-18	57	22.709164	974	5.392238
GO:0000070|GO:0016359	mitotic sister chromatid segregation	1.84E-20	1.91E-17	18	7.1713147	55	0.30448985
GO:0051983	regulation of chromosome segregation	3.48E-20	3.50E-17	19	7.569721	67	0.370924
GO:0044427	chromosomal part	6.95E-20	6.76E-17	45	17.928288	627	3.4711843
GO:0005874	microtubule	4.17E-18	3.92E-15	34	13.545816	379	2.0982118
GO:0051302	regulation of cell division	5.76E-18	5.26E-15	27	10.756972	221	1.2234956
GO:0044430	cytoskeletal part	3.69E-17	3.27E-14	60	23.904383	1288	7.1305985
GO:0007051	spindle organization	6.77E-17	5.82E-14	18	7.1713147	83	0.45950285
GO:0010564	regulation of cell cycle process	2.20E-16	1.85E-13	33	13.14741	404	2.2366164
GO:0051726|GO:0000074	regulation of cell cycle	3.33E-16	2.71E-13	44	17.52988	748	4.141062
GO:0043228	non-membrane-bounded organelle	4.73E-16	3.65E-13	115	45.816734	4114	22.77584
GO:0043232	intracellular non-membrane-bounded organelle	4.73E-16	3.65E-13	115	45.816734	4114	22.77584
GO:0000922|GO:0030615	spindle pole	1.16E-15	8.76E-13	19	7.569721	112	0.62005204
GO:0051276|GO:0007001|GO:0051277	chromosome organization	1.53E-15	1.12E-12	44	17.52988	780	4.3182197
GO:0007088	regulation of mitosis	2.29E-15	1.64E-12	19	7.569721	116	0.6421968
GO:0007346	regulation of mitotic cell cycle	3.93E-15	2.75E-12	29	11.553785	336	1.8601562
GO:0016043|GO:0044235|GO:0071842	cellular component organization	3.68E-14	2.52E-11	116	46.21514	4418	24.45884
GO:0051783	regulation of nuclear division	6.14E-14	4.11E-11	19	7.569721	138	0.76399267
GO:0071840|GO:0071841	cellular component organization or biogenesis	2.69E-13	1.76E-10	116	46.21514	4539	25.128716
GO:0005876	spindle microtubule	5.67E-13	3.63E-10	12	4.7808766	44	0.24359187
GO:0005634	nucleus	7.68E-13	4.82E-10	144	57.370518	6394	35.398327
GO:0005856	cytoskeleton	1.40E-12	8.64E-10	64	25.498009	1815	10.048164
GO:0010965	regulation of mitotic sister chromatid separation	2.33E-12	1.35E-09	12	4.7808766	49	0.27127278
GO:0033045	regulation of sister chromatid segregation	2.33E-12	1.35E-09	12	4.7808766	49	0.27127278
GO:0033047	regulation of mitotic sister chromatid segregation	2.33E-12	1.35E-09	12	4.7808766	49	0.27127278
GO:0000780	condensed nuclear chromosome, centromeric region	3.48E-12	1.98E-09	8	3.187251	14	0.077506505
GO:0007010	cytoskeleton organization	1.27E-11	7.07E-09	38	15.139442	777	4.301611
GO:0008017	microtubule binding	1.31E-11	7.20E-09	18	7.1713147	163	0.90239716
GO:0032886	regulation of microtubule-based process	6.37E-11	3.43E-08	15	5.9760957	115	0.6366606
GO:0031577	spindle checkpoint	1.32E-10	6.97E-08	10	3.9840639	40	0.22144715
GO:0000940	condensed chromosome outer kinetochore	1.47E-10	7.64E-08	7	2.7888446	13	0.07197033
GO:0005871	kinesin complex	1.69E-10	8.65E-08	11	4.38247	54	0.29895365
GO:0031145	anaphase-promoting complex-dependent proteasomal ubiquitin-dependent protein catabolic process	2.91E-10	1.46E-07	13	5.1792827	89	0.49271992
GO:0071822	protein complex subunit organization	3.14E-10	1.55E-07	48	19.123507	1294	7.1638155
GO:0030071	regulation of mitotic metaphase/anaphase transition	3.68E-10	1.76E-07	10	3.9840639	44	0.24359187
GO:1902099	regulation of metaphase/anaphase transition of cell cycle	3.68E-10	1.76E-07	10	3.9840639	44	0.24359187
GO:0030496	midbody	3.91E-10	1.84E-07	14	5.577689	110	0.6089797
GO:0007052	mitotic spindle organization	8.62E-10	3.99E-07	9	3.5856574	35	0.19376627
GO:0070507	regulation of microtubule cytoskeleton organization	8.74E-10	3.99E-07	13	5.1792827	97	0.53700936
GO:0044428	nuclear part	9.66E-10	4.35E-07	85	33.86454	3249	17.987045
GO:0031981	nuclear lumen	1.03E-09	4.55E-07	78	31.075697	2862	15.844544
GO:0033043	regulation of organelle organization	1.08E-09	4.71E-07	32	12.749004	669	3.7037036
GO:0071294	cellular response to zinc ion	1.36E-09	5.85E-07	6	2.3904383	10	0.05536179
GO:0005875	microtubule associated complex	1.59E-09	6.73E-07	15	5.9760957	144	0.79720974
GO:0003777	microtubule motor activity	1.61E-09	6.75E-07	12	4.7808766	83	0.45950285
GO:0044424	intracellular part	1.64E-09	6.79E-07	218	86.85259	12822	70.984886
GO:0051985	negative regulation of chromosome segregation	1.92E-09	7.82E-07	9	3.5856574	38	0.2103748
GO:0015631	tubulin binding	3.07E-09	1.23E-06	18	7.1713147	227	1.2567127
GO:0045931	positive regulation of mitotic cell cycle	3.22E-09	1.27E-06	12	4.7808766	88	0.48718375
GO:1901990	regulation of mitotic cell cycle phase transition	4.67E-09	1.83E-06	17	6.772908	206	1.1404529
GO:0000910|GO:0007104|GO:0016288|GO:0033205	cytokinesis	4.74E-09	1.83E-06	13	5.1792827	111	0.61451584
GO:1901987	regulation of cell cycle phase transition	5.81E-09	2.22E-06	17	6.772908	209	1.1570613
GO:0005622	intracellular	7.76E-09	2.92E-06	220	87.6494	13150	72.80075
GO:0044770	cell cycle phase transition	8.01E-09	2.94E-06	20	7.9681277	300	1.6608536
GO:0044772	mitotic cell cycle phase transition	8.01E-09	2.94E-06	20	7.9681277	300	1.6608536
GO:0006323	DNA packaging	8.63E-09	3.13E-06	16	6.374502	188	1.0408016
GO:0000075|GO:0031576|GO:0071779	cell cycle checkpoint	9.00E-09	3.21E-06	18	7.1713147	243	1.3452915
GO:0051303	establishment of chromosome localization	9.06E-09	3.21E-06	7	2.7888446	21	0.11625976
GO:0045787	positive regulation of cell cycle	9.59E-09	3.36E-06	18	7.1713147	244	1.3508277
GO:0000778	condensed nuclear chromosome kinetochore	1.02E-08	3.54E-06	5	1.9920319	7	0.038753252
GO:0005815	microtubule organizing center	1.11E-08	3.80E-06	27	10.756972	544	3.0116813
GO:0050000	chromosome localization	1.31E-08	4.45E-06	7	2.7888446	22	0.12179594
GO:0000794	condensed nuclear chromosome	1.35E-08	4.51E-06	11	4.38247	80	0.4428943
GO:0034508	centromere complex assembly	1.75E-08	5.80E-06	9	3.5856574	48	0.26573658
GO:0033044	regulation of chromosome organization	1.98E-08	6.49E-06	15	5.9760957	173	0.95775896
GO:0043229	intracellular organelle	2.02E-08	6.56E-06	196	78.08765	11161	61.78929
GO:0071173	spindle assembly checkpoint	2.12E-08	6.78E-06	8	3.187251	35	0.19376627
GO:0005813	centrosome	2.64E-08	7.99E-06	23	9.163346	422	2.3362675
GO:0033046	negative regulation of sister chromatid segregation	2.69E-08	7.99E-06	8	3.187251	36	0.19930243
GO:0033048	negative regulation of mitotic sister chromatid segregation	2.69E-08	7.99E-06	8	3.187251	36	0.19930243
GO:0045841	negative regulation of mitotic metaphase/anaphase transition	2.69E-08	7.99E-06	8	3.187251	36	0.19930243
GO:1902100	negative regulation of metaphase/anaphase transition of cell cycle	2.69E-08	7.99E-06	8	3.187251	36	0.19930243
GO:2000816	negative regulation of mitotic sister chromatid separation	2.69E-08	7.99E-06	8	3.187251	36	0.19930243
GO:0051988	regulation of attachment of spindle microtubules to kinetochore	2.70E-08	7.99E-06	5	1.9920319	8	0.044289432
GO:0005737	cytoplasm	2.70E-08	7.99E-06	179	71.31474	9839	54.470463
GO:0043161	proteasome-mediated ubiquitin-dependent protein catabolic process	2.96E-08	8.65E-06	16	6.374502	205	1.1349167
GO:0071174	mitotic spindle checkpoint	3.39E-08	9.82E-06	8	3.187251	37	0.20483862
GO:0043226	organelle	3.61E-08	1.04E-05	197	78.48605	11302	62.569893
GO:0031262	Ndc80 complex	3.64E-08	1.04E-05	4	1.5936255	4	0.022144716
GO:0010498	proteasomal protein catabolic process	3.88E-08	1.09E-05	16	6.374502	209	1.1570613
GO:0051781	positive regulation of cell division	4.20E-08	1.17E-05	12	4.7808766	110	0.6089797
GO:0007018	microtubule-based movement	6.03E-08	1.67E-05	15	5.9760957	188	1.0408016
GO:0043933|GO:0034600|GO:0034621	macromolecular complex subunit organization	1.12E-07	3.07E-05	54	21.513945	1859	10.291757
GO:0005829	cytosol	1.18E-07	3.19E-05	71	28.286852	2781	15.396113
GO:0071459	protein localization to chromosome, centromeric region	1.19E-07	3.19E-05	5	1.9920319	10	0.05536179

GO:0045839	negative regulation of mitosis	1.43E-07	3.82E-05	8	3.187251	44	0.24359187
GO:0051782	negative regulation of cell division	1.55E-07	4.09E-05	9	3.5856574	61	0.33770692
GO:0030261|GO:0000068	chromosome condensation	1.82E-07	4.77E-05	7	2.7888446	31	0.17162155
GO:0006259|GO:0055132	DNA metabolic process	1.98E-07	5.14E-05	34	13.545816	922	5.104357
GO:0043234	protein complex	2.63E-07	6.76E-05	88	35.05976	3835	21.231247
GO:0090068	positive regulation of cell cycle process	3.44E-07	8.79E-05	14	5.577689	186	1.0297292
GO:0007094	mitotic spindle assembly checkpoint	3.59E-07	9.10E-05	7	2.7888446	34	0.18823008
GO:0051383	kinetochore organization	3.64E-07	9.14E-05	5	1.9920319	12	0.066434145
GO:0010948	negative regulation of cell cycle process	3.93E-07	9.75E-05	15	5.9760957	217	1.2013508
GO:0071103	DNA conformation change	3.95E-07	9.75E-05	17	6.772908	279	1.5445939
GO:0032435	negative regulation of proteasomal ubiquitin-dependent protein catabolic process	4.03E-07	9.88E-05	8	3.187251	50	0.27680895
GO:1901799	negative regulation of proteasomal protein catabolic process	4.73E-07	1.15E-04	8	3.187251	51	0.28234512
GO:0019901	protein kinase binding	4.94E-07	1.19E-04	21	8.366534	422	2.3362675
GO:0043233	organelle lumen	5.23E-07	1.25E-04	82	32.669323	3532	19.553783
GO:0000022	mitotic spindle elongation	5.34E-07	1.26E-04	4	1.5936255	6	0.033217072
GO:0051231	spindle elongation	5.34E-07	1.26E-04	4	1.5936255	6	0.033217072
GO:0003774	motor activity	5.58E-07	1.30E-04	12	4.7808766	139	0.76952887
GO:0006461	protein complex assembly	6.26E-07	1.45E-04	33	13.14741	924	5.1154294
GO:0051784	negative regulation of nuclear division	6.42E-07	1.48E-04	8	3.187251	53	0.29341748
GO:1901991	negative regulation of mitotic cell cycle phase transition	6.64E-07	1.51E-04	13	5.1792827	168	0.9300781
GO:0032991	macromolecular complex	6.86E-07	1.55E-04	99	39.44223	4599	25.460886
GO:0070271	protein complex biogenesis	6.89E-07	1.55E-04	33	13.14741	928	5.137574
GO:1901988	negative regulation of cell cycle phase transition	7.59E-07	1.70E-04	13	5.1792827	170	0.9411504
GO:0034622	cellular macromolecular complex assembly	7.93E-07	1.76E-04	24	9.561753	550	3.0448985
GO:0045786	negative regulation of cell cycle	8.01E-07	1.76E-04	20	7.9681277	398	2.2033992
GO:1903051	negative regulation of proteolysis involved in cellular protein catabolic process	8.61E-07	1.88E-04	8	3.187251	55	0.30448985
GO:0031974	membrane-enclosed lumen	9.61E-07	2.03E-04	82	32.669323	3583	19.836128
GO:0045132	meiotic chromosome segregation	9.62E-07	2.03E-04	6	2.3904383	25	0.13840447
GO:0006336	DNA replication-independent nucleosome assembly	9.68E-07	2.03E-04	7	2.7888446	39	0.21591097
GO:0034080|GO:0034509	CENP-A containing nucleosome assembly	9.68E-07	2.03E-04	7	2.7888446	39	0.21591097
GO:0034724	DNA replication-independent nucleosome organization	9.68E-07	2.03E-04	7	2.7888446	39	0.21591097
GO:0061641	CENP-A containing chromatin organization	9.68E-07	2.03E-04	7	2.7888446	39	0.21591097
GO:1903363	negative regulation of cellular protein catabolic process	9.93E-07	2.06E-04	8	3.187251	56	0.31002602
GO:0070013	intracellular organelle lumen	1.02E-06	2.10E-04	80	31.87251	3468	19.199469
GO:0019886|GO:0042591|GO:0048005	antigen processing and presentation of exogenous peptide antigen via MHC class II	1.08E-06	2.21E-04	10	3.9840639	98	0.54254556
GO:0007093|GO:0031575|GO:0071780	mitotic cell cycle checkpoint	1.09E-06	2.23E-04	12	4.7808766	148	0.8193545
GO:0000228	nuclear chromosome	1.17E-06	2.36E-04	18	7.1713147	336	1.8601562
GO:0071241	cellular response to inorganic substance	1.18E-06	2.38E-04	10	3.9840639	99	0.5480817
GO:0002495	antigen processing and presentation of peptide antigen via MHC class II	1.30E-06	2.59E-04	10	3.9840639	100	0.5536179
GO:0031055	chromatin remodeling at centromere	1.38E-06	2.74E-04	7	2.7888446	41	0.22698334
GO:0002504	antigen processing and presentation of peptide or polysaccharide antigen via MHC class II	1.42E-06	2.80E-04	10	3.9840639	101	0.5591541
GO:0043231	intracellular membrane-bounded organelle	1.48E-06	2.87E-04	177	70.51793	10123	56.04274
GO:0043227	membrane-bounded organelle	1.48E-06	2.87E-04	178	70.916336	10200	56.469025
GO:0044464	cell part	1.62E-06	3.13E-04	233	92.82868	14914	82.566574
GO:0005623	cell	1.64E-06	3.14E-04	233	92.82868	14915	82.572105
GO:0032434	regulation of proteasomal ubiquitin-dependent protein catabolic process	1.71E-06	3.25E-04	10	3.9840639	103	0.57022643
GO:2001251	negative regulation of chromosome organization	2.03E-06	3.83E-04	9	3.5856574	82	0.45396668
GO:0051340	regulation of ligase activity	2.04E-06	3.83E-04	10	3.9840639	105	0.58129877
GO:0044446	intracellular organelle part	2.25E-06	4.21E-04	135	53.78486	7109	39.356697
GO:0045930	negative regulation of mitotic cell cycle	2.36E-06	4.39E-04	13	5.1792827	188	1.0408016
GO:0034501	protein localization to kinetochore	2.44E-06	4.51E-04	4	1.5936255	8	0.044289432
GO:0000079	regulation of cyclin-dependent protein serine/threonine kinase activity	2.49E-06	4.57E-04	9	3.5856574	84	0.46503904
GO:0005524	ATP binding	2.55E-06	4.65E-04	45	17.928288	1576	8.725018
GO:0019900	kinase binding	2.58E-06	4.69E-04	21	8.366534	468	2.5909317
GO:0007126	meiotic nuclear division	2.66E-06	4.76E-04	12	4.7808766	161	0.8913248
GO:0040001|GO:0018986|GO:0030605|GO:0030606|GO:0030608|GO:0030610	establishment of mitotic spindle localization	2.69E-06	4.76E-04	5	1.9920319	17	0.09411504
GO:0051310	metaphase plate congression	2.69E-06	4.76E-04	5	1.9920319	17	0.09411504
GO:0090307	spindle assembly involved in mitosis	2.69E-06	4.76E-04	5	1.9920319	17	0.09411504
GO:0043486	histone exchange	3.10E-06	5.47E-04	7	2.7888446	46	0.25466424
GO:0051321	meiotic cell cycle	3.66E-06	6.42E-04	12	4.7808766	166	0.9190057
GO:0071248	cellular response to metal ion	4.44E-06	7.73E-04	9	3.5856574	90	0.49825612
GO:0032559	adenyl ribonucleotide binding	4.47E-06	7.74E-04	45	17.928288	1610	8.913248
GO:0043623	cellular protein complex assembly	5.00E-06	8.55E-04	15	5.9760957	266	1.4726236
GO:0051640	organelle localization	5.00E-06	8.55E-04	15	5.9760957	266	1.4726236
GO:0061136	regulation of proteasomal protein catabolic process	5.04E-06	8.58E-04	10	3.9840639	116	0.6421968
GO:0044422	organelle part	5.24E-06	8.88E-04	136	54.183266	7277	40.286774
GO:0051656	establishment of organelle localization	5.80E-06	9.76E-04	13	5.1792827	204	1.1293805
GO:0030554	adenyl nucleotide binding	5.88E-06	9.84E-04	45	17.928288	1627	9.007363
GO:1902850	microtubule cytoskeleton organization involved in mitosis	6.51E-06	0.001083543	5	1.9920319	20	0.11072358
GO:0008608|GO:0051313	attachment of spindle microtubules to kinetochore	7.16E-06	0.001172378	4	1.5936255	10	0.05536179
GO:0051382|GO:0000069	kinetochore assembly	7.16E-06	0.001172378	4	1.5936255	10	0.05536179
GO:0060236	regulation of mitotic spindle organization	7.16E-06	0.001172378	4	1.5936255	10	0.05536179
GO:0043044	ATP-dependent chromatin remodeling	7.22E-06	0.001176585	7	2.7888446	52	0.2878813
GO:0051128	regulation of cellular component organization	7.78E-06	0.001259755	43	17.131474	1540	8.525716
GO:0043632	modification-dependent macromolecule catabolic process	8.14E-06	0.001311399	18	7.1713147	386	2.136965
GO:0018636	phenanthrene 9,10-monooxygenase activity	1.05E-05	0.001638997	3	1.1952192	4	0.022144716
GO:0032133	chromosome passenger complex	1.05E-05	0.001638997	3	1.1952192	4	0.022144716
GO:0036017	response to erythropoietin	1.05E-05	0.001638997	3	1.1952192	4	0.022144716
GO:0036018	cellular response to erythropoietin	1.05E-05	0.001638997	3	1.1952192	4	0.022144716
GO:0047026	androsterone dehydrogenase (A-specific) activity	1.05E-05	0.001638997	3	1.1952192	4	0.022144716
GO:0047086	ketosteroid monooxygenase activity	1.05E-05	0.001638997	3	1.1952192	4	0.022144716
GO:0000090	mitotic anaphase	1.11E-05	0.001728273	4	1.5936255	11	0.06089797
GO:0010639	negative regulation of organelle organization	1.13E-05	0.001747377	13	5.1792827	217	1.2013508
GO:0051438	regulation of ubiquitin-protein transferase activity	1.15E-05	0.001767135	9	3.5856574	101	0.5591541
GO:1903050	regulation of proteolysis involved in cellular protein catabolic process	1.21E-05	0.001852883	10	3.9840639	128	0.7086309
GO:1903046	meiotic cell cycle process	1.25E-05	0.001895607	9	3.5856574	102	0.56469023
GO:0044454	nuclear chromosome part	1.35E-05	0.002042688	15	5.9760957	289	1.5999557
GO:0051293	establishment of spindle localization	1.37E-05	0.002046682	5	1.9920319	23	0.12733212
GO:0072686	mitotic spindle	1.37E-05	0.002046682	5	1.9920319	23	0.12733212
GO:0045926	negative regulation of growth	1.37E-05	0.002050393	13	5.1792827	221	1.2234956
GO:1903362	regulation of cellular protein catabolic process	1.59E-05	0.002356548	10	3.9840639	132	0.7307756
GO:0090224	regulation of spindle organization	1.65E-05	0.002438434	4	1.5936255	12	0.066434145
GO:0065004	protein-DNA complex assembly	1.69E-05	0.002483906	11	4.38247	162	0.89686096
GO:0034502	protein localization to chromosome	1.70E-05	0.002494044	5	1.9920319	24	0.13286829
GO:0048015	phosphatidylinositol-mediated signaling	2.01E-05	0.002906887	11	4.38247	165	0.91346955
GO:0048017	inositol lipid-mediated signaling	2.01E-05	0.002906887	11	4.38247	165	0.91346955
GO:0044257	cellular protein catabolic process	2.02E-05	0.002916145	18	7.1713147	413	2.2864418
GO:0065003	macromolecular complex assembly	2.05E-05	0.002939745	33	13.14741	1089	6.0288987
GO:0006511	ubiquitin-dependent protein catabolic process	2.09E-05	0.002991266	17	6.772908	375	2.0760672
GO:0042177	negative regulation of protein catabolic process	2.18E-05	0.003100009	8	3.187251	84	0.46503904
GO:0045840	positive regulation of mitosis	2.34E-05	0.003275067	6	2.3904383	42	0.23251952
GO:0007091	metaphase/anaphase transition of mitotic cell cycle	2.36E-05	0.003275067	4	1.5936255	13	0.07197033
GO:0044784	metaphase/anaphase transition of cell cycle	2.36E-05	0.003275067	4	1.5936255	13	0.07197033
GO:0051322	anaphase	2.36E-05	0.003275067	4	1.5936255	13	0.07197033
GO:0071276	cellular response to cadmium ion	2.36E-05	0.003275067	4	1.5936255	13	0.07197033
GO:0051653	spindle localization	2.58E-05	0.003509761	5	1.9920319	26	0.14394066
GO:0009753	response to jasmonic acid	2.60E-05	0.003509761	3	1.1952192	5	0.027680894
GO:0047023	androsterone dehydrogenase activity	2.60E-05	0.003509761	3	1.1952192	5	0.027680894
GO:0047115	trans-1,2-dihydrobenzene-1,2-diol dehydrogenase activity	2.60E-05	0.003509761	3	1.1952192	5	0.027680894
GO:0051256	mitotic spindle midzone assembly	2.60E-05	0.003509761	3	1.1952192	5	0.027680894
GO:0071395	cellular response to jasmonic acid stimulus	2.60E-05	0.003509761	3	1.1952192	5	0.027680894

GO:0019941	modification-dependent protein catabolic process	2.65E-05	0.00356382	17	6.772908	382	2.1148202
GO:0044763	single-organism cellular process	2.75E-05	0.003683633	180	71.71315	10797	59.774124
GO:0000003|GO:0019952|GO:0050876	reproduction	2.86E-05	0.003808074	29	11.553785	912	5.048995
GO:0010043	response to zinc ion	3.08E-05	0.004082943	6	2.3904383	44	0.24359187
GO:0007076	mitotic chromosome condensation	3.27E-05	0.00427878	4	1.5936255	14	0.077506505
GO:0007080	mitotic metaphase plate congression	3.27E-05	0.00427878	4	1.5936255	14	0.077506505
GO:0008106	alcohol dehydrogenase (NADP+) activity	3.27E-05	0.00427878	4	1.5936255	14	0.077506505
GO:0051225|GO:0051226|GO:0051227	spindle assembly	3.51E-05	0.004576096	6	2.3904383	45	0.24912806
GO:0051603	proteolysis involved in cellular protein catabolic process	4.30E-05	0.005581111	17	6.772908	397	2.197863
GO:0040008	regulation of growth	4.75E-05	0.006148671	21	8.366534	568	3.1445496
GO:0000089	mitotic metaphase	5.14E-05	0.006536931	3	1.1952192	6	0.033217072
GO:0000796|GO:0005676|GO:0008620	condensin complex	5.14E-05	0.006536931	3	1.1952192	6	0.033217072
GO:0004958	prostaglandin F receptor activity	5.14E-05	0.006536931	3	1.1952192	6	0.033217072
GO:0051255	spindle midzone assembly	5.14E-05	0.006536931	3	1.1952192	6	0.033217072
GO:0071824	protein-DNA complex subunit organization	5.75E-05	0.007280374	11	4.38247	185	1.024193
GO:0001556	oocyte maturation	5.81E-05	0.007324421	4	1.5936255	16	0.088578865
GO:0009987|GO:0008151|GO:0050875	cellular process	6.64E-05	0.008340213	213	84.86056	13503	74.75503
GO:0030163	protein catabolic process	6.74E-05	0.008429957	18	7.1713147	453	2.507889
GO:0032467|GO:0071777	positive regulation of cytokinesis	7.51E-05	0.009356373	4	1.5936255	17	0.09411504
GO:0051338	regulation of transferase activity	7.79E-05	0.009654639	27	10.756972	867	4.799867
GO:0051785	positive regulation of nuclear division	8.08E-05	0.009984379	6	2.3904383	52	0.2878813
GO:0005814	centriole	8.17E-05	0.010049835	7	2.7888446	75	0.4152134
GO:0035639	purine ribonucleoside triphosphate binding	8.37E-05	0.010254169	47	18.7251	1923	10.646072
GO:0000166	nucleotide binding	8.77E-05	0.010694566	59	23.505976	2617	14.48818
GO:1901265	nucleoside phosphate binding	8.86E-05	0.01076676	59	23.505976	2618	14.493716
GO:0051488	activation of anaphase-promoting complex activity	8.90E-05	0.010776402	3	1.1952192	7	0.038753252
GO:0032550	purine ribonucleoside binding	9.49E-05	0.011433386	47	18.7251	1933	10.701434
GO:0000086	G2/M transition of mitotic cell cycle	9.62E-05	0.011502646	9	3.5856574	132	0.7307756
GO:0044839	cell cycle G2/M phase transition	9.62E-05	0.011502646	9	3.5856574	132	0.7307756
GO:0001883	purine nucleoside binding	9.85E-05	0.011726403	47	18.7251	1936	10.718042
GO:0032549	ribonucleoside binding	9.97E-05	0.011825791	47	18.7251	1937	10.723578
GO:0001882	nucleoside binding	1.13E-04	0.013322716	47	18.7251	1947	10.77894
GO:0051439	regulation of ubiquitin-protein ligase activity involved in mitotic cell cycle	1.14E-04	0.013400955	7	2.7888446	79	0.43735814
GO:0004033	aldo-keto reductase (NADP) activity	1.20E-04	0.014037667	4	1.5936255	19	0.1051874
GO:0036094	small molecule binding	1.30E-04	0.015176786	63	25.099602	2893	16.016165
GO:0032555	purine ribonucleotide binding	1.35E-04	0.015738616	47	18.7251	1962	10.861983
GO:0044444	cytoplasmic part	1.39E-04	0.01609618	131	52.191235	7351	40.696453
GO:0032052	bile acid binding	1.41E-04	0.016280526	3	1.1952192	8	0.044289432
GO:0006334	nucleosome assembly	1.43E-04	0.016323986	9	3.5856574	139	0.76952887
GO:0000082	G1/S transition of mitotic cell cycle	1.43E-04	0.016323986	10	3.9840639	171	0.9466866
GO:0044843	cell cycle G1/S phase transition	1.43E-04	0.016323986	10	3.9840639	171	0.9466866
GO:0032553	ribonucleotide binding	1.64E-04	0.018622303	47	18.7251	1978	10.950562
GO:0048519|GO:0043118	negative regulation of biological process	1.66E-04	0.018757928	77	30.67729	3780	20.926756
GO:1990267	response to transition metal nanoparticle	1.70E-04	0.01923452	8	3.187251	112	0.62005204
GO:0017076	purine nucleotide binding	1.72E-04	0.019246124	47	18.7251	1982	10.972707
GO:0046686	response to cadmium ion	1.72E-04	0.019246124	5	1.9920319	38	0.2103748
GO:0016538|GO:0003751|GO:0003752|GO:0003753	activity	1.81E-04	0.02007943	4	1.5936255	21	0.11625976
GO:0002478	antigen processing and presentation of exogenous peptide antigen	1.81E-04	0.02007943	10	3.9840639	176	0.9743675
GO:0044450	microtubule organizing center part	1.81E-04	0.02007943	8	3.187251	113	0.62558824
GO:0004808|GO:0016425	tRNA (5-methylaminomethyl-2-thiouridylate)-methyltransferase activity	1.92E-04	0.020923488	2	0.7968128	2	0.011072358
GO:0007079|GO:0007082	mitotic chromosome movement towards spindle pole	1.92E-04	0.020923488	2	0.7968128	2	0.011072358
GO:0010993	regulation of ubiquitin homeostasis	1.92E-04	0.020923488	2	0.7968128	2	0.011072358
GO:0010994	free ubiquitin chain polymerization	1.92E-04	0.020923488	2	0.7968128	2	0.011072358
GO:0030892	mitotic cohesin complex	1.92E-04	0.020923488	2	0.7968128	2	0.011072358
GO:0004032	alditol:NADP+ 1-oxidoreductase activity	2.09E-04	0.022291152	3	1.1952192	9	0.04982561
GO:0030647	aminoglycoside antibiotic metabolic process	2.09E-04	0.022291152	3	1.1952192	9	0.04982561
GO:0044597	daunorubicin metabolic process	2.09E-04	0.022291152	3	1.1952192	9	0.04982561
GO:0044598	doxorubicin metabolic process	2.09E-04	0.022291152	3	1.1952192	9	0.04982561
GO:0051323	metaphase	2.09E-04	0.022291152	3	1.1952192	9	0.04982561
GO:0051984	positive regulation of chromosome segregation	2.09E-04	0.022291152	3	1.1952192	9	0.04982561
GO:0050790	regulation of catalytic activity	2.10E-04	0.022312803	49	19.521912	2114	11.703483
GO:0005680	anaphase-promoting complex	2.19E-04	0.023038581	4	1.5936255	22	0.12179594
GO:0050685	positive regulation of mRNA processing	2.19E-04	0.023038581	4	1.5936255	22	0.12179594
GO:0065009	regulation of molecular function	2.27E-04	0.02378776	56	22.310757	2530	14.006533
GO:0004674|GO:0004695|GO:0004696|GO:0004700	protein serine/threonine kinase activity	2.35E-04	0.024597153	17	6.772908	457	2.5300338
GO:0051129	negative regulation of cellular component organization	2.41E-04	0.025093447	16	6.374502	415	2.2975142
GO:0042176	regulation of protein catabolic process	2.46E-04	0.025512319	11	4.38247	218	1.206887
GO:0019884	antigen processing and presentation of exogenous antigen	2.48E-04	0.025702547	10	3.9840639	183	1.0131208
GO:0031497	chromatin assembly	2.66E-04	0.027481396	9	3.5856574	151	0.835963
GO:0051351	positive regulation of ligase activity	2.76E-04	0.02839457	7	2.7888446	91	0.5037923
GO:0000085	mitotic G2 phase	2.96E-04	0.030133594	3	1.1952192	10	0.05536179
GO:0010369	chromocenter	2.96E-04	0.030133594	3	1.1952192	10	0.05536179
GO:0051319	G2 phase	2.96E-04	0.030133594	3	1.1952192	10	0.05536179
GO:0031330	negative regulation of cellular catabolic process	3.61E-04	0.036654305	8	3.187251	125	0.6920224
GO:0061640	cytoskeleton-dependent cytokinesis	3.66E-04	0.037005283	4	1.5936255	25	0.13840447
GO:0097367	carbohydrate derivative binding	3.68E-04	0.037088484	51	20.318726	2282	12.63356
GO:0019899	enzyme binding	3.83E-04	0.038501494	33	13.14741	1271	7.0364833
GO:0048002	antigen processing and presentation of peptide antigen	3.95E-04	0.039581697	10	3.9840639	194	1.0740187
GO:0030638	polyketide metabolic process	4.03E-04	0.040194172	3	1.1952192	11	0.06089797
GO:0034453	microtubule anchoring	4.28E-04	0.04226644	4	1.5936255	26	0.14394066
GO:1901661|GO:0042375	quinone metabolic process	4.28E-04	0.04226644	4	1.5936255	26	0.14394066
GO:1903313	positive regulation of mRNA metabolic process	4.28E-04	0.04226644	4	1.5936255	26	0.14394066
GO:0034728	nucleosome organization	4.47E-04	0.04402107	9	3.5856574	162	0.89686096
GO:0070925	organelle assembly	4.72E-04	0.04633833	13	5.1792827	314	1.7383602
GO:0031461	cullin-RING ubiquitin ligase complex	5.54E-04	0.054172784	7	2.7888446	102	0.56469023
GO:0000942	condensed nuclear chromosome outer kinetochore	5.72E-04	0.055216696	2	0.7968128	3	0.016608536
GO:0045726	positive regulation of integrin biosynthetic process	5.72E-04	0.055216696	2	0.7968128	3	0.016608536
GO:0051305	chromosome movement towards spindle pole	5.72E-04	0.055216696	2	0.7968128	3	0.016608536
GO:0097149	centralspindlin complex	5.72E-04	0.055216696	2	0.7968128	3	0.016608536
GO:0031110	regulation of microtubule polymerization or depolymerization	5.76E-04	0.05550054	5	1.9920319	49	0.27127278
GO:0006333	chromatin assembly or disassembly	6.34E-04	0.060816843	9	3.5856574	170	0.9411504
GO:0051493	regulation of cytoskeleton organization	6.51E-04	0.062321823	13	5.1792827	325	1.7992581
GO:0008283	cell proliferation	6.62E-04	0.06308207	20	7.9681277	641	3.5486908
GO:0004955	prostaglandin receptor activity	6.84E-04	0.064816184	3	1.1952192	13	0.07197033
GO:0007096	regulation of exit from mitosis	6.84E-04	0.064816184	3	1.1952192	13	0.07197033
GO:0005654	nucleoplasm	7.10E-04	0.06708551	36	14.342629	1482	8.2046175
GO:0048523|GO:0051243	negative regulation of cellular process	7.24E-04	0.06815715	69	27.49004	3452	19.11089
GO:0090304	nucleic acid metabolic process	7.47E-04	0.06999865	79	31.474104	4092	22.654045
GO:0070613|GO:0010953	regulation of protein processing	7.50E-04	0.06999865	13	5.1792827	330	1.8269391
GO:1903317	regulation of protein maturation	7.50E-04	0.06999865	13	5.1792827	330	1.8269391
GO:0008092	cytoskeletal protein binding	7.72E-04	0.07175454	22	8.76494	747	4.1355257
GO:0022607|GO:0071844	cellular component assembly	7.82E-04	0.0724902	38	15.139442	1602	8.868958
GO:0045861	negative regulation of proteolysis	8.51E-04	0.07833656	12	4.7808766	293	1.6221005
GO:0032465|GO:0071775	regulation of cytokinesis	8.53E-04	0.07833656	4	1.5936255	31	0.17162155
GO:0048599	oocyte development	8.53E-04	0.07833656	4	1.5936255	31	0.17162155
GO:0004954	prostanoid receptor activity	8.62E-04	0.078919396	3	1.1952192	14	0.077506505
GO:0022412	cellular process involved in reproduction in multicellular organism	9.76E-04	0.08915096	10	3.9840639	218	1.206887
GO:0008301	DNA binding, bending	9.83E-04	0.0894913	5	1.9920319	55	0.30448985
GO:0019882|GO:0030333	antigen processing and presentation	0.001046281	0.09496554	10	3.9840639	220	1.2179594
GO:0005730	nucleolus	0.001053846	0.09536493	39	15.537848	1685	9.328462
GO:0000132|GO:0030607|GO:0030609	establishment of mitotic spindle orientation	0.001066033	0.09572034	3	1.1952192	15	0.08304268
GO:0016137|GO:0016140	glycoside metabolic process	0.001066033	0.09572034	3	1.1952192	15	0.08304268
GO:0048477|GO:0009993|GO:0048157	oogenesis	0.001067303	0.09572034	5	1.9920319	56	0.31002602
GO:0010955	negative regulation of protein processing	0.001073916	0.095743574	12	4.7808766	301	1.6663898
GO:1903318	negative regulation of protein maturation	0.001073916	0.095743574	12	4.7808766	301	1.6663898
GO:0009994	oocyte differentiation	0.001085045	0.09645039	4	1.5936255	33	0.1826939

GO:0065007	biological regulation	0.001125691	0.09866732	164	65.338646	10064	55.716106
GO:0000212	meiotic spindle organization	0.001132905	0.09866732	2	0.7968128	4	0.022144716
GO:0031536	positive regulation of exit from mitosis	0.001132905	0.09866732	2	0.7968128	4	0.022144716
GO:0044530	supraspliceosomal complex	0.001132905	0.09866732	2	0.7968128	4	0.022144716
GO:0045113	regulation of integrin biosynthetic process	0.001132905	0.09866732	2	0.7968128	4	0.022144716
GO:0051299	centrosome separation	0.001132905	0.09866732	2	0.7968128	4	0.022144716
GO:0060564	negative regulation of mitotic anaphase-promoting complex activity	0.001132905	0.09866732	2	0.7968128	4	0.022144716

Table S8 GO HUV 12hr UP

GO ACCESSION	GO Term	p-value	corrected p-value	Count in Selection	% Count in Selection	Count in Total	% Count in Total
GO:0007067	mitotic nuclear division	2.28E-28	8.26E-24	44	15.224914	318	1.7605048
GO:0000278	mitotic cell cycle	1.22E-26	1.99E-22	61	21.107267	738	4.0857
GO:1903047	mitotic cell cycle process	1.65E-26	1.99E-22	58	20.069204	665	3.681559
GO:0000280	nuclear division	1.53E-25	1.39E-21	48	16.608997	455	2.5189614
GO:0048285	organelle fission	2.20E-25	1.60E-21	49	16.955017	481	2.662902
GO:0051301	cell division	2.87E-23	1.74E-19	46	15.916955	466	2.5798595
GO:0022402	cell cycle process	1.67E-22	8.67E-19	65	22.49135	1004	5.558324
GO:0000087	mitotic M phase	1.29E-19	5.21E-16	23	7.9584775	108	0.5979073
GO:0007049	cell cycle	1.27E-19	5.21E-16	70	24.221453	1307	7.235786
GO:0000279	M phase	4.22E-17	1.53E-13	23	7.9584775	138	0.76399267
GO:0000777	condensed chromosome kinetochore	3.06E-16	1.01E-12	19	6.574394	91	0.5037923
GO:0000779	condensed chromosome, centromeric region	7.17E-16	2.16E-12	19	6.574394	95	0.525937
GO:0000793	condensed chromosome	6.46E-15	1.80E-11	24	8.304499	191	1.0574101
GO:0022403	cell cycle phase	1.19E-14	3.07E-11	29	10.034602	302	1.671926
GO:0005819	spindle	1.99E-14	4.80E-11	27	9.342561	263	1.4560151
GO:0000776|GO:0005699	kinetochore	2.45E-14	5.55E-11	19	6.574394	114	0.6311244
GO:0000236	mitotic prometaphase	3.05E-14	6.50E-11	17	5.882353	86	0.47611138
GO:0000775|GO:0097521	chromosome, centromeric region	3.78E-14	7.61E-11	22	7.612457	168	0.9300781
GO:1902589	single-organism organelle organization	4.15E-14	7.92E-11	69	23.875433	1627	9.007363
GO:0007059	chromosome segregation	6.46E-14	1.17E-10	21	7.266436	154	0.85257155
GO:0044848	biological phase	1.17E-13	2.03E-10	29	10.034602	330	1.8269391
GO:0098687	chromosomal region	1.51E-12	2.49E-09	23	7.9584775	222	1.2290317
GO:0007017	microtubule-based process	3.99E-12	6.29E-09	31	10.726644	434	2.4027016
GO:0010564	regulation of cell cycle process	1.83E-11	2.76E-08	29	10.034602	404	2.2366164
GO:0000226	microtubule cytoskeleton organization	2.27E-11	3.30E-08	24	8.304499	277	1.5335215
GO:0006996	organelle organization	9.52E-11	1.33E-07	81	28.027681	2456	13.596855
GO:0051302	regulation of cell division	5.17E-10	6.94E-07	20	6.9204154	221	1.2234956
GO:0007088	regulation of mitosis	5.71E-10	7.38E-07	15	5.1903114	116	0.6421968
GO:0051983	regulation of chromosome segregation	6.64E-10	8.29E-07	12	4.1522493	67	0.370924
GO:0007051	spindle organization	7.28E-10	8.79E-07	13	4.49827	83	0.45950285
GO:0007346	regulation of mitotic cell cycle	1.20E-09	1.41E-06	24	8.304499	336	1.8601562
GO:0071294	cellular response to zinc ion	3.30E-09	3.74E-06	6	2.0761247	10	0.05536179
GO:0000819	sister chromatid segregation	3.85E-09	4.23E-06	11	3.8062284	62	0.3432431
GO:0005634	nucleus	4.84E-09	5.16E-06	151	52.249134	6394	35.398327
GO:0015630	microtubule cytoskeleton	5.40E-09	5.59E-06	42	14.532872	974	5.392238
GO:0051783	regulation of nuclear division	6.57E-09	6.62E-06	15	5.1903114	138	0.76399267
GO:0005874	microtubule	1.28E-08	1.26E-05	24	8.304499	379	2.0982118
GO:0045931	positive regulation of mitotic cell cycle	1.68E-08	1.60E-05	12	4.1522493	88	0.48718375
GO:0000778	condensed nuclear chromosome kinetochore	2.15E-08	1.99E-05	5	1.7301039	7	0.038753252
GO:0005694	chromosome	2.75E-08	2.49E-05	34	11.764706	729	4.0358744
GO:0000922|GO:0030615	spindle pole	3.08E-08	2.72E-05	13	4.49827	112	0.62005204
GO:0000780	condensed nuclear chromosome, centromeric region	4.47E-08	3.86E-05	6	2.0761247	14	0.077506505
GO:0051726|GO:0000074	regulation of cell cycle	5.12E-08	4.31E-05	34	11.764706	748	4.141062
GO:0007052	mitotic spindle organization	6.65E-08	5.35E-05	8	2.768166	35	0.19376627
GO:0031262	Ndc80 complex	6.60E-08	5.35E-05	4	1.384083	4	0.022144716
GO:0045787	positive regulation of cell cycle	9.29E-08	7.32E-05	18	6.2283735	244	1.3508277
GO:0044427	chromosomal part	1.10E-07	8.51E-05	30	10.380623	627	3.4711843
GO:0031981	nuclear lumen	1.21E-07	9.12E-05	81	28.027681	2862	15.844544
GO:0031577	spindle checkpoint	2.03E-07	1.50E-04	8	2.768166	40	0.22144715
GO:0000070|GO:0016359	mitotic sister chromatid segregation	2.15E-07	1.56E-04	9	3.1141868	55	0.30448985
GO:0090068	positive regulation of cell cycle process	3.57E-07	2.54E-04	15	5.1903114	186	1.0297292
GO:0051276|GO:0007001|GO:0051277	chromosome organization	4.23E-07	2.95E-04	33	11.418685	780	4.3182197
GO:0005876	spindle microtubule	4.42E-07	3.02E-04	8	2.768166	44	0.24359187
GO:0007010	cytoskeleton organization	1.15E-06	7.72E-04	32	11.072664	777	4.301611
GO:0071276	cellular response to cadmium ion	1.21E-06	7.86E-04	5	1.7301039	13	0.07197033
GO:0000940	condensed chromosome outer kinetochore	1.21E-06	7.86E-04	5	1.7301039	13	0.07197033
GO:1901990	regulation of mitotic cell cycle phase transition	1.31E-06	8.34E-04	15	5.1903114	206	1.1404529
GO:0033043	regulation of organelle organization	1.38E-06	8.59E-04	29	10.034602	669	3.7037036
GO:1901987	regulation of cell cycle phase transition	1.57E-06	9.66E-04	15	5.1903114	209	1.1570613
GO:0030496	midbody	1.63E-06	9.68E-04	11	3.8062284	110	0.6089797
GO:0051781	positive regulation of cell division	1.63E-06	9.68E-04	11	3.8062284	110	0.6089797
GO:0070013	intracellular organelle lumen	1.85E-06	0.001081263	89	30.795847	3468	19.199469
GO:0046686	response to cadmium ion	2.16E-06	0.001201889	7	2.4221454	38	0.2103748
GO:0031974	membrane-enclosed lumen	2.10E-06	0.001201889	91	31.487888	3583	19.836128
GO:0043233	organelle lumen	2.14E-06	0.001201889	90	31.141869	3532	19.553783
GO:0000075|GO:0031576|GO:0071779	cell cycle checkpoint	2.19E-06	0.001203422	16	5.536332	243	1.3452915
GO:0006323	DNA packaging	2.28E-06	0.001232489	14	4.8442907	188	1.0408016
GO:0008017	microtubule binding	2.46E-06	0.001310056	13	4.49827	163	0.90239716
GO:0032886	regulation of microtubule-based process	2.53E-06	0.001330376	11	3.8062284	115	0.6366606
GO:0070507	regulation of microtubule cytoskeleton organization	3.69E-06	0.001910435	10	3.4602077	97	0.53700936
GO:0043232	intracellular non-membrane-bounded organelle	3.81E-06	0.001916541	100	34.602077	4114	22.77584
GO:0043228	non-membrane-bounded organelle	3.81E-06	0.001916541	100	34.602077	4114	22.77584
GO:0044430	cytoskeletal part	4.11E-06	0.002040889	43	14.878893	1288	7.1305985
GO:0051988	regulation of attachment of spindle microtubules to kinetochore	4.39E-06	0.00209302	4	1.384083	8	0.044289432
GO:0015631	tubulin binding	4.37E-06	0.00209302	15	5.1903114	227	1.2567127
GO:0045840	positive regulation of mitosis	4.36E-06	0.00209302	7	2.4221454	42	0.23251952
GO:0040001|GO:0018986|GO:0030605|GO:0030606|GO:0030608|GO:0030610	establishment of mitotic spindle localization	5.54E-06	0.002571776	5	1.7301039	17	0.09411504
GO:0000794	condensed nuclear chromosome	5.51E-06	0.002571776	9	3.1141868	80	0.4428943
GO:0044428	nuclear part	5.86E-06	0.002686202	83	28.719723	3249	17.987045
GO:1902099	regulation of metaphase/anaphase transition of cell cycle	6.03E-06	0.002696292	7	2.4221454	44	0.24359187
GO:0030071	regulation of mitotic metaphase/anaphase transition	6.03E-06	0.002696292	7	2.4221454	44	0.24359187
GO:0005737	cytoplasm	7.69E-06	0.003397795	194	67.12803	9839	54.470463
GO:0034508	centromere complex assembly	1.10E-05	0.00478507	7	2.4221454	48	0.26573658
GO:0010639	negative regulation of organelle organization	1.20E-05	0.005168879	14	4.8442907	217	1.2013508
GO:0033047	regulation of mitotic sister chromatid segregation	1.26E-05	0.005253166	7	2.4221454	49	0.27127278
GO:0010965	regulation of mitotic sister chromatid separation	1.26E-05	0.005253166	7	2.4221454	49	0.27127278
GO:0033045	regulation of sister chromatid segregation	1.26E-05	0.005253166	7	2.4221454	49	0.27127278
GO:0071459	protein localization to chromosome, centromeric region	1.28E-05	0.005286112	4	1.384083	10	0.05536179
GO:0071248	cellular response to metal ion	1.46E-05	0.005933027	9	3.1141868	90	0.49825612
GO:0047026	androsterone dehydrogenase (A-specific) activity	1.64E-05	0.006305683	3	1.0380623	4	0.022144716
GO:0036017	response to erythropoietin	1.64E-05	0.006305683	3	1.0380623	4	0.022144716
GO:0047086	ketosteroid monooxygenase activity	1.64E-05	0.006305683	3	1.0380623	4	0.022144716
GO:0036018	cellular response to erythropoietin	1.64E-05	0.006305683	3	1.0380623	4	0.022144716
GO:0018636	phenanthrene 9,10-monooxygenase activity	1.64E-05	0.006305683	3	1.0380623	4	0.022144716
GO:0016043|GO:0044235|GO:0071842	cellular component organization	1.69E-05	0.006390582	103	35.640137	4418	24.45884
GO:0008284	positive regulation of cell proliferation	1.69E-05	0.006390582	28	9.688581	722	3.997121
GO:0051303	establishment of chromosome localization	1.73E-05	0.006450236	5	1.7301039	21	0.11625976
GO:0071173	spindle assembly checkpoint	1.82E-05	0.006726083	6	2.0761247	35	0.19376627
GO:0071840|GO:0071841	cellular component organization or biogenesis	1.88E-05	0.00683029	105	36.33218	4539	25.128716
GO:0051785	positive regulation of nuclear division	1.88E-05	0.00683029	7	2.4221454	52	0.2878813
GO:0050000	chromosome localization	2.21E-05	0.00791155	5	1.7301039	22	0.12179594
GO:0005871	kinesin complex	2.43E-05	0.00862419	7	2.4221454	54	0.29895365
GO:0071174	mitotic spindle checkpoint	2.54E-05	0.008921314	6	2.0761247	37	0.20483862
GO:0051293	establishment of spindle localization	2.78E-05	0.009688773	5	1.7301039	23	0.12733212
GO:0007093|GO:0031575|GO:0071780	mitotic cell cycle checkpoint	2.85E-05	0.009821313	11	3.8062284	148	0.8193545
GO:0051985	negative regulation of chromosome segregation	2.97E-05	0.01015604	6	2.0761247	38	0.2103748
GO:0071241	cellular response to inorganic substance	3.15E-05	0.010661226	9	3.1141868	99	0.5480817
GO:0009753	response to jasmonic acid	4.04E-05	0.013190324	3	1.0380623	5	0.027680894
GO:0047115	trans-1,2-dihydrobenzene-1,2-diol dehydrogenase activity	4.04E-05	0.013190324	3	1.0380623	5	0.027680894
GO:0047023	androsterone dehydrogenase activity	4.04E-05	0.013190324	3	1.0380623	5	0.027680894
GO:0071395	cellular response to jasmonic acid stimulus	4.04E-05	0.013190324	3	1.0380623	5	0.027680894
GO:0044424	intracellular part	4.55E-05	0.014705904	235	81.31488	12822	70.984886
GO:0051653	spindle localization	5.23E-05	0.01675519	5	1.7301039	26	0.14394066
GO:0071822	protein complex subunit organization	5.35E-05	0.016853418	40	13.840831	1294	7.1638155
GO:0010948	negative regulation of cell cycle process	5.31E-05	0.016853418	13	4.49827	217	1.2013508
GO:0005856	cytoskeleton	5.67E-05	0.017685182	51	17.647058	1815	10.048164
GO:0003777	microtubule motor activity	5.71E-05	0.017685182	8	2.768166	83	0.45950285
GO:0008106	alcohol dehydrogenase (NADP+) activity	5.82E-05	0.017856944	4	1.384083	14	0.077506505
GO:0048519|GO:0043118	negative regulation of biological process	6.09E-05	0.018551169	89	30.795847	3780	20.926756
GO:0065004	protein-DNA complex assembly	6.52E-05	0.01969473	11	3.8062284	162	0.89686096
GO:0010043	response to zinc ion	7.01E-05	0.020979328	6	2.0761247	44	0.24359187
GO:0048017	inositol lipid-mediated signaling	7.70E-05	0.022316074	11	3.8062284	165	0.91346955

GO:0006692	prostanoid metabolic process	7.60E-05	0.022316074	5	1.7301039	28	0.15501301
GO:0006693	prostaglandin metabolic process	7.60E-05	0.022316074	5	1.7301039	28	0.15501301
GO:0048015	phosphatidylinositol-mediated signaling	7.70E-05	0.022316074	11	3.8062284	165	0.91346955
GO:0000910|GO:0007104|GO:0016288|GO:0033205	cytokinesis	7.77E-05	0.022339545	9	3.1141868	111	0.61451584
GO:0000022	mitotic spindle elongation	7.98E-05	0.022428554	3	1.0380623	6	0.033217072
GO:0051231	spindle elongation	7.98E-05	0.022428554	3	1.0380623	6	0.033217072
GO:0004958	prostaglandin F receptor activity	7.98E-05	0.022428554	3	1.0380623	6	0.033217072
GO:1990267	response to transition metal nanoparticle	8.33E-05	0.023216933	9	3.1141868	112	0.62005204
GO:1901991	negative regulation of mitotic cell cycle phase transition	9.05E-05	0.025036655	11	3.8062284	168	0.9300781
GO:0031145	anaphase-promoting complex-dependent proteasomal ubiquitin-dependent protein catabolic process	9.41E-05	0.025826272	8	2.768166	89	0.49271992
GO:1901988	negative regulation of cell cycle phase transition	1.01E-04	0.027414154	11	3.8062284	170	0.9411504
GO:0061136	regulation of proteasomal protein catabolic process	1.09E-04	0.029550746	9	3.1141868	116	0.6421968
GO:0005524	ATP binding	1.15E-04	0.030850817	45	15.570934	1576	8.725018
GO:0005875	microtubule associated complex	1.16E-04	0.03090578	10	3.4602077	144	0.79720974
GO:0030261|GO:0000068	chromosome condensation	1.26E-04	0.03342077	5	1.7301039	31	0.17162155
GO:0007019	microtubule depolymerization	1.38E-04	0.036252372	3	1.0380623	7	0.038753252
GO:0019899	enzyme binding	1.66E-04	0.043315932	38	13.148788	1271	7.0364833
GO:0019901	protein kinase binding	1.79E-04	0.046455722	18	6.2283735	422	2.3362675
GO:0071103	DNA conformation change	1.83E-04	0.047067996	14	4.8442907	279	1.5445939
GO:0032559	adenyl ribonucleotide binding	1.87E-04	0.0476482	45	15.570934	1610	8.913248
GO:0010038	response to metal ion	1.90E-04	0.04815851	14	4.8442907	280	1.5501301
GO:0007094	mitotic spindle assembly checkpoint	1.99E-04	0.050055504	5	1.7301039	34	0.18823008
GO:0005622	intracellular	2.05E-04	0.051223937	237	82.00692	13150	72.80075
GO:0004033	aldo-keto reductase (NADP) activity	2.11E-04	0.05225502	4	1.384083	19	0.1051874
GO:0071824	protein-DNA complex subunit organization	2.12E-04	0.05225502	11	3.8062284	185	1.024193
GO:0034501	protein localization to kinetochore	2.18E-04	0.053081088	3	1.0380623	8	0.044289432
GO:0042989	sequestering of actin monomers	2.18E-04	0.053081088	3	1.0380623	8	0.044289432
GO:1903050	regulation of proteolysis involved in cellular protein catabolic process	2.31E-04	0.055819012	9	3.1141868	128	0.7086309
GO:0044763	single-organism cellular process	2.36E-04	0.05591843	202	69.896194	10797	59.774124
GO:0005654	nucleoplasm	2.34E-04	0.05591843	42	14.532872	1482	8.2046175
GO:0030554	adenyl nucleotide binding	2.36E-04	0.05591843	45	15.570934	1627	9.007363
GO:0045930	negative regulation of mitotic cell cycle	2.44E-04	0.05734426	11	3.8062284	188	1.0408016
GO:0070542	response to fatty acid	2.48E-04	0.057994843	6	2.0761247	55	0.30448985
GO:0033046	negative regulation of sister chromatid segregation	2.63E-04	0.058717426	5	1.7301039	36	0.19930243
GO:0032434	regulation of proteasomal ubiquitin-dependent protein catabolic process	2.61E-04	0.058717426	8	2.768166	103	0.57022643
GO:0007079|GO:0007082	mitotic chromosome movement towards spindle pole	2.59E-04	0.058717426	2	0.6920415	2	0.011072358
GO:2000816	negative regulation of mitotic sister chromatid separation	2.63E-04	0.058717426	5	1.7301039	36	0.19930243
GO:1902100	negative regulation of metaphase/anaphase transition of cell cycle	2.63E-04	0.058717426	5	1.7301039	36	0.19930243
GO:0045841	negative regulation of mitotic metaphase/anaphase transition	2.63E-04	0.058717426	5	1.7301039	36	0.19930243
GO:0033048	negative regulation of mitotic sister chromatid segregation	2.63E-04	0.058717426	5	1.7301039	36	0.19930243
GO:1903362	regulation of cellular protein catabolic process	2.91E-04	0.06465252	9	3.1141868	132	0.7307756
GO:0051340	regulation of ligase activity	2.98E-04	0.06575704	8	2.768166	105	0.58129877
GO:0051984	positive regulation of chromosome segregation	3.24E-04	0.069362596	3	1.0380623	9	0.04982561
GO:0044597	daunorubicin metabolic process	3.24E-04	0.069362596	3	1.0380623	9	0.04982561
GO:0044598	doxorubicin metabolic process	3.24E-04	0.069362596	3	1.0380623	9	0.04982561
GO:0004032	alditol:NADP+ 1-oxidoreductase activity	3.24E-04	0.069362596	3	1.0380623	9	0.04982561
GO:0030647	aminoglycoside antibiotic metabolic process	3.24E-04	0.069362596	3	1.0380623	9	0.04982561
GO:0010035	response to inorganic substance	3.45E-04	0.07347951	17	5.882353	407	2.2532248
GO:0051782	negative regulation of cell division	4.38E-04	0.09288711	6	2.0761247	61	0.33770692
GO:0006259|GO:0055132	DNA metabolic process	4.61E-04	0.09540878	29	10.034602	922	5.104357
GO:0072686	mitotic spindle	4.59E-04	0.09540878	4	1.384083	23	0.12733212
GO:0051382|GO:0000069	kinetochore assembly	4.57E-04	0.09540878	3	1.0380623	10	0.05536179
GO:0060236	regulation of mitotic spindle organization	4.57E-04	0.09540878	3	1.0380623	10	0.05536179

Table S9 Pathway analysis through WikiPathways in the fibroblast irradiated with UV-C (downregulation, 4hr, 0.5J/m^2)	p-value (low dose 4hr)	Matched Entities (low dose 4hr)	Pathway Entities of Experiment Type (low dose 4hr)
Hs_Circadian_Clock_WP1797_76871	2.50E-04	4	49
Hs_Regulation_of_Lipid_Metabolism_by_Peroxisome_proliferator-activated_receptor_alpha_(PPARalpha)_WP2797_77088	7.32E-04	5	118
Hs_Pre-NOTCH_Expression_and_Processing_WP2786_77076	0.001236412	3	38
Hs_Diurnally_Regulated_Genes_with_Circadian_Orthologs_WP410_69903	0.003351741	3	48
Hs_miR-targeted_genes_in_muscle_cell_-_TarBase_WP2005_78538	0.005197532	7	409
Hs_Insulin_Processing_WP2736_76967	0.006804433	2	20
Hs_miR-targeted_genes_in_epithelium_-_TarBase_WP2002_78530	0.007813588	6	345
Hs_Mesodermal_Commitment_Pathway_WP2857_78577	0.008866091	3	154
Hs_Transcriptional_Regulation_of_White_Adipocyte_Differentiation_WP2751_76992	0.010756369	3	74
Hs_Arylhydrocarbon_receptor_(AhR)_signaling_pathway_WP2100_74081	0.012215915	2	28
Hs_Apoptosis_Modulation_and_Signaling_WP1772_63162	0.013765656	3	93
Hs_Nuclear_Receptors_Meta-Pathway_WP2882_78569	0.014100108	6	318
Hs_MicroRNAs_in_cardiomyocyte_hypertrophy_WP1544_75258	0.01567848	3	104
Hs_TNF_alpha_Signaling_Pathway_WP2808_78568	0.016688107	3	87
Hs_Phase_1_-_Functionalization_of_compounds_WP1879_76834	0.01793261	2	33
Hs_TNF_alpha_Signaling_Pathway_WP231_79280	0.018269373	3	90
Hs_Corticotropin-releasing_hormone_WP2355_78490	0.018814325	3	90
Hs_Corticotropin-releasing_hormone_WP2355_79562	0.019368224	3	91
Hs_Nuclear_Receptors_WP170_71083	0.023396417	2	38
Hs_Neural_Crest_Differentiation_WP2064_79263	0.024121908	3	101
Hs_TWEAK_Signaling_Pathway_WP2036_78525	0.026960611	2	41
Hs_Transcriptional_activity_of_SMAD2-SMAD3-SMAD4_heterotrimer_WP2755_77005	0.028194314	2	44
Hs_Sulindac_Metabolic_Pathway_WP2542_70621	0.030714499	1	5
Hs_Aryl_Hydrocarbon_Receptor_Pathway_WP2873_79696	0.032027762	2	46
Hs_Aryl_Hydrocarbon_Receptor_pathway_WP2873_79544	0.032027762	2	46
Hs_Aryl_Hydrocarbon_Receptor_WP2586_78547	0.03334863	2	47
Hs_Notch_Signaling_Pathway_WP268_70096	0.03334863	2	46
Hs_Translocation_of_GLUT4_to_the_Plasma_Membrane_WP2777_77058	0.040261786	2	51
Hs_Cardiac_Hypertrophic_Response_WP2795_78544	0.04464583	2	54
Hs_miR-targeted_genes_in_lymphocytes_-_TarBase_WP2004_78524	0.044664748	6	495
Hs_Adipogenesis_WP236_78584	0.047899272	3	131
Hs_Oxidation_by_Cytochrome_P450_WP43_73490	0.05390846	2	63
Hs_Benzo(a)pyrene_metabolism_WP696_72081	0.05460751	1	9
Hs_Notch_Signaling_Pathway_WP61_78592	0.055513054	2	61
Hs_Oxidation_by_Cytochrome_P450_WP43_79585	0.055513054	2	63
Hs_Semaphorin_interactions_WP1907_76850	0.057134297	2	62
Hs_Endochondral_Ossification_WP474_72122	0.060425658	2	64
Hs_Mammary_gland_development_pathway_-_Involution_(Stage_4_of_4)_WP2815_78062	0.060488448	1	10
Hs_Vitamin_D_Metabolism_WP1531_74057	0.066332914	1	11
Hs_DNA_Damage_Response_WP707_78527	0.067196555	2	68
Hs_Endoderm_Differentiation_WP2853_78496	0.067196555	2	146
Hs_Glucocorticoid_Receptor_Pathway_WP2880_79522	0.070672	2	71
Hs_Glucocorticoid_Receptor_Pathway_WP2880_79615	0.070672	2	71
Hs_BMP_Signalling_and_Regulation_WP1425_74390	0.07214113	1	12
Hs_Reversible_Hydration_of_Carbon_Dioxide_WP2770_77043	0.07214113	1	12
Hs_Arrhythmogenic_Right_Ventricular_Cardiomyopathy_WP2118_71265	0.07779294	2	78
Hs_miRNA_Regulation_of_DNA_Damage_Response_WP1530_78503	0.07779294	2	98
Hs_miRNA_Regulation_of_DNA_Damage_Response_WP1530_79564	0.07779294	2	98
Hs_Estrogen_Receptor_Pathway_WP2881_79519	0.07791334	1	13
Hs_Estrogen_Receptor_Pathway_WP2881_79603	0.07791334	1	13
Hs_Homologous_recombination_WP186_68935	0.07791334	1	13
Hs_Osteopontin_Signaling_WP1434_78545	0.07791334	1	13
Hs_SRF_and_miRs_in_Smooth_Muscle_Differentiation_and_Proliferation_WP1991_75261	0.07791334	1	17
Hs_Extrinsic_Pathway_for_Apoptosis_WP1814_77023	0.08364974	1	17
Hs_Prolactin_receptor_signaling_WP2678_78711	0.08364974	1	15
Hs_Transcriptional_activation_by_NRF2_WP3_79527	0.08364974	1	15
Hs_MAPK_Signaling_Pathway_WP382_72103	0.0878676	3	168
Hs_Cori_Cycle_WP1946_74091	0.089350566	1	22
Hs_Activation_of_Matrix_Metalloproteinases_WP2769_77041	0.09501603	1	16
Hs_Cori_Cycle_WP1946_79691	0.09501603	1	23
Hs_Estrogen_metabolism_WP697_63185	0.09501603	1	18
Hs_Apoptosis_WP254_78808	0.09649918	2	84
Hs_Mitochondrial_LC-Fatty_Acid_Beta-Oxidation_WP368_79271	0.10064636	1	17
Hs_Metabolism_of_amino_acids_and_derivatives_WP2693_76898	0.10522473	3	184
Hs_Growth_hormone_receptor_signaling_WP2657_76835	0.10624177	1	20
Hs_Tamoxifen_metabolism_WP691_69462	0.10624177	1	21
Hs_miR-targeted_genes_in_adipocytes_-_TarBase_WP2001_78529	0.10624177	1	38
Hs_Androgen_receptor_signaling_pathway_WP138_79277	0.106283404	2	89
Hs_Focal_Adhesion_WP306_78800	0.10910741	3	188
Hs_Focal_Adhesion_WP306_79698	0.11172636	3	188
Hs_Mitochondrial_Gene_Expression_WP391_71373	0.11180247	1	19
Hs_Signaling_by_Hippo_WP2714_76930	0.11180247	1	20
Hs_miRNAs_involved_in_DNA_damage_response_WP1545_78559	0.11180247	1	69
Hs_Incretin_Synthesis,_Secretion,_and_Inactivation_WP2728_76946	0.11732869	1	21
Hs_Degradation_of_collagen_WP2708_76921	0.12282062	1	21
Hs_Double-Strand_Break_Repair_WP1807_76953	0.12282062	1	21
Hs_Signaling_by_BMP_WP2760_77018	0.12282062	1	21
Hs_Cell_Differentiation_-_meta_WP2023_68892	0.1282785	1	67
Hs_Nucleosome_assembly_WP1874_76826	0.1282785	1	22
Hs_Wnt_Signaling_Pathway_and_Pluripotency_WP399_79474	0.12866244	2	101
Hs_Angiogenesis_WP1539_78807	0.1337025	1	24
Hs_Metabolism_of_steroid_hormones_and_vitamin_D_WP2749_76990	0.1337025	1	23
Hs_Signaling_by_Rho_GTPases_WP1917_76820	0.1337025	1	23
Hs_Cell_Cycle_WP179_70629	0.13494402	2	103
Hs_IL1_and_megakaryotyces_in_obesity_WP2865_78483	0.13909288	1	24
Hs_Physiological_and_Pathological_Hypertrophy_of_the_Heart_WP1528_78581	0.13909288	1	24
Hs_Regulation_of_beta-cell_development_WP1897_77067	0.13909288	1	28
Hs_Triacylglyceride_Synthesis_WP325_71223	0.13909288	1	24
Hs_Post-translational_modification-_synthesis_of_GPI-anchored_proteins_WP1887_77093	0.1444498	1	26
Hs_YAP1-_and_WWTR1_(TAZ)-stimulated_gene_expression_WP2738_76971	0.1444498	1	25
Hs_Regulatory_RNA_pathways_WP1901_76851	0.14977351	1	26
Hs_Signaling_by_NOTCH2_WP2718_76935	0.14977351	1	28
Hs_Extracellular_vesicle-mediated_signaling_in_recipient_cells_WP2870_78078	0.15506418	1	30
Hs_Extracellular_vesicle-mediated_signaling_in_recipient_cells_WP2870_79555	0.16554728	1	30
Hs_Generic_Transcription_Pathway_WP1822_77033	0.1707401	1	30
Hs_Oxidative_Stress_WP408_78546	0.1707401	1	30
Hs_Constitutive_Androstane_Receptor_Pathway_WP2875_79540	0.17590071	1	32

Hs_Constitutive_Androstane_Receptor_Pathway_WP2875_79600	0.17590071	1	32
Hs_Gastric_cancer_network_2_WP2363_76329	0.17590071	1	32
Hs_Inositol_phosphate_metabolism_WP2741_76978	0.17590071	1	31
Hs_Matrix_Metalloproteinases_WP129_72054	0.17590071	1	31
Hs_miR-targeted_genes_in_squamous_cell_-_TarBase_WP2006_78523	0.1805464	2	160
Hs_Assembly_of_collagen_fibrils_and_other_multimeric_structures_WP2798_77089	0.1810293	1	33
Hs_Elastic_fibre_formation_WP2666_76849	0.1810293	1	32
Hs_Monoamine_Transport_WP727_68928	0.1810293	1	32
Hs_Ovarian_Infertility_Genes_WP34_72115	0.1810293	1	32
Hs_Pregnane_X_Receptor_pathway_WP2876_79537	0.1810293	1	33
Hs_Pregnane_X_Receptor_pathway_WP2876_79556	0.1810293	1	33
Hs_Monoamine_GPCRs_WP58_69046	0.18612607	1	34
Hs_miR-targeted_genes_in_leukocytes_-_TarBase_WP2003_78572	0.18724363	2	160
Hs_p38_MAPK_Signaling_Pathway_WP400_72084	0.19119123	1	34
Hs_TGF_beta_Signaling_Pathway_WP366_79341	0.1962256	2	131
Hs_Interleukin-1_signaling_WP1839_76943	0.20619889	1	38
Hs_ATM_Signaling_Pathway_WP2516_78531	0.2111395	1	41
Hs_Amyotrophic_lateral_sclerosis_(ALS)_WP2447_75221	0.2111395	1	38
Hs_FAS_pathway_and_Stress_induction_of_HSP_regulation_WP314_71366	0.2111395	1	38
Hs_Signaling_by_NOTCH1_WP2720_76937	0.21604945	1	42
Hs_Hair_Follicle_Development-_Induction_(Part_1_of_3)_WP2804_78710	0.23538648	1	44
Hs_IL-6_signaling_pathway_WP364_78561	0.23538648	1	43
Hs_Heart_Development_WP1591_78590	0.24014597	1	47
Hs_Tryptophan_metabolism_WP465_77387	0.24957654	1	80
Hs_Tryptophan_metabolism_WP465_79226	0.24957654	1	80
Hs_Energy_Metabolism_WP1541_68947	0.25424796	1	47
Hs_Signaling_by_FGFR_WP1911_76830	0.25889042	1	69
Hs_Differentiation_Pathway_WP2848_78558	0.26350406	1	50
Hs_Glycolysis_and_Gluconeogenesis_WP534_78585	0.26350406	1	49
Hs_Translation_Factors_WP107_78489	0.26808906	1	50
Hs_SIDS_Susceptibility_Pathways_WP706_78533	0.26933166	2	166
Hs_Collagen_biosynthesis_and_modifying_enzymes_WP2725_76944	0.2726456	1	51
Hs_Synaptic_Vesicle_Pathway_WP2267_78595	0.2726456	1	51
Hs_Transport_of_glucose_and_other_sugars,_bile_salts_and_organic_acids,_metal_ions_and_amine_compounds_WP1935_76949	0.2726456	1	52
Hs_Wnt_Signaling_Pathway_Netpath_WP363_78571	0.2726456	1	51
Hs_Cardiac_Progenitor_Differentiation_WP2406_73324	0.27717385	1	53
Hs_Interferon_type_I_signaling_pathways_WP585_79096	0.28614625	1	54
Hs_NGF_signalling_via_TRKA_from_the_plasma_membrane_WP1873_76973	0.28614625	1	57
Hs_TGF_Beta_Signaling_Pathway_WP560_68944	0.28614625	1	55
Hs_IL-1_signaling_pathway_WP195_78528	0.29059073	1	55
Hs_Metapathway_biotransformation_WP702_73516	0.2945886	2	188
Hs_Pathogenic_Escherichia_coli_infection_WP2272_78594	0.29500762	1	64
Hs_Activation_of_Gene_Expression_by_SREBP_(SREBF)_WP2706_76917	0.29939708	1	60
Hs_Kit_receptor_signaling_pathway_WP304_78799	0.30809444	1	59
Hs_Oxidative_phosphorylation_WP623_79520	0.3124027	1	63
Hs_Cytosolic_sensors_of_pathogen-associated_DNA_WP2794_77085	0.3166842	1	62
Hs_Integrin_cell_surface_interactions_WP1833_77019	0.3209391	1	64
Hs_Angiogenesis_overview_WP1993_71385	0.32936984	1	65
Hs_Histone_Modifications_WP2369_69927	0.32936984	1	67
Hs_Oncostatin_M_Signaling_Pathway_WP2374_73668	0.333546	1	65
Hs_AGE-RAGE_pathway_WP2324_78487	0.33769625	1	66
Hs_TSH_signaling_pathway_WP2032_78548	0.33769625	1	66
Hs_Wnt_Signaling_Pathway_WP428_79528	0.33769625	1	67
Hs_G1_to_S_cell_cycle_control_WP45_71377	0.3459196	1	68
Hs_Integrated_Pancreatic_Cancer_Pathway_WP2377_71228	0.35137606	2	200
Hs_Factors_involved_in_megakaryocyte_development_and_platelet_production_WP1815_77027	0.35404116	1	71
Hs_PIP3_activates_AKT_signaling_WP2653_76821	0.35404116	1	90
Hs_L1CAM_interactions_WP1843_76876	0.35806414	1	75
Hs_RNA_Polymerase_I,_RNA_Polymerase_III,_and_Mitochondrial_Transcription_WP1905_77034	0.3739079	1	76
Hs_Prolactin_Signaling_Pathway_WP2037_78501	0.37780756	1	76
Hs_Hair_Follicle_Development-_Cytodifferentiation_(Part_3_of_3)_WP2840_78512	0.39316547	1	87
Hs_Signaling_Pathways_in_Glioblastoma_WP2261_78522	0.40070212	1	82
Hs_Metabolism_of_carbohydrates_WP1848_76833	0.40443537	1	87
Hs_Alzheimers_Disease_WP2059_79302	0.41549662	1	146
Hs_Eukaryotic_Translation_Elongation_WP1811_77053	0.41549662	1	91
Hs_GPCRs,_Other_WP117_71231	0.43347877	1	118
Hs_GPCRs,_Other_WP117_79657	0.43700847	1	118
Hs_GPCRs,_Other_WP117_79718	0.43700847	1	118
Hs_G_Protein_Signaling_Pathways_WP35_71252	0.43700847	1	92
Hs_B_Cell_Receptor_Signaling_Pathway_WP23_78566	0.44051623	1	94
Hs_DNA_Damage_Response_(only_ATM_dependent)_WP710_70109	0.4440022	1	97
Hs_Mitotic_Prometaphase_WP2652_76819	0.44746652	1	98
Hs_Potassium_Channels_WP2669_76853	0.45773092	1	99
Hs_Electron_Transport_Chain_WP111_79220	0.46111	1	104
Hs_Integrin-mediated_Cell_Adhesion_WP185_71391	0.46111	1	99
Hs_Senescence_and_Autophagy_WP615_71375	0.48418278	1	106
Hs_Spinal_Cord_Injury_WP2431_78556	0.50627065	1	116
Hs_Gastrin-CREB_signalling_pathway_via_PKC_and_MAPK_WP2664_76844	0.5724178	1	147
Hs_NRF2_pathway_WP2884_79518	0.5803672	1	143
Hs_NRF2_pathway_WP2884_79616	0.5803672	1	143
Hs_BDNF_signaling_pathway_WP2380_79265	0.5855848	1	141
Hs_Regulation_of_toll-like_receptor_signaling_pathway_WP1449_77378	0.58816934	1	150
Hs_Regulation_of_toll-like_receptor_signaling_pathway_WP1449_79550	0.59073776	1	150
Hs_Mitotic_Metaphase_and_Anaphase_WP2757_77009	0.60581636	1	153
Hs_Calcium_Regulation_in_the_Cardiac_Cell_WP536_78588	0.60827506	1	150
Hs_Insulin_Signaling_WP481_72080	0.6343323	1	161
Hs_EGF-EGFR_Signaling_Pathway_WP437_79266	0.63661367	1	162
Hs_GPCR_downstream_signaling_WP1824_76910	0.6951623	2	406
Hs_GPCRs,_Class_A_Rhodopsin-like_WP455_78510	0.79320866	1	262
Hs_GPCRs,_Class_A_Rhodopsin-like_WP455_79717	0.79320866	1	262
Hs_GPCR_ligand_binding_WP1825_76977	0.88959473	1	371

Table S10 Pathway analysis through WikiPathways in the fibroblast irradiated with UV-C (downregulation, 4hr, 5J/m^2)	p-value (high dose 4hr)	Matched Entities (high dose 4hr)	Pathway Entities of Experiment Type (high dose 4hr)
Hs_EGF-EGFR_Signaling_Pathway_WP437_79266	2.43E-09	18	162
Hs_YAP1-_and_WWTR1_(TAZ)-stimulated_gene_expression_WP2738_76971	1.36E-08	8	25
Hs_Circadian_Clock_WP1797_76871	2.50E-08	10	49
Hs_Regulation_of_Lipid_Metabolism_by_Peroxisome_proliferator-activated_receptor_alpha_(PPARalpha)_WP2797_77088	3.56E-08	14	118
Hs_Regulation_of_Microtubule_Cytoskeleton_WP2038_78570	9.95E-08	9	44
Hs_Androgen_receptor_signaling_pathway_WP138_79277	1.30E-07	12	89
Hs_Transcriptional_Regulation_of_White_Adipocyte_Differentiation_WP2751_76992	1.36E-07	11	74
Hs_TGF_beta_Signaling_Pathway_WP366_79341	2.32E-07	14	131
Hs_Regulation_of_Actin_Cytoskeleton_WP51_79526	9.55E-07	14	149
Hs_Insulin_Signaling_WP481_72080	2.83E-06	14	161
Hs_Mesodermal_Commitment_Pathway_WP2857_78577	5.68E-06	9	154
Hs_Nuclear_Receptors_WP170_71083	6.66E-06	7	38
Hs_Cardiac_Hypertrophic_Response_WP2795_78544	8.00E-06	8	54
Hs_ErbB_Signaling_Pathway_WP673_69914	8.00E-06	8	54
Hs_Activation_of_Gene_Expression_by_SREBP_(SREBF)_WP2706_76917	1.21E-05	8	60
Hs_MAPK_Signaling_Pathway_WP382_72103	2.23E-05	13	168
Hs_miR-targeted_genes_in_muscle_cell_-_TarBase_WP2005_78538	2.75E-05	19	409
Hs_Signaling_by_SCF-KIT_WP2713_76929	4.73E-05	6	39
Hs_Wnt_Signaling_Pathway_Netpath_WP363_78571	4.93E-05	7	51
Hs_Signaling_by_NOTCH1_WP2720_76937	8.91E-05	6	42
Hs_Integrin-mediated_Cell_Adhesion_WP185_71391	1.18E-04	9	99
Hs_Signaling_by_EGFR_WP1910_77036	1.37E-04	6	46
Hs_miR-targeted_genes_in_lymphocytes_-_TarBase_WP2004_78524	1.47E-04	20	495
Hs_Signaling_Pathways_in_Glioblastoma_WP2261_78522	1.72E-04	8	82
Hs_MicroRNAs_in_cardiomyocyte_hypertrophy_WP1544_75258	2.03E-04	8	104
Hs_Focal_Adhesion_WP306_78800	2.44E-04	12	188
Hs_Mitotic_G2-G2-M_phases_WP1859_77022	2.59E-04	8	89
Hs_Focal_Adhesion_WP306_79698	2.69E-04	12	188
Hs_G_Protein_Signaling_Pathways_WP35_71252	3.80E-04	8	92
Hs_Nephrin_interactions_WP1867_76942	4.02E-04	4	22
Hs_Signaling_by_Robo_receptor_WP1918_76868	4.95E-04	4	21
Hs_Signaling_by_ERBB4_WP2781_77063	5.70E-04	5	44
Hs_Prolactin_Signaling_Pathway_WP2037_78501	6.18E-04	7	76
Hs_miR-targeted_genes_in_squamous_cell_-_TarBase_WP2006_78523	6.37E-04	9	160
Hs_Wnt_Signaling_Pathway_and_Pluripotency_WP399_79474	6.65E-04	8	101
Hs_G13_Signaling_Pathway_WP524_72112	7.36E-04	5	38
Hs_Kit_receptor_signaling_pathway_WP304_78799	8.92E-04	6	59
Hs_miR-targeted_genes_in_epithelium_-_TarBase_WP2002_78530	8.98E-04	14	345
Hs_Binding_of_RNA_by_Insulin-like_Growth_Factor-2_mRNA_Binding_Proteins_(IGF2BPs-IMPs-VICKZs)_WP1789_77054	0.001070988	2	13
Hs_Transcriptional_activity_of_SMAD2-SMAD3-SMAD4_heterotrimer_WP2755_77005	0.001171198	5	44
Hs_IL-6_signaling_pathway_WP364_78561	0.001304759	5	43
Hs_BMP_Signalling_and_Regulation_WP1425_74390	0.001328252	3	12
Hs_Endochondral_Ossification_WP474_72122	0.001370163	6	64
Hs_BDNF_signaling_pathway_WP2380_79265	0.001580337	9	141
Hs_AGE-RAGE_pathway_WP2324_78487	0.001608171	6	66
Hs_Extracellular_vesicle-mediated_signaling_in_recipient_cells_WP2870_78078	0.001612291	4	30
Hs_Corticotropin-releasing_hormone_WP2355_78490	0.001680476	7	90
Hs_SRF_and_miRs_in_Smooth_Muscle_Differentiation_and_Proliferation_WP1991_75261	0.001702376	3	17
Hs_Corticotropin-releasing_hormone_WP2355_79562	0.001791133	7	91
Hs_Signaling_by_ERBB2_WP2780_77062	0.001852918	4	36
Hs_Nanoparticle-mediated_activation_of_receptor_signaling_WP2643_74251	0.001852918	4	28
Hs_Energy_Metabolism_WP1541_68947	0.001953392	5	47
Hs_Serotonin_Receptor_2_and_STAT3_Signaling_WP733_74441	0.002114906	2	4
Hs_Extracellular_vesicle-mediated_signaling_in_recipient_cells_WP2870_79555	0.00211731	4	30
Hs_Mitotic_Telophase-Cytokinesis_WP2765_77030	0.002136133	3	14
Hs_Factors_involved_in_megakaryocyte_development_and_platelet_production_WP1815_77027	0.002177041	6	71
Hs_Constitutive_Androstane_Receptor_Pathway_WP2875_79600	0.002721979	4	32
Hs_Constitutive_Androstane_Receptor_Pathway_WP2875_79540	0.002721979	4	32
Hs_Neural_Crest_Differentiation_WP2064_79263	0.002892853	7	101
Hs_Nuclear_Receptors_Meta-Pathway_WP2882_78569	0.00298034	14	318
Hs_Pregnane_X_Receptor_pathway_WP2876_79556	0.003064508	4	33
Hs_Ovarian_Infertility_Genes_WP34_72115	0.003064508	4	32
Hs_Pregnane_X_Receptor_pathway_WP2876_79537	0.003064508	4	33
Hs_Signaling_of_Hepatocyte_Growth_Factor_Receptor_WP313_71094	0.003435305	4	34
Hs_Alpha_6_Beta_4_signaling_pathway_WP244_78506	0.003435305	4	33
Hs_Adipogenesis_WP236_78584	0.003538112	8	131
Hs_Ectoderm_Differentiation_WP2858_78578	0.003706843	8	145
Hs_Ectoderm_Commitment_Pathway_WP2856_78535	0.003706843	8	145
Hs_Integrated_Breast_Cancer_Pathway_WP1984_72732	0.004358921	9	164
Hs_Gastrin-CREB_signalling_pathway_via_PKC_and_MAPK_WP2664_76844	0.004645743	8	147
Hs_Notch_Signaling_Pathway_WP61_78592	0.006095653	5	61
Hs_Semaphorin_interactions_WP1907_76850	0.006528605	5	62
Hs_Double-Strand_Break_Repair_WP1807_76953	0.007069924	3	21
Hs_Signaling_by_BMP_WP2760_77018	0.007069924	3	21
Hs_Mitotic_G1-G1-S_phases_WP1858_76928	0.007534949	7	120
Hs_Wnt_Signaling_Pathway_WP428_79528	0.008476322	5	67
Hs_JAK-STAT_WP2593_74127	0.008923217	4	45
Hs_JAK-STAT_WP2594_74128	0.008923217	4	45
Hs_Sphingolipid_metabolism_WP2788_77079	0.008923217	4	46
Hs_Nanoparticle_triggered_autophagic_cell_death_WP2509_78509	0.009152977	3	23
Hs_DNA_Damage_Response_(only_ATM_dependent)_WP710_70109	0.009234387	6	97
Hs_Mitotic_Prometaphase_WP2652_76819	0.009702458	6	98
Hs_Myometrial_Relaxation_and_Contraction_Pathways_WP289_78540	0.009950098	8	156
Hs_PIP3_activates_AKT_signaling_WP2653_76821	0.010793019	5	90
Hs_L1CAM_interactions_WP1843_76876	0.011433373	5	75
Hs_EPO_Receptor_Signaling_WP581_67199	0.01288355	3	26
Hs_Arylhydrocarbon_receptor_(AhR)_signaling_pathway_WP2100_74081	0.014292457	3	28
Hs_T-Cell_Receptor_and_Co-stimulatory_Signaling_WP2583_75360	0.015785202	3	32
Hs_Interleukin-2_signaling_WP2732_76959	0.015785202	3	29
Hs_Hair_Follicle_Development-_Cytodifferentiation_(Part_3_of_3)_WP2840_78512	0.018387688	5	87
Hs_DAG_and_IP3_signaling_WP2688_76890	0.019024005	3	30
Hs_Colchicine_Metabolic_Pathway_WP2536_74479	0.019024512	1	1
Hs_TGF_Beta_Signaling_Pathway_WP560_68944	0.019427067	4	55
Hs_NGF_signalling_via_TRKA_from_the_plasma_membrane_WP1873_76973	0.019427067	4	57
Hs_Hypothetical_Network_for_Drug_Addiction_WP666_68893	0.02077065	3	32
Hs_Opioid_Signalling_WP1978_76919	0.021906639	4	56
Hs_Fc_epsilon_receptor_(FCERI)_signaling_WP2759_77017	0.02430484	5	218
Hs_Signaling_by_TGF-beta_Receptor_Complex_WP2742_76980	0.024519023	3	36

Hs_Meiotic_Synapsis_WP2731_76957	0.024519023	3	35
Hs_Calcium_Regulation_in_the_Cardiac_Cell_WP536_78588	0.02517635	7	150
Hs_Prolactin_receptor_signaling_WP2678_78711	0.028277297	2	15
Hs_Leptin_signaling_pathway_WP2034_79545	0.030463047	4	62
Hs_Interleukin-3,_5_and_GM-CSF_signaling_WP1840_77073	0.030777857	3	37
Hs_B_Cell_Receptor_Signaling_Pathway_WP23_78566	0.032605376	5	94
Hs_Histone_Modifications_WP2369_69927	0.033694718	4	67
Hs_Angiogenesis_overview_WP1993_71385	0.033694718	4	65
Hs_Hedgehog_Signaling_Pathway_WP47_78542	0.03636951	2	16
Hs_Integrated_Pancreatic_Cancer_Pathway_WP2377_71228	0.037550163	8	200
Hs_Felbamate_Metabolism_WP2816_76465	0.03768745	1	4
Hs_Lidocaine_metabolism_WP2646_74430	0.03768745	1	2
Hs_Neurotransmitter_uptake_and_Metabolism_In_Glial_Cells_WP1872_76956	0.03768745	1	2
Hs_DAP12_interactions_WP2694_76901	0.037792813	3	45
Hs_IL-5_Signaling_Pathway_WP127_78498	0.040296827	3	40
Hs_G1_to_S_cell_cycle_control_WP45_71377	0.040734664	4	68
Hs_Endoderm_Differentiation_WP2853_78496	0.040734664	4	146
Hs_Netrin-1_signaling_WP1868_76847	0.042882595	3	42
Hs_Glycosaminoglycan_metabolism_WP2743_76981	0.042882595	3	41
Hs_Growth_hormone_receptor_signaling_WP2657_76835	0.045231797	2	20
Hs_Cell_Cycle_WP179_70629	0.047206417	5	103
Hs_Signaling_by_PDGF_WP1916_76874	0.048296332	3	48
Hs_Hair_Follicle_Development-_Induction_(Part_1_of_3)_WP2804_78710	0.048296332	3	44
Hs_Integrin_alphaIIb_beta3_signaling_WP1832_76825	0.04992986	2	21
Hs_miRNAs_involved_in_DNA_damage_response_WP1545_78559	0.04992986	2	69
Hs_Signaling_by_Insulin_receptor_WP1913_77046	0.050617963	4	75
Hs_Arrhythmogenic_Right_Ventricular_Cardiomyopathy_WP2118_71265	0.05273851	4	78
Hs_Aryl_Hydrocarbon_Receptor_pathway_WP2873_79544	0.054027256	3	46
Hs_NCAM_signaling_for_neurite_out-growth_WP1866_76927	0.054027256	3	48
Hs_Aryl_Hydrocarbon_Receptor_Pathway_WP2873_79696	0.054027256	3	46
Hs_GPVI-mediated_activation_cascade_WP1826_76817	0.054794744	2	21
Hs_PDGF_Pathway_WP2526_78551	0.054794744	2	37
Hs_Oxytocin_signaling_WP2889_78766	0.055995684	1	3
Hs_Aryl_Hydrocarbon_Receptor_WP2586_78547	0.057009302	3	47
Hs_Muscle_contraction_WP1864_76895	0.060067713	3	49
Hs_Prostate_Cancer_WP2263_73838	0.061272983	5	115
Hs_Diurnally_Regulated_Genes_with_Circadian_Orthologs_WP410_69903	0.06320141	3	48
Hs_Signaling_by_FGFR_WP1911_76830	0.06320141	3	69
Hs_Cell_Differentiation_-_meta_WP2023_68892	0.06499307	2	67
Hs_Mitotic_Metaphase_and_Anaphase_WP2757_77009	0.06603219	6	153
Hs_Transport_of_vitamins,_nucleosides,_and_related_molecules_WP1937_77048	0.070311174	2	24
Hs_Oxytocin_signaling_WP2889_79688	0.073955946	1	5
Hs_DNA_Damage_Bypass_WP1803_76966	0.073955946	1	4
Hs_Pilocytic_astrocytoma_WP2253_74520	0.073955946	1	4
Hs_Cardiac_Progenitor_Differentiation_WP2406_73324	0.076466165	3	53
Hs_Neurotransmitter_Receptor_Binding_And_Downstream_Transmission_In_The_Postsynaptic_Cell_WP2754_77001	0.07746007	5	124
Hs_Apoptosis-related_network_due_to_altered_Notch3_in_ovarian_cancer_WP2864_79278	0.079958804	3	53
Hs_IL-4_Signaling_Pathway_WP395_78576	0.083519496	3	55
Hs_PPAR_Alpha_Pathway_WP2878_79689	0.087054096	2	26
Hs_PPAR_alpha_pathway_WP2878_79532	0.087054096	2	26
Hs_RANKL-RANK_Signaling_Pathway_WP2018_79274	0.08714693	3	55
Hs_TCA_Cycle_Nutrient_Utilization_and_Invasiveness_of_Ovarian_Cancer_WP2868_78079	0.09157484	1	5
Hs_PI_Metabolism_WP2747_76987	0.092875026	2	29
Hs_TCR_Signaling_Pathway_WP69_72111	0.09297597	4	92
Hs_Signaling_by_Type_1_Insulin-like_Growth_Factor_1_Receptor_(IGF1R)_WP2677_76867	0.094596684	3	75
Hs_Lipid_storage_and_perilipins_in_skeletal_muscle_WP2887_78335	0.108858846	1	6
Hs_Hair_Follicle_Development-_Organogenesis_(Part_2_of_3)_WP2839_78519	0.11096736	2	31
Hs_Gastric_cancer_network_2_WP2363_76329	0.11718783	2	32
Hs_miRs_in_Muscle_Cell_Differentiation_WP2012_68959	0.11718783	2	40
Hs_IL17_signaling_pathway_WP2112_63216	0.11718783	2	31
Hs_Monoamine_Transport_WP727_68928	0.12349352	2	32
Hs_Vitamin_D_Receptor_Pathway_WP2877_79534	0.12581433	1	7
Hs_Oncostatin_M_Signaling_Pathway_WP2374_73668	0.12679037	3	65
Hs_Nuclear_Receptors_in_Lipid_Metabolism_and_Toxicity_WP299_78587	0.12987898	2	35
Hs_TSH_signaling_pathway_WP2032_78548	0.13106047	3	66
Hs_Pre-NOTCH_Expression_and_Processing_WP2786_77076	0.13633887	2	38
Hs_p38_MAPK_Signaling_Pathway_WP400_72084	0.13633887	2	34
Hs_Non-homologous_end_joining_WP438_68977	0.14244753	1	8
Hs_Aflatoxin_B1_metabolism_WP699_70509	0.14244753	1	8
Hs_Nifedipine_Activity_WP259_75227	0.14244753	1	10
Hs_Genes_and_(Common)_Pathways_Underlying_Drug_Addiction_WP2636_73720	0.14244753	1	12
Hs_Codeine_and_Morphine_Metabolism_WP1604_74317	0.14244753	1	9
Hs_Senescence_and_Autophagy_WP615_71375	0.1439653	4	106
Hs_Glucocorticoid_Receptor_Pathway_WP2880_79522	0.1486244	3	71
Hs_Glucocorticoid_Receptor_Pathway_WP2880_79615	0.1486244	3	71
Hs_Glycogen_Metabolism_WP500_63201	0.14946161	2	36
Hs_Type_II_interferon_signaling_(IFNG)_WP619_71168	0.1561147	2	37
Hs_Response_to_elevated_platelet_cytosolic_Ca2+_WP1903_76962	0.15876456	1	9
Hs_RalA_downstream_regulated_genes_WP2290_69039	0.15876456	1	9
Hs_Benzo(a)pyrene_metabolism_WP696_72081	0.15876456	1	9
Hs_Metastatic_brain_tumor_WP2249_76471	0.15876456	1	27
Hs_ATM_Signaling_Pathway_WP2516_78531	0.16282266	2	41
Hs_miRNA_Regulation_of_DNA_Damage_Response_WP1530_79564	0.16689217	3	98
Hs_miRNA_Regulation_of_DNA_Damage_Response_WP1530_78503	0.16689217	3	98
Hs_S_Phase_WP2772_77049	0.16908969	4	116
Hs_RNA_Polymerase_I,_RNA_Polymerase_III,_and_Mitochondrial_Transcription_WP1905_77034	0.17155823	3	76
Hs_Liver_X_Receptor_Pathway_WP2874_79599	0.17477143	1	10
Hs_MAPK_targets-_Nuclear_events_mediated_by_MAP_kinases_WP1845_76899	0.17477143	1	10
Hs_Liver_X_Receptor_Pathway_WP2874_79542	0.17477143	1	10
Hs_NOD_pathway_WP1433_68991	0.17638546	2	41
Hs_Integrated_Lung_Cancer_Pathway_WP2512_71235	0.18323177	2	44
Hs_Signaling_by_the_B_Cell_Receptor_(BCR)_WP2746_76984	0.19561395	4	239
Hs_Interleukin-11_Signaling_Pathway_WP2332_79525	0.20398207	2	44
Hs_Heart_Development_WP1591_78590	0.20398207	2	47
Hs_Apoptotic_execution_phase_WP1784_76813	0.20398207	2	46
Hs_Estrogen_Receptor_Pathway_WP2881_79519	0.22098939	1	13
Hs_Estrogen_Receptor_Pathway_WP2881_79603	0.22098939	1	13
Hs_Homologous_recombination_WP186_68935	0.22098939	1	13
Hs_Cytosolic_Iron-sulfur_Cluster_Assembly_WP2690_76892	0.22098939	1	13
Hs_Regulation_of_Insulin-like_Growth_Factor_(IGF)_Transport_and_Uptake_by_Insulin-like_Growth_Factor_Binding_Proteins_(IGFBPs)_WP2799_77094	0.22098939	1	13

Hs_Signaling_by_Activin_WP2791_77082	0.22098939	1	13
Hs_miR-targeted_genes_in_leukocytes_-_TarBase_WP2003_78572	0.2233056	4	160
Hs_TNF_alpha_Signaling_Pathway_WP2808_78568	0.22500509	3	87
Hs_Irinotecan_Pathway_WP229_68389	0.23581341	1	14
Hs_Transcriptional_activation_by_NRF2_WP3_79527	0.23581341	1	15
Hs_Thrombin_signalling_through_proteinase_activated_receptors_(PARs)_WP1929_77002	0.23581341	1	15
Hs_Signal_amplification_WP1908_76822	0.23581341	1	15
Hs_Fatty_acid,_triacylglycerol,_and_ketone_body_metabolism_WP1817_77087	0.23905161	2	49
Hs_IL-3_Signaling_Pathway_WP286_78583	0.23905161	2	49
Hs_TNF_alpha_Signaling_Pathway_WP231_79280	0.2401134	3	90
Hs_Fatty_Acid_Omega_Oxidation_WP206_68882	0.2503556	1	15
Hs_TarBasePathway_WP1992_78296	0.2503556	1	18
Hs_Translocation_of_GLUT4_to_the_Plasma_Membrane_WP2777_77058	0.25317225	2	51
Hs_Regulation_of_Apoptosis_WP1896_77071	0.2602405	2	53
Hs_ID_signaling_pathway_WP53_67360	0.26462135	1	16
Hs_Syndecan_interactions_WP2787_77077	0.26462135	1	16
Hs_SREBF_and_miR33_in_cholesterol_and_lipid_homeostasis_WP2011_75253	0.26462135	1	18
Hs_Estrogen_metabolism_WP697_63185	0.26462135	1	18
Hs_Signaling_by_NODAL_WP2675_76863	0.26462135	1	17
Hs_Drug_Induction_of_Bile_Acid_Pathway_WP2289_78511	0.27861586	1	17
Hs_Serotonin_Receptor_2_and_ELK-SRF-GATA4_signaling_WP732_74437	0.27861586	1	17
Hs_Mitochondrial_LC-Fatty_Acid_Beta-Oxidation_WP368_79271	0.27861586	1	17
Hs_RIG-I-MDA5_mediated_induction_of_IFN-alpha-beta_pathways_WP1904_76841	0.2814446	2	56
Hs_Pathogenic_Escherichia_coli_infection_WP2272_78594	0.28850403	2	64
Hs_miR-targeted_genes_in_adipocytes_-_TarBase_WP2001_78529	0.29234436	1	38
Hs_Tamoxifen_metabolism_WP691_69462	0.29234436	1	21
Hs_Farnesoid_X_Receptor_Pathway_WP2879_79602	0.30581182	1	19
Hs_Small_Ligand_GPCRs_WP247_74422	0.30581182	1	19
Hs_Farnesoid_X_Receptor_Pathway_WP2879_79524	0.30581182	1	19
Hs_Glutathione_metabolism_WP100_74146	0.30581182	1	37
Hs_Mitochondrial_Gene_Expression_WP391_71373	0.30581182	1	19
Hs_Signaling_by_Hippo_WP2714_76930	0.30581182	1	20
Hs_Peroxisomal_lipid_metabolism_WP1878_76900	0.30581182	1	21
Hs_Fanconi_Anemia_pathway_WP1816_76931	0.31902325	1	22
Hs_Urea_cycle_and_metabolism_of_amino_groups_WP497_72142	0.31902325	1	37
Hs_Hypertrophy_Model_WP516_71358	0.31902325	1	20
Hs_Incretin_Synthesis,_Secretion,_and_Inactivation_WP2728_76946	0.31902325	1	21
Hs_Sphingolipid_Metabolism_WP1422_78591	0.31902325	1	21
Hs_Insulin_Processing_WP2736_76967	0.31902325	1	20
Hs_Metabolism_of_water-soluble_vitamins_and_cofactors_WP1857_76875	0.33061185	2	67
Hs_Glycerophospholipid_Biosynthetic_Pathway_WP2533_76320	0.33198348	1	34
Hs_Type_II_diabetes_mellitus_WP1584_72094	0.33198348	1	22
Hs_TCR_signaling_WP1927_76950	0.33756945	2	74
Hs_Folate_Metabolism_WP176_74202	0.34450486	2	67
Hs_Bile_acid_and_bile_salt_metabolism_WP1788_76958	0.34469733	1	23
Hs_Detoxification_of_Reactive_Oxygen_Species_WP2824_76144	0.34469733	1	26
Hs_Globo_Sphingolipid_Metabolism_WP1424_71392	0.34469733	1	24
Hs_Nucleosome_assembly_WP1874_76826	0.34469733	1	22
Hs_Signaling_by_Rho_GTPases_WP1917_76820	0.35716942	1	23
Hs_GPCRs,_Class_B_Secretin-like_WP334_79716	0.35716942	1	23
Hs_GPCRs,_Class_B_Secretin-like_WP334_63204	0.35716942	1	23
Hs_Angiogenesis_WP1539_78807	0.35716942	1	24
Hs_Pan-cancer_miRNA-target_network_involving_ECM-receptor_interactions_WP2911_79678	0.35716942	1	45
Hs_Signal_Transduction_of_S1P_Receptor_WP26_78492	0.36940438	1	25
Hs_Physiological_and_Pathological_Hypertrophy_of_the_Heart_WP1528_78581	0.36940438	1	24
Hs_Triacylglyceride_Synthesis_WP325_71223	0.36940438	1	24
Hs_DNA_Damage_Response_WP707_78527	0.37199238	2	68
Hs_AMPK_Signaling_WP1403_79471	0.37199238	2	68
Hs_Toll-Like_Receptors_Cascades_WP2775_77055	0.3814067	1	27
Hs_IL-7_Signaling_Pathway_WP205_79472	0.3814067	1	25
Hs_Regulatory_RNA_pathways_WP1901_76851	0.3931808	1	26
Hs_FSH_signaling_pathway_WP2035_78536	0.404731	1	27
Hs_mRNA_Capping_WP1861_76815	0.4160616	1	28
Hs_Platelet_homeostasis_WP1885_77031	0.4160616	1	31
Hs_Fcgamma_receptor_(FCGR)_dependent_phagocytosis_WP2719_76936	0.41890097	2	210
Hs_M-G1_Transition_WP2785_77074	0.42545882	2	79
Hs_MAPK_Cascade_WP422_72129	0.42717674	1	29
Hs_Meiotic_Recombination_WP2698_76904	0.42717674	1	29
Hs_HIV_Life_Cycle_WP2658_76836	0.43566236	3	143
Hs_Oxidative_Stress_WP408_78546	0.43808052	1	30
Hs_Generic_Transcription_Pathway_WP1822_77033	0.43808052	1	30
Hs_Dopaminergic_Neurogenesis_WP2855_79211	0.43808052	1	30
Hs_Inositol_phosphate_metabolism_WP2741_76978	0.44877693	1	31
Hs_Prostaglandin_Synthesis_and_Regulation_WP98_72088	0.44877693	1	31
Hs_Matrix_Metalloproteinases_WP129_72054	0.44877693	1	31
Hs_Bladder_Cancer_WP2828_79529	0.44877693	1	31
Hs_Integration_of_energy_metabolism_WP1831_77011	0.4512923	2	81
Hs_Selenium_Micronutrient_Network_WP15_78776	0.45764711	2	84
Hs_Elastic_fibre_formation_WP2666_76849	0.45926994	1	32
Hs_Endothelin_Pathways_WP2197_74852	0.46956342	1	33
Hs_MHC_class_II_antigen_presentation_WP2679_76872	0.4764514	2	90
Hs_TOR_Signaling_WP1471_70031	0.47966114	1	34
Hs_Fatty_Acid_Beta_Oxidation_WP143_71172	0.47966114	1	34
Hs_Alzheimers_Disease_WP2059_79302	0.4887646	2	146
Hs_NRF2_pathway_WP2884_79616	0.49440825	3	143
Hs_NRF2_pathway_WP2884_79518	0.49440825	3	143
Hs_RB_in_Cancer_WP2446_78573	0.49485272	2	87
Hs_Integrated_Cancer_pathway_WP1971_71249	0.49928412	1	36
Hs_Nucleotide-binding_domain,_leucine_rich_repeat_containing_receptor_(NLR)_signaling_pathways_WP2763_77025	0.49928412	1	38
Hs_Telomere_Maintenance_WP1928_76893	0.49928412	1	37
Hs_Cell_surface_interactions_at_the_vascular_wall_WP1794_77039	0.5008944	2	91
Hs_FAS_pathway_and_Stress_induction_of_HSP_regulation_WP314_71366	0.5181678	1	38
Hs_Striated_Muscle_Contraction_WP383_68970	0.5181678	1	38
Hs_Interferon_gamma_signaling_WP1836_77096	0.5181678	1	43
Hs_Amyotrophic_lateral_sclerosis_(ALS)_WP2447_75221	0.5181678	1	38
Hs_Synthesis_of_DNA_WP1925_76968	0.51873744	2	94
Hs_Transport_of_inorganic_cations-anions_and_amino_acids-oligopeptides_WP1936_76845	0.5273411	1	43
Hs_TWEAK_Signaling_Pathway_WP2036_78525	0.5451677	1	41
Hs_Hematopoietic_Stem_Cell_Differentiation_WP2849_78586	0.5451677	1	98
Hs_IL-2_Signaling_Pathway_WP49_78543	0.55382746	1	42
Hs_DNA_Replication_WP466_76196	0.55382746	1	42

Hs_RNA_Polymerase_II_Transcription_WP1906_76887	0.57506204	2	101
Hs_Toll-like_receptor_signaling_pathway_WP75_72133	0.5804225	2	102
Hs_Tryptophan_metabolism_WP465_79226	0.5868505	1	80
Hs_Tryptophan_metabolism_WP465_77387	0.5868505	1	80
Hs_Selenium_Metabolism_and_Selenoproteins_WP28_71888	0.5868505	1	48
Hs_Notch_Signaling_Pathway_WP268_70096	0.5868505	1	46
Hs_Structural_Pathway_of_Interleukin_1_(IL-1)_WP2637_79580	0.59471744	1	49
Hs_Structural_Pathway_of_Interleukin_1_(IL-1)_WP2637_76330	0.59471744	1	49
Hs_TSLP_Signaling_Pathway_WP2203_78549	0.59471744	1	48
Hs_GPCR_downstream_signaling_WP1824_76910	0.6053419	7	406
Hs_Differentiation_Pathway_WP2848_78558	0.61000526	1	50
Hs_Deadenylation-dependent_mRNA_decay_WP2659_76837	0.61000526	1	49
Hs_Vitamin_B12_Metabolism_WP1533_70117	0.6247169	1	53
Hs_Transport_of_glucose_and_other_sugars,_bile_salts_and_organic_acids,_metal_ions_and_amine_compounds_WP1935_7694	0.6247169	1	52
Hs_Synaptic_Vesicle_Pathway_WP2267_78595	0.6247169	1	51
Hs_Cell_Cycle_Checkpoints_WP1775_76816	0.6264196	2	115
Hs_ISG15_antiviral_mechanism_WP2672_76856	0.6318635	1	61
Hs_Spinal_Cord_Injury_WP2431_78556	0.6360934	2	116
Hs_Degradation_of_beta-catenin_by_the_destruction_complex_WP2773_77050	0.6388741	1	55
Hs_Metapathway_biotransformation_WP702_73516	0.6455854	3	188
Hs_IL-1_signaling_pathway_WP195_78528	0.6524978	1	55
Hs_Costimulation_by_the_CD28_family_WP1799_77064	0.66560805	1	71
Hs_Glycerophospholipid_biosynthesis_WP2740_76974	0.6719767	1	59
Hs_Cell_junction_organization_WP1793_77057	0.6843527	1	61
Hs_Oxidation_by_Cytochrome_P450_WP43_73490	0.6843527	1	63
Hs_mRNA_Processing_WP411_71369	0.6900319	2	127
Hs_Oxidation_by_Cytochrome_P450_WP43_79585	0.6903647	1	63
Hs_Cytosolic_sensors_of_pathogen-associated_DNA_WP2794_77085	0.6903647	1	62
Hs_SREBP_signalling_WP1982_78494	0.6962623	1	65
Hs_Integrin_cell_surface_interactions_WP1833_77019	0.6962623	1	64
Hs_Regulation_of_DNA_replication_WP1898_76824	0.724109	1	70
Hs_Primary_Focal_Segmental_Glomerulosclerosis_FSGS_WP2572_79296	0.7494051	1	74
Hs_Regulation_of_toll-like_receptor_signaling_pathway_WP1449_77378	0.7549841	2	150
Hs_Regulation_of_toll-like_receptor_signaling_pathway_WP1449_79550	0.75841206	2	150
Hs_Membrane_Trafficking_WP1846_76873	0.7588623	1	78
Hs_Apoptosis_Modulation_and_Signaling_WP1772_63162	0.78514796	1	93
Hs_Regulation_of_mRNA_Stability_by_Proteins_that_Bind_AU-rich_Elements_WP2733_76960	0.78514796	1	83
Hs_Eukaryotic_Translation_Termination_WP1813_77051	0.797197	1	88
Hs_Apoptosis_WP254_78808	0.8010614	1	84
Hs_Eukaryotic_Translation_Elongation_WP1811_77053	0.8085709	1	91
Hs_Allograft_Rejection_WP2328_78554	0.8085709	1	100
Hs_Cytoplasmic_Ribosomal_Proteins_WP477_67139	0.8122188	1	88
Hs_SIDS_Susceptibility_Pathways_WP706_78533	0.81870055	2	166
Hs_GPCRs,_Other_WP117_71231	0.82612896	1	118
Hs_GPCRs,_Other_WP117_79657	0.82944256	1	118
Hs_GPCRs,_Other_WP117_79718	0.82944256	1	118
Hs_Potassium_Channels_WP2669_76853	0.84804064	1	99
Hs_Human_Complement_System_WP2806_78589	0.8592996	1	136
Hs_SRP-dependent_cotranslational_protein_targeting_to_membrane_WP2737_76970	0.8646126	1	111
Hs_Nonsense-Mediated_Decay_WP2710_76924	0.8671934	1	111
Hs_Iron_uptake_and_transport_WP2670_76854	0.8770346	1	114
Hs_Eukaryotic_Translation_Initiation_WP1812_76969	0.88167846	1	117
Hs_Host_Interactions_of_HIV_factors_WP2684_76883	0.8965951	1	137
Hs_Metabolism_of_amino_acids_and_derivatives_WP2693_76898	0.9698625	1	184
Hs_Influenza_Life_Cycle_WP2683_76880	0.9698625	1	217
Hs_GPCRs,_Class_A_Rhodopsin-like_WP455_79717	0.99218893	1	262
Hs_GPCRs,_Class_A_Rhodopsin-like_WP455_78510	0.99218893	1	262
Hs_GPCR_ligand_binding_WP1825_76977	0.99886864	1	371

Table S11 Pathway analysis through WikiPathways in the fibroblast irradiated with UV-C (downregulation, 12hr, 0.5J/m^2)	p-value(low dose 12hr)	Matched Entities (low dose 12hr)	Pathway Entities of Experiment Type (low dose 12hr)
Hs_Regulation_of_toll-like_receptor_signaling_pathway_WP1449_77378	1.19E-06	8	150
Hs_Regulation_of_toll-like_receptor_signaling_pathway_WP1449_79550	1.26E-06	8	150
Hs_Toll-like_receptor_signaling_pathway_WP75_72133	1.50E-06	7	102
Hs_Senescence_and_Autophagy_WP615_71375	1.94E-06	7	106
Hs_Insulin_Signaling_WP481_72080	3.05E-06	8	161
Hs_Cytokines_and_Inflammatory_Response_WP530_79331	1.14E-05	4	30
Hs_Costimulation_by_the_CD28_family_WP1799_77064	1.49E-05	5	71
Hs_TCR_signaling_WP1927_76950	2.43E-05	5	74
Hs_Glucocorticoid_Receptor_Pathway_WP2880_79615	4.06E-05	5	71
Hs_Interleukin-1_signaling_WP1839_76943	4.81E-05	4	38
Hs_Interferon_gamma_signaling_WP1836_77096	5.36E-05	4	43
Hs_Selenium_Micronutrient_Network_WP15_78776	8.20E-05	5	84
Hs_Allograft_Rejection_WP2328_78554	1.09E-04	5	100
Hs_TCR_Signaling_Pathway_WP69_72111	1.35E-04	5	92
Hs_Vitamin_B12_Metabolism_WP1533_70117	1.72E-04	4	53
Hs_Folate_Metabolism_WP176_74202	4.13E-04	4	67
Hs_Glucocorticoid_Receptor_Pathway_WP2880_79522	5.81E-04	4	71
Hs_Monoamine_Transport_WP727_68928	6.92E-04	3	32
Hs_Nuclear_Receptors_WP170_71083	0.00114925	3	38
Hs_MHC_class_II_antigen_presentation_WP2679_76872	0.001152491	4	90
Hs_MicroRNAs_in_cardiomyocyte_hypertrophy_WP1544_75258	0.001152491	4	104
Hs_TNF_alpha_Signaling_Pathway_WP2808_78568	0.001257745	4	87
Hs_TNF_alpha_Signaling_Pathway_WP231_79280	0.001427957	4	90
Hs_Signaling_by_NOTCH3_WP2722_76939	0.001553633	2	11
Hs_Signaling_by_NOTCH4_WP2721_76938	0.001553633	2	11
Hs_Myometrial_Relaxation_and_Contraction_Pathways_WP289_78540	0.001615804	5	156
Hs_Nuclear_Receptors_Meta-Pathway_WP2882_78569	0.001691634	7	318
Hs_SIDS_Susceptibility_Pathways_WP706_78533	0.002013413	5	166
Hs_Effects_of_PIP2_hydrolysis_WP1809_76979	0.002187644	2	13
Hs_MAPK_Signaling_Pathway_WP382_72103	0.002295601	5	168
Hs_Differentiation_Pathway_WP2848_78558	0.00240238	3	50
Hs_Cardiac_Hypertrophic_Response_WP2795_78544	0.00317068	3	54
Hs_IL-1_signaling_pathway_WP195_78528	0.003340318	3	55
Hs_RIG-I-MDA5_mediated_induction_of_IFN-alpha-beta_pathways_WP1904_76841	0.003340318	3	56
Hs_Spinal_Cord_Injury_WP2431_78556	0.003404089	4	116
Hs_Ganglio_Sphingolipid_Metabolism_WP1423_79262	0.003760295	2	18
Hs_Serotonin_Receptor_2_and_ELK-SRF-GATA4_signaling_WP732_74437	0.003760295	2	17
Hs_Signaling_by_the_B_Cell_Receptor_(BCR)_WP2746_76984	0.004217314	4	239
Hs_AGE-RAGE_pathway_WP2324_78487	0.005577757	3	66
Hs_Signaling_by_BMP_WP2760_77018	0.005724193	2	21
Hs_Type_II_diabetes_mellitus_WP1584_72094	0.005724193	2	22
Hs_TGF_beta_Signaling_Pathway_WP366_79341	0.005744525	4	131
Hs_EBV_LMP1_signaling_WP262_70166	0.006847397	2	23
Hs_EBV_LMP1_signaling_WP262_79554	0.00744338	2	24
Hs_Toll-Like_Receptors_Cascades_WP2775_77055	0.008061932	2	27
Hs_Signaling_by_NOTCH2_WP2718_76935	0.008702794	2	28
Hs_PI_Metabolism_WP2747_76987	0.009365708	2	29
Hs_MyD88-Mal_cascade_initiated_on_plasma_membrane_WP2761_77020	0.009365708	2	28
Hs_Oxidative_Stress_WP408_78546	0.011484215	2	30
Hs_Alzheimers_Disease_WP2059_79302	0.011527676	3	146
Hs_Assembly_of_collagen_fibrils_and_other_multimeric_structures_WP2798_77089	0.013002183	2	33
Hs_Ovarian_Infertility_Genes_WP34_72115	0.013002183	2	32
Hs_Elastic_fibre_formation_WP2666_76849	0.013002183	2	32
Hs_Signaling_by_TGF-beta_Receptor_Complex_WP2742_76980	0.013792114	2	36
Hs_Serotonin_HTR1_Group_and_FOS_Pathway_WP722_74511	0.013792114	2	34
Hs_Nucleotide-binding_domain,_leucine_rich_repeat_containing_receptor_(NLR)_signaling_pathways_WP2763_77025	0.01628278	2	38
Hs_Signaling_by_NOTCH1_WP2720_76937	0.018949755	2	42
Hs_GPCR_downstream_signaling_WP1824_76910	0.019431662	6	406
Hs_NOD_pathway_WP1433_68991	0.019876832	2	41
Hs_Integrated_Lung_Cancer_Pathway_WP2512_71235	0.02082257	2	44
Hs_TWEAK_Signaling_Pathway_WP2036_78525	0.02082257	2	41
Hs_Hematopoietic_Stem_Cell_Differentiation_WP2849_78586	0.02082257	2	98
Hs_Transcriptional_activity_of_SMAD2-SMAD3-SMAD4_heterotrimer_WP2755_77005	0.021786738	2	44
Hs_IL-6_signaling_pathway_WP364_78561	0.022769112	2	43
Hs_Hair_Follicle_Development-_Induction_(Part_1_of_3)_WP2804_78710	0.022769112	2	44
Hs_Integrated_Pancreatic_Cancer_Pathway_WP2377_71228	0.023430986	4	200
Hs_Interleukin-11_Signaling_Pathway_WP2332_79525	0.023769468	2	44
Hs_Aryl_Hydrocarbon_Receptor_Pathway_WP2873_79696	0.024787584	2	46
Hs_Aryl_Hydrocarbon_Receptor_pathway_WP2873_79544	0.024787584	2	46
Hs_Notch_Signaling_Pathway_WP268_70096	0.025823241	2	46
Hs_Muscle_contraction_WP1864_76895	0.026876219	2	49
Hs_Signalling_by_NGF_WP1976_76994	0.026876219	2	50
Hs_TSLP_Signaling_Pathway_WP2203_78549	0.026876219	2	48
Hs_Circadian_Clock_WP1797_76871	0.029033279	2	49
Hs_Translation_Factors_WP107_78489	0.030136932	2	50
Hs_Translocation_of_GLUT4_to_the_Plasma_Membrane_WP2777_77058	0.031257056	2	51
Hs_Collagen_biosynthesis_and_modifying_enzymes_WP2725_76944	0.031257056	2	51
Hs_Ectoderm_Differentiation_WP2858_78578	0.034588758	3	145
Hs_Ectoderm_Commitment_Pathway_WP2856_78535	0.034588758	3	145
Hs_TGF_Beta_Signaling_Pathway_WP560_68944	0.03471414	2	55
Hs_RANKL-RANK_Signaling_Pathway_WP2018_79274	0.03589806	2	55
Hs_Interleukin-6_signaling_WP2704_76915	0.03726854	1	11
Hs_Signaling_by_Type_1_Insulin-like_Growth_Factor_1_Receptor_(IGF1R)_WP2677_76867	0.03831201	2	75
Hs_NLR_Proteins_WP288_70187	0.04247841	1	10
Hs_Notch_Signaling_Pathway_WP61_78592	0.043318838	2	61
Hs_Leptin_signaling_pathway_WP2034_79545	0.044606682	2	62
Hs_Regulation_of_Actin_Cytoskeleton_WP51_79526	0.046051297	3	149
Hs_Endochondral_Ossification_WP474_72122	0.047224395	2	64
Hs_Response_to_elevated_platelet_cytosolic_Ca2+_WP1903_76962	0.04766018	1	9
Hs_Oncostatin_M_Signaling_Pathway_WP2374_73668	0.048553888	2	65
Hs_AMPK_Signaling_WP1403_79471	0.0526227	2	68
Hs_Gene_regulatory_network_modelling_somitogenesis_WP2854_77681	0.05281401	1	11
Hs_Platelet_Aggregation_(Plug_Formation)_WP1884_76986	0.05281401	1	10
Hs_alpha-linolenic_(omega3)_and_linoleic_(omega6)_acid_metabolism_WP2724_76941	0.05281401	1	11
Hs_Signaling_by_VEGF_WP1919_76864	0.05281401	1	10
Hs_PIP3_activates_AKT_signaling_WP2653_76821	0.055400357	2	90
Hs_Serotonin_Transporter_Activity_WP1455_68965	0.05794005	1	11
Hs_Primary_Focal_Segmental_Glomerulosclerosis_FSGS_WP2572_79296	0.05822844	2	74
Hs_Alanine_and_aspartate_metabolism_WP106_74147	0.063038446	1	40
Hs_BMP_Signalling_and_Regulation_WP1425_74390	0.063038446	1	12

Hs_Peptide_hormone_biosynthesis_WP2691_76894	0.063038446	1	12
Hs_Regulation_of_Insulin-like_Growth_Factor_(IGF)_Transport_and_Uptake_by_Insulin-like_Growth_Factor_Binding_Proteins_(IGFBPs)_WP2799_77094	0.06810934	1	13
Hs_Signaling_by_Activin_WP2791_77082	0.06810934	1	13
Hs_Quercetin_and_Nf-kB-_AP-1_induced_cell_apoptosis_WP2435_72165	0.06810934	1	15
Hs_Osteopontin_Signaling_WP1434_78545	0.06810934	1	13
Hs_Integration_of_energy_metabolism_WP1831_77011	0.07001734	2	81
Hs_Apoptosis_Modulation_and_Signaling_WP1772_63162	0.07001734	2	93
Hs_Prolactin_receptor_signaling_WP2678_78711	0.0731529	1	15
Hs_Quercetin_and_Nf-kB-_AP-1_Induced_Cell_Apoptosis_WP2435_79692	0.0731529	1	15
Hs_Quercetin_and_Nf-kB-_AP-1_Induced_Cell_Apoptosis_WP2435_79583	0.0731529	1	15
Hs_Osteoblast_Signaling_WP322_79496	0.0731529	1	14
Hs_Apoptosis_WP254_78808	0.07617899	2	84
Hs_GABA_synthesis,_release,_reuptake_and_degradation_WP2685_76885	0.07816925	1	16
Hs_Fc_epsilon_receptor_(FCERI)_signaling_WP2759_77017	0.07932211	2	218
Hs_Focal_Adhesion_WP306_78800	0.079656	3	188
Hs_Focal_Adhesion_WP306_79698	0.08165574	3	188
Hs_Osteoclast_Signaling_WP12_78593	0.08315855	1	16
Hs_G_Protein_Signaling_Pathways_WP35_71252	0.08898573	2	92
Hs_Growth_hormone_receptor_signaling_WP2657_76835	0.09305657	1	20
Hs_MyD88_cascade_initiated_on_plasma_membrane_WP2801_77090	0.09305657	1	19
Hs_Nanomaterial_induced_apoptosis_WP2507_78557	0.09305657	1	18
Hs_Polycystic_Kidney_Disease_Pathway_WP2571_78508	0.09305657	1	18
Hs_Serotonin_Receptor_4-6-7_and_NR3C_Signaling_WP734_74438	0.097965576	1	19
Hs_Peroxisomal_lipid_metabolism_WP1878_76900	0.097965576	1	21
Hs_Electron_Transport_Chain_WP111_79220	0.10067048	2	104
Hs_Neural_Crest_Differentiation_WP2064_79263	0.10067048	2	101
Hs_Fanconi_Anemia_pathway_WP1816_76931	0.102848105	1	22
Hs_MyD88_dependent_cascade_initiated_on_endosome_WP2768_77040	0.1077043	1	21
Hs_Bile_acid_and_bile_salt_metabolism_WP1788_76958	0.1125343	1	23
Hs_Nucleosome_assembly_WP1874_76826	0.1125343	1	22
Hs_Globo_Sphingolipid_Metabolism_WP1424_71392	0.1125343	1	24
Hs_Detoxification_of_Reactive_Oxygen_Species_WP2824_76144	0.1125343	1	26
Hs_Transport_of_vitamins,_nucleosides,_and_related_molecules_WP1937_77048	0.117338255	1	24
Hs_TAK1_activates_NFkB_by_phosphorylation_and_activation_of_IKKs_complex_WP2656_76831	0.117338255	1	24
Hs_Nanoparticle_triggered_autophagic_cell_death_WP2509_78509	0.117338255	1	23
Hs_Pan-cancer_miRNA-target_network_involving_ECM-receptor_interactions_WP2911_79678	0.117338255	1	45
Hs_Estrogen_signaling_pathway_WP712_78491	0.117338255	1	23
Hs_MyD88-independent_cascade_WP2752_76995	0.12211629	1	25
Hs_IL1_and_megakaryotyces_in_obesity_WP2865_78483	0.12211629	1	24
Hs_Regulation_of_Lipid_Metabolism_by_Peroxisome_proliferator-activated_receptor_alpha_(PPARalpha)_WP2797_77088	0.1251826	2	118
Hs_Activation_of_Genes_by_ATF4_WP2753_76999	0.12686856	1	25
Hs_Mitotic_G1-G1-S_phases_WP1858_76928	0.134253	2	120
Hs_T-Cell_Receptor_and_Co-stimulatory_Signaling_WP2583_75360	0.1409721	1	32
Hs_Platelet_homeostasis_WP1885_77031	0.1409721	1	31
Hs_DAG_and_IP3_signaling_WP2688_76890	0.15024811	1	30
Hs_Dopaminergic_Neurogenesis_WP2855_79211	0.15024811	1	30
Hs_Bladder_Cancer_WP2828_79529	0.15484862	1	31
Hs_miRs_in_Muscle_Cell_Differentiation_WP2012_68959	0.15484862	1	40
Hs_IL17_signaling_pathway_WP2112_63216	0.15484862	1	31
Hs_Adipogenesis_WP236_78584	0.15657598	2	131
Hs_Mitochondrial_Protein_Import_WP2717_76934	0.1594243	1	32
Hs_Class_I_MHC_mediated_antigen_processing_&_presentation_WP2796_77098	0.16226254	2	148
Hs_Monoamine_GPCRs_WP58_69046	0.16397531	1	34
Hs_Gastrin-CREB_signalling_pathway_via_PKC_and_MAPK_WP2664_76844	0.16798587	2	147
Hs_Fatty_Acid_Beta_Oxidation_WP143_71172	0.16850176	1	34
Hs_Signaling_by_ERBB4_WP2781_77063	0.17748155	1	44
Hs_BDNF_signaling_pathway_WP2380_79265	0.1775992	2	141
Hs_Type_II_interferon_signaling_(IFNG)_WP619_71168	0.18193513	1	37
Hs_Striated_Muscle_Contraction_WP383_68970	0.18636468	1	38
Hs_miR-targeted_genes_in_epithelium_-_TarBase_WP2002_78530	0.18744715	3	345
Hs_Calcium_Regulation_in_the_Cardiac_Cell_WP536_78588	0.19510455	2	150
Hs_Interferon_alpha-beta_signaling_WP1835_77078	0.20384519	1	45
Hs_Selenium_Metabolism_and_Selenoproteins_WP28_71888	0.22095145	1	48
Hs_Tryptophan_metabolism_WP465_77387	0.22095145	1	80
Hs_Tryptophan_metabolism_WP465_79226	0.22095145	1	80
Hs_Structural_Pathway_of_Interleukin_1_(IL-1)_WP2637_76330	0.22517048	1	49
Hs_Structural_Pathway_of_Interleukin_1_(IL-1)_WP2637_79580	0.22517048	1	49
Hs_Signaling_by_FGFR_WP1911_76830	0.22936673	1	69
Hs_Glycolysis_and_Gluconeogenesis_WP534_78585	0.23354036	1	49
Hs_IL-3_Signaling_Pathway_WP286_78583	0.23354036	1	49
Hs_Wnt_Signaling_Pathway_Netpath_WP363_78571	0.24182013	1	51
Hs_Regulation_of_Apoptosis_WP1896_77071	0.24592653	1	53
Hs_ISG15_antiviral_mechanism_WP2672_76856	0.24592653	1	61
Hs_Degradation_of_beta-catenin_by_the_destruction_complex_WP2773_77050	0.2500108	1	55
Hs_Apoptosis-related_network_due_to_altered_Notch3_in_ovarian_cancer_WP2864_79278	0.2500108	1	53
Hs_Interferon_type_I_signaling_pathways_WP585_79096	0.25407296	1	54
Hs_IL-4_Signaling_Pathway_WP395_78576	0.25407296	1	55
Hs_Metabolism_of_amino_acids_and_derivatives_WP2693_76898	0.25860822	2	184
Hs_Opioid_Signalling_WP1978_76919	0.26213172	1	56
Hs_Extracellular_matrix_organization_WP2703_76914	0.27010372	1	58
Hs_Glycerophospholipid_biosynthesis_WP2740_76974	0.27010372	1	59
Hs_Kit_receptor_signaling_pathway_WP304_78799	0.27405748	1	59
Hs_Oxidation_by_Cytochrome_P450_WP43_73490	0.27798986	1	63
Hs_Cell_junction_organization_WP1793_77057	0.27798986	1	61
Hs_Oxidation_by_Cytochrome_P450_WP43_79585	0.28190106	1	63
Hs_Cytosolic_sensors_of_pathogen-associated_DNA_WP2794_77085	0.28190106	1	62
Hs_Integrin_cell_surface_interactions_WP1833_77019	0.28579113	1	64
Hs_Proteasome_Degradation_WP183_71712	0.29350838	1	65
Hs_Histone_Modifications_WP2369_69927	0.29350838	1	67
Hs_Visual_phototransduction_WP2776_77056	0.2973358	1	67
Hs_GPCR_ligand_binding_WP1825_76977	0.29735908	3	371
Hs_TSH_signaling_pathway_WP2032_78548	0.30114254	1	66
Hs_Wnt_Signaling_Pathway_WP428_79528	0.30114254	1	67
Hs_Regulation_of_DNA_replication_WP1898_76824	0.30492875	1	70
Hs_Mesodermal_Commitment_Pathway_WP2857_78577	0.3086945	1	154
Hs_G1_to_S_cell_cycle_control_WP45_71377	0.3086945	1	68
Hs_Signaling_by_Insulin_receptor_WP1913_77046	0.32722056	1	75
Hs_Arrhythmogenic_Right_Ventricular_Cardiomyopathy_WP2118_71265	0.33086598	1	78
Hs_RNA_Polymerase_I,_RNA_Polymerase_III,_and_Mitochondrial_Transcription_WP1905_77034	0.3344917	1	76
Hs_Fcgamma_receptor_(FCGR)_dependent_phagocytosis_WP2719_76936	0.3344917	1	210
Hs_Prolactin_Signaling_Pathway_WP2037_78501	0.33809784	1	76
Hs_M-G1_Transition_WP2785_77074	0.33809784	1	79

Hs_APC-C-mediated_degradation_of_cell_cycle_proteins_WP1782_77060	0.34168452	1	80
Hs_Regulation_of_mRNA_Stability_by_Proteins_that_Bind_AU-rich_Elements_WP2733_76960	0.3523288	1	83
Hs_Signaling_Pathways_in_Glioblastoma_WP2261_78522	0.35932952	1	82
Hs_Metabolism_of_carbohydrates_WP1848_76833	0.36280155	1	87
Hs_Eukaryotic_Translation_Elongation_WP1811_77053	0.37310556	1	91
Hs_Mitotic_G2-G2-M_phases_WP1859_77022	0.3765032	1	89
Hs_RB_in_Cancer_WP2446_78573	0.3765032	1	87
Hs_miR-targeted_genes_in_lymphocytes_-_TarBase_WP2004_78524	0.38406736	3	495
Hs_Respiratory_electron_transport,_ATP_synthesis_by_chemiosmotic_coupling,_and_heat_production_by_uncoupling_proteins._ WP1902_77091	0.3865864	1	94
Hs_Corticotropin-releasing_hormone_WP2355_78490	0.3865864	1	90
Hs_Corticotropin-releasing_hormone_WP2355_79562	0.38991123	1	91
Hs_Synthesis_of_DNA_WP1925_76968	0.38991123	1	94
Hs_GPCRs,_Class_A_Rhodopsin-like_WP455_78510	0.39615896	2	262
Hs_GPCRs,_Class_A_Rhodopsin-like_WP455_79717	0.39615896	2	262
Hs_B_Cell_Receptor_Signaling_Pathway_WP23_78566	0.3965071	1	94
Hs_DNA_Damage_Response_(only_ATM_dependent)_WP710_70109	0.39977837	1	97
Hs_Potassium_Channels_WP2669_76853	0.41268763	1	99
Hs_Integrin-mediated_Cell_Adhesion_WP185_71391	0.41587147	1	99
Hs_Human_Complement_System_WP2806_78589	0.42532018	1	136
Hs_Cell_Cycle_WP179_70629	0.4284358	1	103
Hs_Iron_uptake_and_transport_WP2670_76854	0.44677913	1	114
Hs_Cell_Cycle_Checkpoints_WP1775_76816	0.45276228	1	115
Hs_Eukaryotic_Translation_Initiation_WP1812_76969	0.45276228	1	117
Hs_S_Phase_WP2772_77049	0.45868093	1	116
Hs_Host_Interactions_of_HIV_factors_WP2684_76883	0.4732	1	137
Hs_Neurotransmitter_Receptor_Binding_And_Downstream_Transmission_In_The_Postsynaptic_Cell_WP2754_77001	0.4760569	1	124
Hs_miR-targeted_genes_in_squamous_cell_-_TarBase_WP2006_78523	0.49011153	1	160
Hs_miR-targeted_genes_in_leukocytes_-_TarBase_WP2003_78572	0.49836335	1	160
Hs_NRF2_pathway_WP2884_79616	0.53006095	1	143
Hs_NRF2_pathway_WP2884_79518	0.53006095	1	143
Hs_Mitotic_Metaphase_and_Anaphase_WP2757_77009	0.5549453	1	153
Hs_Integrated_Breast_Cancer_Pathway_WP1984_72732	0.58983123	1	164
Hs_Metapathway_biotransformation_WP702_73516	0.6115609	1	188
Hs_Influenza_Life_Cycle_WP2683_76880	0.6281156	1	217
Hs_miR-targeted_genes_in_muscle_cell_-_TarBase_WP2005_78538	0.8376451	1	409

Table S12 Pathway analysis through WikiPathways in the fibroblast irradiated with UV-C (downregulation, 12hr, 5J/m^2)	p-value (high dose 12hr)	Matched Entities (high dose 12hr)	Pathway Entities of Experiment Type (high dose 12hr)
Hs_Myometrial_Relaxation_and_Contraction_Pathways_WP289_78540	5.16E-09	14	156
Hs_Arrhythmogenic_Right_Ventricular_Cardiomyopathy_WP2118_71265	2.28E-07	9	78
Hs_Opioid_Signalling_WP1978_76919	3.03E-07	8	56
Hs_Calcium_Regulation_in_the_Cardiac_Cell_WP536_78588	1.86E-06	11	150
Hs_Senescence_and_Autophagy_WP615_71375	4.86E-06	9	106
Hs_Netrin-1_signaling_WP1868_76847	8.20E-06	6	42
Hs_DAG_and_IP3_signaling_WP2688_76890	2.48E-05	5	30
Hs_Cardiac_Hypertrophic_Response_WP2795_78544	4.14E-05	6	54
Hs_Response_to_elevated_platelet_cytosolic_Ca2+_WP1903_76962	1.29E-04	3	9
Hs_Focal_Adhesion_WP306_78800	3.75E-04	9	188
Hs_Focal_Adhesion_WP306_79698	4.06E-04	9	188
Hs_SRF_and_miRs_in_Smooth_Muscle_Differentiation_and_Proliferation_WP1991_75261	4.24E-04	3	17
Hs_Integrin_cell_surface_interactions_WP1833_77019	8.26E-04	5	64
Hs_Semaphorin_interactions_WP1907_76850	8.26E-04	5	62
Hs_Integrin-mediated_Cell_Adhesion_WP185_71391	0.001150613	6	99
Hs_Nuclear_Receptors_Meta-Pathway_WP2882_78569	0.001343978	11	318
Hs_Signaling_by_BMP_WP2760_77018	0.001840475	3	21
Hs_Spinal_Cord_Injury_WP2431_78556	0.002261114	6	116
Hs_Muscle_contraction_WP1864_76895	0.002267067	4	49
Hs_Differentiation_Pathway_WP2848_78558	0.002643728	4	50
Hs_Neurotransmitter_Receptor_Binding_And_Downstream_Transmission_In_The_Postsynaptic_Cell_WP2754_77001	0.002928242	6	124
Hs_MicroRNAs_in_cardiomyocyte_hypertrophy_WP1544_75258	0.00319074	5	104
Hs_Cytokines_and_Inflammatory_Response_WP530_79331	0.003444494	3	30
Hs_Platelet_homeostasis_WP1885_77031	0.004265221	3	31
Hs_TGF_beta_Signaling_Pathway_WP366_79341	0.004690064	6	131
Hs_G_Protein_Signaling_Pathways_WP35_71252	0.004709213	5	92
Hs_Activation_of_Chaperone_Genes_by_ATF6-alpha_WP2655_78848	0.004709819	2	10
Hs_Cell_junction_organization_WP1793_77057	0.005498496	4	61
Hs_Metabolism_of_nucleotides_WP1851_76838	0.005498496	4	65
Hs_Inositol_phosphate_metabolism_WP2741_76978	0.005701817	3	31
Hs_miRs_in_Muscle_Cell_Differentiation_WP2012_68959	0.005701817	3	40
Hs_NRF2_pathway_WP2884_79616	0.006233166	6	143
Hs_NRF2_pathway_WP2884_79518	0.006233166	6	143
Hs_Ovarian_Infertility_Genes_WP34_72115	0.006237328	3	32
Hs_Elastic_fibre_formation_WP2666_76849	0.006237328	3	32
Hs_BDNF_signaling_pathway_WP2380_79265	0.006670525	6	141
Hs_Endothelin_Pathways_WP2197_74852	0.006801847	3	33
Hs_Angiogenesis_overview_WP1993_71385	0.006906164	4	65
Hs_Wnt_Signaling_Pathway_WP428_79528	0.007691511	4	67
Hs_Signaling_by_SCF-KIT_WP2713_76929	0.008019375	3	39
Hs_AMPK_Signaling_WP1403_79471	0.008533376	4	68
Hs_Glycogen_Metabolism_WP500_63201	0.008673049	3	36
Hs_Glucocorticoid_Receptor_Pathway_WP2880_79522	0.009433305	4	71
Hs_Glucocorticoid_Receptor_Pathway_WP2880_79615	0.009433305	4	71
Hs_Integrated_Pancreatic_Cancer_Pathway_WP2377_71228	0.009621215	7	200
Hs_Signaling_by_Activin_WP2791_77082	0.009891798	2	13
Hs_Regulation_of_Insulin-like_Growth_Factor_(IGF)_Transport_and_Uptake_by_Insulin-like_Growth_Factor_Binding_Proteins_(IGFBPs)_WP2799_77094	0.009891798	2	13
Hs_Osteoblast_Signaling_WP322_79496	0.011451178	2	14
Hs_Transcriptional_activation_by_NRF2_WP3_79527	0.011451178	2	15
Hs_Transcriptional_activity_of_SMAD2-SMAD3-SMAD4_heterotrimer_WP2755_77005	0.01324102	3	44
Hs_IL-6_signaling_pathway_WP364_78561	0.014112022	3	43
Hs_Integration_of_energy_metabolism_WP1831_77011	0.014852791	4	81
Hs_Osteoclast_Signaling_WP12_78593	0.014868134	2	16
Hs_Syndecan_interactions_WP2787_77077	0.014868134	2	16
Hs_Energy_dependent_regulation_of_mTOR_by_LKB1-AMPK_WP2748_76988	0.016720623	2	18
Hs_Ganglio_Sphingolipid_Metabolism_WP1423_79262	0.016720623	2	18
Hs_GPCR_downstream_signaling_WP1824_76910	0.017324343	10	406
Hs_MHC_class_II_antigen_presentation_WP2679_76872	0.017472759	4	90
Hs_RB_in_Cancer_WP2446_78573	0.019615075	4	87
Hs_Circadian_Clock_WP1797_76871	0.020009177	3	49
Hs_Metabolism_of_amino_acids_and_derivatives_WP2693_76898	0.021260148	6	184
Hs_Wnt_Signaling_Pathway_Netpath_WP363_78571	0.022232335	3	51
Hs_Gastrin-CREB_signalling_pathway_via_PKC_and_MAPK_WP2664_76844	0.022736035	5	147
Hs_Urea_cycle_and_metabolism_of_amino_groups_WP497_72142	0.022824561	2	37
Hs_Signaling_by_Robo_receptor_WP1918_76868	0.022824561	2	21
Hs_Regulation_of_Apoptosis_WP1896_77071	0.023392325	3	53
Hs_Cardiac_Progenitor_Differentiation_WP2406_73324	0.023392325	3	53
Hs_TGF_Beta_Signaling_Pathway_WP560_68944	0.025809063	3	55
Hs_NGF_signalling_via_TRKA_from_the_plasma_membrane_WP1873_76973	0.025809063	3	57
Hs_Potassium_Channels_WP2669_76853	0.028815614	4	99
Hs_Signaling_by_Type_1_Insulin-like_Growth_Factor_1_Receptor_(IGF1R)_WP2677_76867	0.029675445	3	75
Hs_Pan-cancer_miRNA-target_network_involving_ECM-receptor_interactions_WP2911_79678	0.02969856	2	45
Hs_Regulation_of_Actin_Cytoskeleton_WP51_79526	0.030430749	5	149
Hs_Physiological_and_Pathological_Hypertrophy_of_the_Heart_WP1528_78581	0.03215038	2	24
Hs_Kit_receptor_signaling_pathway_WP304_78799	0.032413088	3	59
Hs_Human_Complement_System_WP2806_78589	0.03269827	4	136
Hs_Cell_Cycle_WP179_70629	0.033714347	4	103
Hs_Activation_of_Genes_by_ATF4_WP2753_76999	0.03467879	2	25
Hs_Endochondral_Ossification_WP474_72122	0.039811242	3	64
Hs_Arachidonic_acid_metabolism_WP2650_76814	0.03995686	2	27
Hs_Cell_Cycle_Checkpoints_WP1775_76816	0.04250048	4	115
Hs_Prostate_Cancer_WP2263_73838	0.04250048	4	115
Hs_T-Cell_Receptor_and_Co-stimulatory_Signaling_WP2583_75360	0.04270237	2	32
Hs_AGE-RAGE_pathway_WP2324_78487	0.0429892	3	66
Hs_SIDS_Susceptibility_Pathways_WP706_78533	0.044246454	5	166
Hs_Regulation_of_DNA_replication_WP1898_76824	0.044624384	3	70
Hs_mRNA_Editing_WP1862_77075	0.04623793	1	4
Hs_Nicotine_Activity_on_Chromaffin_Cells_WP1603_78574	0.04623793	1	4
Hs_G1_to_S_cell_cycle_control_WP45_71377	0.046290126	3	68
Hs_MAPK_Signaling_Pathway_WP382_72103	0.049220216	5	168
Hs_PIP3_activates_AKT_signaling_WP2653_76821	0.04971247	3	90
Hs_Mitotic_G1-G1-S_phases_WP1858_76928	0.05114793	4	120
Hs_Hypothetical_Network_for_Drug_Addiction_WP666_68893	0.051340695	2	32
Hs_L1CAM_interactions_WP1843_76876	0.05146866	3	75
Hs_Signaling_by_the_B_Cell_Receptor_(BCR)_WP2746_76984	0.053782348	4	239
Hs_Monoamine_Transport_WP727_68928	0.054347586	2	32
Hs_Fluoropyrimidine_Activity_WP1601_78516	0.057415053	2	33
Hs_Signaling_of_Hepatocyte_Growth_Factor_Receptor_WP313_71094	0.057415053	2	34
Hs_Signaling_by_TGF-beta_Receptor_Complex_WP2742_76980	0.057415053	2	36

Hs_Sulindac_Metabolic_Pathway_WP2542_70621	0.057459954	1	5
Hs_Fcgamma_receptor_(FCGR)_dependent_phagocytosis_WP2719_76936	0.05878829	3	210
Hs_RNA_Polymerase_I,_RNA_Polymerase_III,_and_Mitochondrial_Transcription_WP1905_77034	0.05878829	3	76
Hs_TOR_Signaling_WP1471_70031	0.060541257	2	34
Hs_p38_MAPK_Signaling_Pathway_WP400_72084	0.060541257	2	34
Hs_M-G1_Transition_WP2785_77074	0.06069072	3	79
Hs_Adipogenesis_WP236_78584	0.06803074	4	131
Hs_Regulation_of_mRNA_Stability_by_Proteins_that_Bind_AU-rich_Elements_WP2733_76960	0.06858289	3	83
Hs_Signaling_Pathways_in_Glioblastoma_WP2261_78522	0.07269467	3	82
Hs_Nuclear_Receptors_WP170_71083	0.07359796	2	38
Hs_Striated_Muscle_Contraction_WP383_68970	0.07359796	2	38
Hs_Interleukin-6_signaling_WP2704_76915	0.07951008	1	11
Hs_Ubiquinol_biosynthesis_WP2734_76961	0.07951008	1	7
Hs_Alzheimers_Disease_WP2059_79302	0.08123822	3	146
Hs_TWEAK_Signaling_Pathway_WP2036_78525	0.08392231	2	41
Hs_Glycosaminoglycan_metabolism_WP2743_76981	0.08392231	2	41
Hs_Hematopoietic_Stem_Cell_Differentiation_WP2849_78586	0.08392231	2	98
Hs_Cell_surface_interactions_at_the_vascular_wall_WP1794_77039	0.08566523	3	91
Hs_Signaling_by_EGFR_WP1910_77036	0.087456375	2	46
Hs_Genes_and_(Common)_Pathways_Underlying_Drug_Addiction_WP2636_73720	0.09034125	1	12
Hs_Unfolded_Protein_Response_WP1939_77024	0.09034125	1	11
Hs_Regulation_of_Microtubule_Cytoskeleton_WP2038_78570	0.091034144	2	44
Hs_Synthesis_of_DNA_WP1925_76968	0.09249291	3	94
Hs_TSLP_Signaling_Pathway_WP2203_78549	0.1057525	2	48
Hs_Energy_Metabolism_WP1541_68947	0.1057525	2	47
Hs_Signaling_by_FGFR_WP1911_76830	0.109526694	2	69
Hs_Gene_regulatory_network_modelling_somitogenesis_WP2854_77681	0.111623354	1	11
Hs_Signal_regulatory_protein_(SIRP)_family_interactions_WP1909_77029	0.111623354	1	11
Hs_Signaling_by_VEGF_WP1919_76864	0.111623354	1	10
Hs_Platelet_Aggregation_(Plug_Formation)_WP1884_76986	0.111623354	1	10
Hs_IL-3_Signaling_Pathway_WP286_78583	0.11333595	2	49
Hs_Wnt_Signaling_Pathway_and_Pluripotency_WP399_79474	0.11424262	3	101
Hs_Transport_of_glucose_and_other_sugars,_bile_salts_and_organic_acids,_metal_ions_and_amine_compounds_WP1935_7694	0.12105429	2	52
Hs_Collagen_biosynthesis_and_modifying_enzymes_WP2725_76944	0.12105429	2	51
Hs_Translocation_of_GLUT4_to_the_Plasma_Membrane_WP2777_77058	0.12105429	2	51
Hs_Synaptic_Vesicle_Pathway_WP2267_78595	0.12105429	2	51
Hs_Serotonin_Transporter_Activity_WP1455_68965	0.122077264	1	11
Hs_Insulin_Signaling_WP481_72080	0.12289058	4	161
Hs_Nonsense-Mediated_Decay_WP2710_76924	0.12708133	3	111
Hs_Degradation_of_beta-catenin_by_the_destruction_complex_WP2773_77050	0.12889709	2	55
Hs_Apoptosis-related_network_due_to_altered_Notch3_in_ovarian_cancer_WP2864_79278	0.12889709	2	53
Hs_Peptide_hormone_biosynthesis_WP2691_76894	0.13240835	1	12
Hs_Alanine_and_aspartate_metabolism_WP106_74147	0.13240835	1	40
Hs_BMP_Signalling_and_Regulation_WP1425_74390	0.13240835	1	12
Hs_Effects_of_PIP2_hydrolysis_WP1809_76979	0.14261806	1	13
Hs_Osteopontin_Signaling_WP1434_78545	0.14261806	1	13
Hs_Platelet_Adhesion_to_exposed_collagen_WP1883_76862	0.14261806	1	13
Hs_S_Phase_WP2772_77049	0.14861703	3	116
Hs_Extracellular_matrix_organization_WP2703_76914	0.1489838	2	58
Hs_Prolactin_receptor_signaling_WP2678_78711	0.15270783	1	15
Hs_Signal_amplification_WP1908_76822	0.15270783	1	15
Hs_Regulation_of_Cholesterol_Biosynthesis_by_SREBP_(SREBF)_WP2686_76888	0.15270783	1	16
Hs_GPCRs,_Class_C_Metabotropic_glutamate,_pheromone_WP501_78580	0.16267905	1	15
Hs_Cell_Differentiation_-_Index_WP2029_69036	0.16267905	1	54
Hs_GABA_synthesis,_release,_reuptake_and_degradation_WP2685_76885	0.16267905	1	16
Hs_GPCRs,_Class_C_Metabotropic_glutamate,_pheromone_WP501_79715	0.16267905	1	15
Hs_Signaling_by_NODAL_WP2675_76863	0.1725331	1	17
Hs_Folate_Metabolism_WP176_74202	0.17382899	2	67
Hs_Proteasome_Degradation_WP183_71712	0.17382899	2	65
Hs_Visual_phototransduction_WP2776_77056	0.17803356	2	67
Hs_Oncostatin_M_Signaling_Pathway_WP2374_73668	0.17803356	2	65
Hs_Metabolism_of_nitric_oxide_WP1850_77097	0.18227138	1	17
Hs_Drug_Induction_of_Bile_Acid_Pathway_WP2289_78511	0.18227138	1	17
Hs_Mesodermal_Commitment_Pathway_WP2857_78577	0.19073687	2	154
Hs_Endoderm_Differentiation_WP2853_78496	0.19073687	2	146
Hs_Growth_hormone_receptor_signaling_WP2657_76835	0.19189523	1	20
Hs_Ectoderm_Commitment_Pathway_WP2856_78535	0.20072505	3	145
Hs_Ectoderm_Differentiation_WP2858_78578	0.20072505	3	145
Hs_Glutathione_metabolism_WP100_74146	0.20140602	1	37
Hs_Integrin_alphaIIb_beta3_signaling_WP1832_76825	0.20140602	1	21
Hs_Mitochondrial_Gene_Expression_WP391_71373	0.20140602	1	19
Hs_Parkin-Ubiquitin_Proteasomal_System_pathway_WP2359_72121	0.20355533	2	73
Hs_Class_I_MHC_mediated_antigen_processing_&_presentation_WP2796_77098	0.20676492	3	148
Hs_Primary_Focal_Segmental_Glomerulosclerosis_FSGS_WP2572_79296	0.20784964	2	74
Hs_Sphingolipid_Metabolism_WP1422_78591	0.21080504	1	21
Hs_PDGF_Pathway_WP2526_78551	0.21080504	1	37
Hs_Eicosanoid_Synthesis_WP167_71381	0.21080504	1	25
Hs_Insulin_Processing_WP2736_76967	0.21080504	1	20
Hs_Signaling_by_Insulin_receptor_WP1913_77046	0.21215329	2	75
Hs_miR-targeted_genes_in_lymphocytes_-_TarBase_WP2004_78524	0.21253838	7	495
Hs_Degradation_of_collagen_WP2708_76921	0.22009362	1	21
Hs_Cell_Differentiation_-_meta_WP2023_68892	0.22927305	1	67
Hs_Nucleosome_assembly_WP1874_76826	0.22927305	1	22
Hs_Bile_acid_and_bile_salt_metabolism_WP1788_76958	0.22927305	1	23
Hs_Globo_Sphingolipid_Metabolism_WP1424_71392	0.22927305	1	24
Hs_APC-C-mediated_degradation_of_cell_cycle_proteins_WP1782_77060	0.22944537	2	80
Hs_EBV_LMP1_signaling_WP262_70166	0.23834462	1	23
Hs_Signaling_by_Rho_GTPases_WP1917_76820	0.23834462	1	23
Hs_Nanoparticle_triggered_autophagic_cell_death_WP2509_78509	0.23834462	1	23
Hs_Angiogenesis_WP1539_78807	0.23834462	1	24
Hs_Apoptosis_Modulation_and_Signaling_WP1772_63162	0.24247447	2	93
Hs_Hair_Follicle_Development-_Cytodifferentiation_(Part_3_of_3)_WP2840_78512	0.24247447	2	87
Hs_Selenium_Micronutrient_Network_WP15_78776	0.24682508	2	84
Hs_Signal_Transduction_of_S1P_Receptor_WP26_78492	0.2473096	1	25
Hs_EBV_LMP1_signaling_WP262_79554	0.2473096	1	24
Hs_Eukaryotic_Translation_Termination_WP1813_77051	0.25553358	2	88
Hs_Toll-Like_Receptors_Cascades_WP2775_77055	0.2561692	1	27
Hs_Eukaryotic_Translation_Elongation_WP1811_77053	0.26860482	2	91
Hs_Fc_epsilon_receptor_(FCERI)_signaling_WP2759_77017	0.26860482	2	218
Hs_TNF_alpha_Signaling_Pathway_WP2808_78568	0.26860482	2	87
Hs_Cytoplasmic_Ribosomal_Proteins_WP477_67139	0.27296165	2	88
Hs_PI_Metabolism_WP2747_76987	0.27357733	1	29
Hs_Extracellular_vesicle-mediated_signaling_in_recipient_cells_WP2870_78078	0.27357733	1	30

Hs_One_Carbon_Metabolism_WP241_79667	0.27357733	1	27
Hs_One_Carbon_Metabolism_WP241_78552	0.27357733	1	27
Hs_FSH_signaling_pathway_WP2035_78536	0.27357733	1	27
Hs_TNF_alpha_Signaling_Pathway_WP231_79280	0.2816714	2	90
Hs_Nanoparticle-mediated_activation_of_receptor_signaling_WP2643_74251	0.28212824	1	28
Hs_Interleukin-2_signaling_WP2732_76959	0.28212824	1	29
Hs_Corticotropin-releasing_hormone_WP2355_78490	0.2860232	2	90
Hs_Corticotropin-releasing_hormone_WP2355_79562	0.2903722	2	91
Hs_Extracellular_vesicle-mediated_signaling_in_recipient_cells_WP2870_79555	0.29057866	1	30
Hs_EGF-EGFR_Signaling_Pathway_WP437_79266	0.29768857	3	162
Hs_Hair_Follicle_Development-_Organogenesis_(Part_2_of_3)_WP2839_78519	0.29892978	1	31
Hs_Oxidative_Stress_WP408_78546	0.29892978	1	30
Hs_Formation_of_Fibrin_Clot_(Clotting_Cascade)_WP1818_76965	0.29892978	1	30
Hs_Bladder_Cancer_WP2828_79529	0.30718276	1	31
Hs_Prostaglandin_Synthesis_and_Regulation_WP98_72088	0.30718276	1	31
Hs_Pregnane_X_Receptor_pathway_WP2876_79556	0.31533873	1	33
Hs_Mitochondrial_Protein_Import_WP2717_76934	0.31533873	1	32
Hs_Pregnane_X_Receptor_pathway_WP2876_79537	0.31533873	1	33
Hs_Assembly_of_collagen_fibrils_and_other_multimeric_structures_WP2798_77089	0.31533873	1	33
Hs_Meiotic_Synapsis_WP2731_76957	0.32339886	1	35
Hs_Alpha_6_Beta_4_signaling_pathway_WP244_78506	0.32339886	1	33
Hs_Monoamine_GPCRs_WP58_69046	0.32339886	1	34
Hs_Neural_Crest_Differentiation_WP2064_79263	0.3250018	2	101
Hs_Pre-NOTCH_Expression_and_Processing_WP2786_77076	0.33136424	1	38
Hs_Toll-like_receptor_signaling_pathway_WP75_72133	0.33788222	2	102
Hs_SRP-dependent_cotranslational_protein_targeting_to_membrane_WP2737_76970	0.34642774	2	111
Hs_Integrated_Cancer_pathway_WP1971_71249	0.34701523	1	36
Hs_Interleukin-3,_5_and_GM-CSF_signaling_WP1840_77073	0.34701523	1	37
Hs_Lipid_digestion,_mobilization,_and_transport_WP2764_77026	0.35470304	1	41
Hs_miR-targeted_genes_in_muscle_cell_-_TarBase_WP2005_78538	0.35726812	5	409
Hs_Influenza_Life_Cycle_WP2683_76880	0.36178118	3	217
Hs_Amyotrophic_lateral_sclerosis_(ALS)_WP2447_75221	0.36230046	1	38
Hs_FAS_pathway_and_Stress_induction_of_HSP_regulation_WP314_71366	0.36230046	1	38
Hs_G13_Signaling_Pathway_WP524_72112	0.36230046	1	38
Hs_Transport_of_inorganic_cations-anions_and_amino_acids-oligopeptides_WP1936_76845	0.36980858	1	43
Hs_Eukaryotic_Translation_Initiation_WP1812_76969	0.37602833	2	117
Hs_IL-5_Signaling_Pathway_WP127_78498	0.37722847	1	40
Hs_Mitotic_Prophase_WP2654_76823	0.37722847	1	44
Hs_Regulation_of_Lipid_Metabolism_by_Peroxisome_proliferator-activated_receptor_alpha_(PPARalpha)_WP2797_77088	0.38438606	2	118
Hs_tRNA_Aminoacylation_WP1938_76923	0.3918076	1	42
Hs_DNA_Replication_WP466_76196	0.3918076	1	42
Hs_Interferon_alpha-beta_signaling_WP1835_77078	0.3918076	1	45
Hs_IL-2_Signaling_Pathway_WP49_78543	0.3918076	1	42
Hs_Sphingolipid_metabolism_WP2788_77079	0.39896885	1	46
Hs_Hair_Follicle_Development-_Induction_(Part_1_of_3)_WP2804_78710	0.39896885	1	44
Hs_GPCR_ligand_binding_WP1825_76977	0.39899996	5	371
Hs_Host_Interactions_of_HIV_factors_WP2684_76883	0.4050616	2	137
Hs_Interleukin-11_Signaling_Pathway_WP2332_79525	0.40604594	1	44
Hs_Heart_Development_WP1591_78590	0.40604594	1	47
Hs_Aryl_Hydrocarbon_Receptor_Pathway_WP2873_79696	0.41303983	1	46
Hs_NCAM_signaling_for_neurite_out-growth_WP1866_76927	0.41303983	1	48
Hs_Aryl_Hydrocarbon_Receptor_pathway_WP2873_79544	0.41303983	1	46
Hs_Signalling_by_NGF_WP1976_76994	0.4267819	1	50
Hs_Fatty_acid,_triacylglycerol,_and_ketone_body_metabolism_WP1817_77087	0.44020277	1	49
Hs_miR-targeted_genes_in_leukocytes_-_TarBase_WP2003_78572	0.4414027	2	160
Hs_Complement_and_Coagulation_Cascades_WP558_67786	0.4533099	1	64
Hs_Vitamin_B12_Metabolism_WP1533_70117	0.4533099	1	53
Hs_Asparagine_N-linked_glycosylation_WP1785_78300	0.46611065	1	53
Hs_Interferon_type_I_signaling_pathways_WP585_79096	0.47239837	1	54
Hs_ErbB_Signaling_Pathway_WP673_69914	0.47239837	1	54
Hs_RANKL-RANK_Signaling_Pathway_WP2018_79274	0.47861212	1	55
Hs_Pathogenic_Escherichia_coli_infection_WP2272_78594	0.48475286	1	64
Hs_Glycerophospholipid_biosynthesis_WP2740_76974	0.4968185	1	59
Hs_Regulation_of_toll-like_receptor_signaling_pathway_WP1449_77378	0.49911815	2	150
Hs_Regulation_of_toll-like_receptor_signaling_pathway_WP1449_79550	0.5028287	2	150
Hs_Complement_and_Coagulation_Cascades_WP558_79680	0.5086021	1	61
Hs_Oxidative_phosphorylation_WP623_79520	0.5086021	1	63
Hs_Notch_Signaling_Pathway_WP61_78592	0.5143901	1	61
Hs_SREBP_signalling_WP1982_78494	0.52011013	1	65
Hs_Leptin_signaling_pathway_WP2034_79545	0.52011013	1	62
Hs_Metabolism_of_water-soluble_vitamins_and_cofactors_WP1857_76875	0.52011013	1	67
Hs_Mitotic_Metaphase_and_Anaphase_WP2757_77009	0.524715	2	153
Hs_Histone_Modifications_WP2369_69927	0.5313491	1	67
Hs_TSH_signaling_pathway_WP2032_78548	0.5423253	1	66
Hs_DNA_Damage_Response_WP707_78527	0.5530448	1	68
Hs_Peptide_GPCRs_WP24_79444	0.57373774	1	73
Hs_Integrated_Breast_Cancer_Pathway_WP1984_72732	0.57652223	2	164
Hs_miRNA_Regulation_of_DNA_Damage_Response_WP1530_79564	0.58372265	1	98
Hs_Membrane_Trafficking_WP1846_76873	0.58372265	1	78
Hs_miRNA_Regulation_of_DNA_Damage_Response_WP1530_78503	0.58372265	1	98
Hs_Prolactin_Signaling_Pathway_WP2037_78501	0.5934741	1	76
Hs_Metabolism_of_carbohydrates_WP1848_76833	0.6258427	1	87
Hs_miR-targeted_genes_in_epithelium_-_TarBase_WP2002_78530	0.6296174	3	345
Hs_Apoptosis_WP254_78808	0.63025177	1	84
Hs_Allograft_Rejection_WP2328_78554	0.6389149	1	100
Hs_Mitotic_G2-G2-M_phases_WP1859_77022	0.6431702	1	89
Hs_TCR_Signaling_Pathway_WP69_72111	0.6556379	1	92
Hs_Respiratory_electron_transport,_ATP_synthesis_by_chemiosmotic_coupling,_and_heat_production_by_uncoupling_proteins_WP1902_77091	0.6556379	1	94
Hs_B_Cell_Receptor_Signaling_Pathway_WP23_78566	0.6676707	1	94
Hs_DNA_Damage_Response_(only_ATM_dependent)_WP710_70109	0.67158765	1	97
Hs_Mitotic_Prometaphase_WP2652_76819	0.6754585	1	98
Hs_Electron_Transport_Chain_WP111_79220	0.6904916	1	104
Hs_GPCRs,_Class_A_Rhodopsin-like_WP455_79717	0.79803675	2	262
Hs_GPCRs,_Class_A_Rhodopsin-like_WP455_78510	0.79803675	2	262

Supplementary Table S13

Primer information used in the qRT-PCR

Gene name	Forward primer sequence (5'-3')	Reverse primer sequence (5'-3')
BUB1	TGGGACTGTTGATGCTCCAAACT	GTATTTGGATAGGAACTCACTGG
BUB1b	CAGCAGAAACGGGCATTT	TACCTATCCCAAACATCCAGAG
CCNB1	GGCCAAAATGCCTATGAAGA	AGATGTTTCCATTGGGCTTG
GAPDH	CATGAGAAGTATGACAACAGCCT	AGTCCTTCCACGATACCAAAGT
